# New indole-linked 1,2,4-triazole derivatives as dual FAK inhibitors and apoptosis inducers targeting survival and migration in triple-negative breast cancer *in-vitro*

**DOI:** 10.1038/s41598-026-41032-1

**Published:** 2026-04-22

**Authors:** Hayam A. Abd El Salam, Nourhan Abu-Shahba, Ghadha Ibrahim Fouad, Marwa Mahmoud, Eslam A. Mostafa, Mona A. M. Abozeid, Heba M. Abo-Salem, Rasha A. M. Azouz

**Affiliations:** 1 Green Chemistry Department, Organic Chemicals Industries Institute, National Research Centre, Giza, 12622 Egypt; 2 Department of Human Medical Molecular Genetics, Human Genetics and Genome Research Institute, National Research Centre, Giza, 12622 Egypt; 3 Stem Cell Research Lab., Medical Research Center of Excellence, National Research Centre, Giza, 12622 Egypt; 4 Therapeutic Chemistry Department, Pharmaceutical and Drug Industries Research Institute, National Research Centre, Giza, 12622 Egypt; 5 Department of Organometallic and Organometalloid Chemistry, Chemical Industries Research Institute, National Research Centre, Giza, 12622 Egypt; 6 Cancer Biology and Genetics Laboratory, Centre of Excellence for Advanced Sciences, National Research Centre, Giza, 12622 Egypt; 7 Genetics and Cytology Department, Biotechnology Research Institute, National Research Centre, Giza, 12622 Egypt; 8 Chemistry of Natural Compounds Department, Pharmaceutical and Drug Industries Research Institute, National Research Centre, Giza, 12622 Egypt; 9 Molecular Biology Department, Biotechnology Research Institute, National Research Centre, Giza, 12622 Egypt

**Keywords:** Indole, 1,2,4-Triazole, Triple-negative breast cancer, FAK inhibition, Cell migration, Pre-clinical Safety, Cancer, Cell biology, Drug discovery, Oncology

## Abstract

Focal adhesion kinase (FAK) is overexpressed and hyperactivated in triple-negative breast cancer, driving tumor aggressiveness and cancer stem cell–mediated therapy resistance. Therefore, targeting FAK signalling represents a promising therapeutic strategy. In this study, a series of indole and bis-indole-1,2,4-triazoles were synthesized and evaluated as anti-TNBC agents targeting FAK. Compounds **3c**, **4c**, and **5c** displayed potent cytotoxicity (IC₅₀ = 41–77 µg/mL) with minimal toxicity to normal cells, outperforming precursor compound** 2**. Wound-healing assay revealed significant inhibition of cell migration, particularly by **4c**. Cell cycle analysis revealed that **4c** induced S-phase arrest in MCF-7 cells and G1-phase arrest in MDA-MB-231 cells, accompanied by significant apoptosis. In MDA-MB-231 cells, **4c** triggered extensive total apoptosis (90.84%) with minimal necrosis. Gene expression studies demonstrated that **4c** markedly downregulated *PTK2* (FAK), *CCL5*, and *BCL2*, while upregulating *CASP3*, highlighting its dual role as FAK inhibitor and apoptosis inducer. Importantly, **4c** efficiently suppressed FAK protein expression (61.3%) in TNBC, compared to the FAK inhibitor GSK-2256098 (70.7%). In vivo toxicity assessment confirmed good tolerability in mice without profound hepatic or renal impairments, while docking and ADMET analyses confirmed strong FAK binding affinity, and favourable pharmacokinetics of **4c**. Collectively, **4c** emerges as a promising FAK-targeted candidate for TNBC therapy.

**Supplementary Information:**

The online version contains supplementary material available at 10.1038/s41598-026-41032-1.

## Introduction

Breast cancer (BC) is the second leading cause of cancer-related mortality among women, and about 1–3 million cases are diagnosed worldwide every year^1,2^. Triple-negative breast cancer (TNBC) is the most aggressive and therapy-resistant breast cancer form, represents 20% of total BC cases and mostly affects young women under 40 years old^3,4^. TNBC is characterized by the low/lack of expression of three key receptors, including HER2 (human epidermal growth factor receptor 2), PR (progesterone receptors) and ER (estrogen receptors) and therefore not sensitive to available hormonal targeted therapies^5^. Clinically, TNBC is often associated with high histological grade, poor prognosis, increased risk of recurrence, and metastatic spread at an early stage. TNBC metastasizing is highly aggressive and frequently metastasizes to the lungs and central nervous system. TNBC is a highly heterogeneous disease, and in vitro cell line models are commonly classified into distinct molecular subtypes based on their genetic, transcriptomic, and morphological characteristics. The most widely classification describes six TNBC subtypes, including Basal-like 1 (BL1), Basal-like 2 (BL2), Immunomodulatory (IM), Mesenchymal (M), Mesenchymal stem-like (MSL), and Luminal androgen receptor (LAR) subtypes^6–8^. The BL1 subtype is characterized by elevated expression of genes involved in cell-cycle regulation and DNA damage response, with representative models including MDA-MB-468, HCC1937, HCC2157, HCC1599, HCC1143, HCC3153, and HCC38^9^. BL2 tumors are enriched in growth-factor signaling pathways, such as EGFR and MET, and include cell lines such as SUM149PT, HCC1806, HCC70, CAL-851, and HDQ-P1^9^. Immunomodulatory (IM) subtype is associated with immune-related pathways and cytokine signaling, includes cell lines such as HCC1187 and DU4475^9^. The mesenchymal-like (M) subtype exhibits epithelial-to-mesenchymal transition (EMT) and enhanced cell motility, commonly represented by BT-549, CAL-51, and CAL-120^9^. The mesenchymal stem-like (MSL) subtype shares mesenchymal features but displays lower proliferative activity and higher angiogenic potential, with MDA-MB-231, HS578T, MDA-MB-157, SUM159PT, andMDA-MB-436 as typical models. The LAR subtype is driven by androgen receptor signaling and displays a luminal-like gene expression profile, with MDA-MB-453 considered a gold-standard model, in addition to SUM185PE, HCC2185, CAL-148, and MFM-223 models^9^. Collectively, these aggressive biological features contribute to limited therapeutic options, poor treatment outcomes, and reduced overall survival in patients with TNBC^10^. Currently, no targeted therapies are available for TNBC, and the most popular therapeutic options are the standard cytotoxic chemotherapies with significant side effects, lack of long-term efficacy, and tendency for rapid resistance development^5,11^. Hence, there is an urgent need for novel, effective, selective and safe treatment alternatives with recognized mechanism(s) of action.

Focal adhesion kinase (FAK) is a cytoplasmic tyrosine kinase that plays a vital role in regulating tumor development processes such as invasion, metastasis, and angiogenesis through both kinase-dependent and kinase-independent pathways^12,13^. Notably, FAK is frequently overexpressed in various human cancers, including TNBC cells, where its elevated levels are associated with enhanced invasion and metastasis^14^. Consequently, FAK inhibitors are emerging as one of the most promising targeted therapeutic approaches for TNBC treatment^12^. Several five-membered heterocyclic compounds, including oxadiazoles, triazoles, and thiadiazoles, have been identified as potent FAK inhibitors with significant anti-proliferative activity^15–17^.

1,2,4-Triazole derivatives have attracted renewed attention from organic and medicinal chemists, as several new hybrids with broader biological activity have recently been developed^18–20^. These compounds serve as key pharmacophores, interacting strongly with biological receptors due to their dipole moment, ability to form hydrogen bonds, good solubility, and resistance to both chemical and metabolic degradation^21^. Recently, four 1,2,4-triazole derivatives were synthesized, demonstrating potent anti-proliferative effects and significantly inhibiting FAK kinase at nanomolar concentrations^22^.

Moreover, indoles have gained significant interest due to their synthetic versatility and notable biological relevance, particularly in cancer research^23^. Interestingly, numerous studies indicate that conjugating indole with 1,2,4-triazole could significantly boost their anticancer activity^24–26^. In this context and as part of our continuing efforts to identify novel bioactive anticancer agents^20,27–30^, the present study aimed to design, synthesize, and characterize a new series of indole and bis-indole-linked 1,2,4-triazole derivatives, evaluating their in vitro anticancer potential against breast cancer cell lines, with special emphasis on triple-negative breast cancer (MDA-MB-231). The research investigated the possible mechanism underlying their cytotoxic action through cell-cycle arrest, apoptosis induction, and FAK inhibition. In addition, the selectivity and preliminary safety profile of the most active compounds, through in vitro and in vivo assays alongside in silico studies, was investigated.

## Result and discussion

### Chemistry

**Fig. 1:**
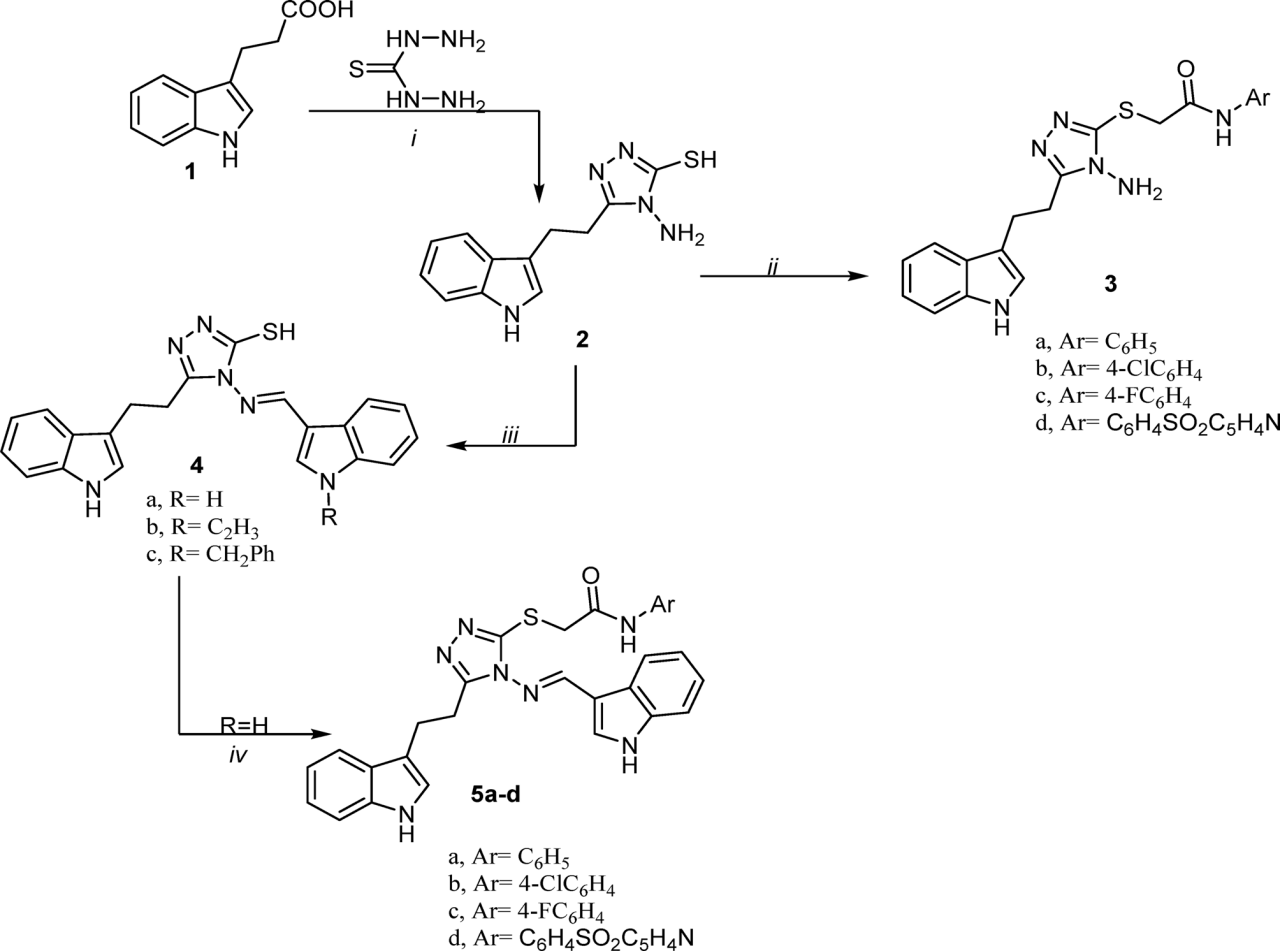
Synthesis of 1,2,4-triazole derivatives 2, 3a-d, 4a-c and 5a-d, reagent and condition: (i) oil bath, fusion at 130–140 °C for 4h; (ii) chloro acetamide derivatives, acetone, K_2_CO_3_, reflux; (iii) *N*-substituted indole-3-carboxaldehydes, EtOH/AcOH, refluxß (iv) chloro acetamide derivatives, acetone, K_2_CO_3_, reflux.

The synthesis of new indole-linked 1,2,4-triazole derivatives **3–8** was accomplished according to the synthetic routes illustrated in Fig. 1. Initially, the key precursor 5-(2-(1*H*-indol-3-yl)ethyl)-4-amino-4*H*-1,2,4-triazole-3-thiol (**2**) was synthesized through a straightforward one-pot fusion reaction between indole-3-propionic acid (**1**) and thiocarbohydrazide. Subsequently, the thiol (SH) functionality of compound **2** was subjected to S-alkylation reaction with various chloroacetamide derivatives, namely 2-chloro-*N*-phenylacetamide, 2-chloro-*N*-(4-chlorophenyl)acetamide, 2-chloro-*N*-(4-fluorophenyl)acetamide and 2-chloro-*N*-(4-(*N*-(pyridin-2-yl)sulfamoyl)phenyl)acetamide, which afforded the corresponding *S*-acetamide derivatives **3a-d** (Fig. 1).

The 1HNMR spectra of **3a-d** confirmed successful S-alkylation by the disappearance of the SH proton signal and the appearance of new singlet signals at δ 10.59–10.05 ppm, and δ 4.17–4.05 ppm (integrating for two protons), corresponding to NH and SCH₂-protons, respectively. For instance, the ^1^HNMR spectrum of **3a** in DMSO-d₆ displayed four singlet signals at δ 10.77, 10.31, 5.93, and 4.06 assigned to 2NH, NH₂, and CH₂ protons, respectively. Additionally, multiplet signals observed at δ 3.05 ppm were assigned to the ethylene (CH₂–CH₂) protons linking the indole and triazole rings, along with characteristic aromatic proton resonances. The ^13^C NMR spectrum of **3a** (DMSO-d6) further supported the proposed structure, showing three distinct CH₂ carbon signals at δ 39.91, 25.48, and 22.81 ppm, in addition to the expected aromatic carbon resonances (see Experimental Section).

Furthermore, a series of bis-indolyl-triazole Schiff bases **4a-c** was synthesized through acid-catalyzed condensation reaction of compound **2** with indole-3-carboxaldehyde, *N*-ethyl and/or *N*-benzyl indole-3-carboxaldehydes under reflux in absolute ethanol (Fig. 1).

The ^1^HNMR spectra of compounds **4a–c** confirmed Schiff base formation by the disappearance of the NH₂ proton signals and the appearance of new singlet resonances in the range δ 13.62–9.46 ppm, corresponding to SH, NH, and azomethine (CH=N) protons. For example, the ^1^HNMR spectrum of compound **4b** (DMSO-d₆) displayed three singlet characteristic singlet signals at δ 13.62, 10.78 and 9.50 ppm which were attributed to SH, NH and CH=N protons, respectively. Additionally, quartet and triplet signals at δ 4.23 and 1.38 ppm were assigned to the NCH₂CH₃ protons, respectively, while signals observed at δ 3.07–3.06 ppm corresponded to the two methylene (CH₂) groups. The aromatic proton resonances were observed in the expected region. The ^13^C NMR spectrum (DMSO-d6) of **4b** further supported the proposed structure, displaying CH₂ and CH₃ carbon signals at δ 50.69, 26.65, 22.43, and 15.69 ppm, alongside signals from aromatic carbons (Ar–C) (see Experimental Section).

In a subsequent synthetic route, S-alkylation of the bis-indolyl Schiff bases **4a–c** with the different chloroacetamide derivatives, namely 2-chloro-*N*-phenylacetamide, 2-chloro-*N*-(4-chlorophenyl)acetamide, 2-chloro-*N*-(4-fluorophenyl) acetamide and 2-chloro-*N*-(4-(*N*-(pyridin-2-yl)sulfamoyl)phenyl)acetamide] yielded the corresponding bis-indolyl *S*-acetamide derivatives **5a-d** (Fig. 1).

The ^1^HNMR spectra confirmed successful derivatization by the disappearance of SH signals and the appearance of new singlets for SCH₂ protons at δ 4.04–4.95 ppm. For instance, the ^1^HNMR spectra of compound **5a** displayed characteristic singlet signals at δ 11.15, 10.81, 10.44, 8.66, and 4.95 ppm, corresponding to NH, azomethine (CH=N), and SCH₂ protons, respectively, in addition to multiplet signals at δ 3.07–2.99 assigned to the ethylene (CH₂–CH₂) linker and the expected aromatic proton signals. The ^13^CNMR spectrum of **5a** further supported the proposed structure, displaying CH₂ carbon signals at δ 53.85, 25.04, and 22.91 ppm, consistent with the proposed structures (see Experimental Section).

### Biological activities

#### Cytotoxic activity

**Fig. 2:**
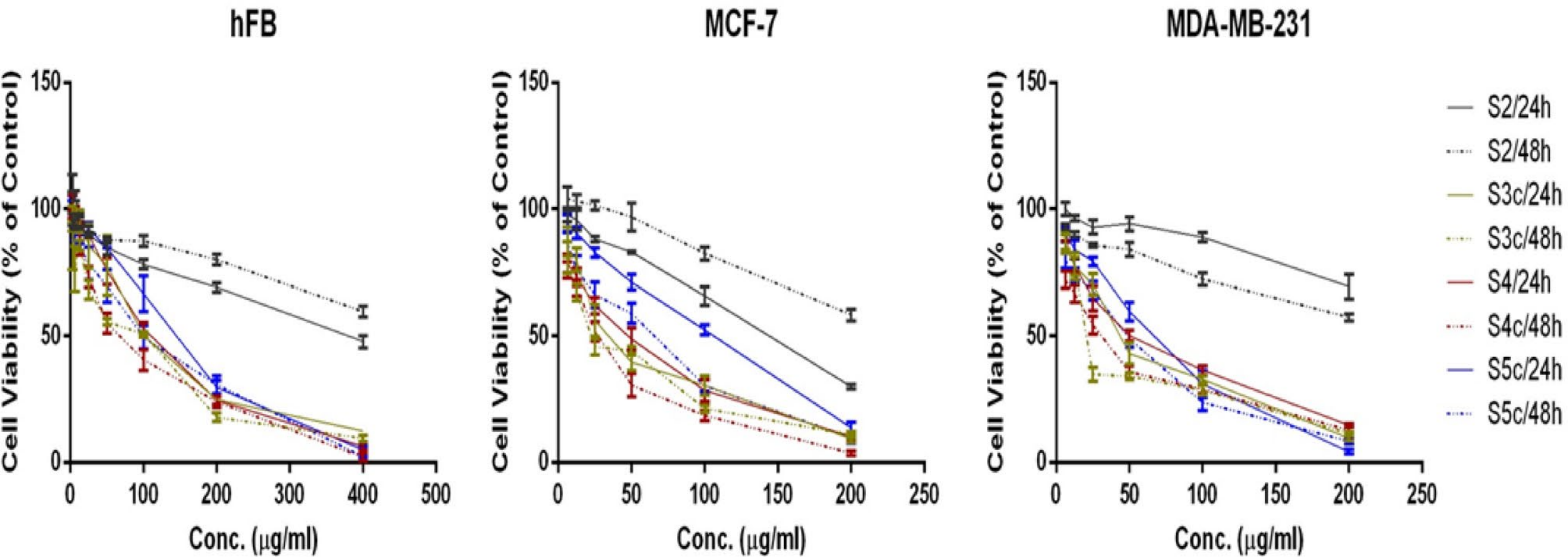
Evaluation of cytotoxic response of compounds 2, 3c, 4c, and 5c on hFB, MCF-7, and MDA-MB-231 cell lines after treatment with compounds 2, 3c, 4c, and 5c for 24h and 48h. Data are presented as mean ± SEM (n = 3).

The cytotoxic activities of the newly synthesized 1,2,4-triazole derivatives were evaluated against two human breast cancer cell lines (MCF-7 and MDA-MB-231) and a normal human fibroblast cell line (hFB) using the MTT assay after 24 h and 48 h of exposure. The half-maximal inhibitory concentration (IC_50_) values were summarized in Table 1. Overall, several derivatives demonstrated enhanced cytotoxicity compared to the parent compound **2**, which exhibited relatively weak activity with IC_50_ values above 140 µg/mL on MCF-7 cells and more than 230 µg/mL on MDA-MB-231 cells after both incubation periods. This observation indicates that structural modification significantly improved the anti-proliferative potential of the synthesized analogues. Among the tested compounds, 1,2,4-triazole derivatives **3c, 4c,** and **5c** displayed the most potent activities with IC₅₀ values in the range of **41–78 µg/mL** against both MCF-7 and MDA-MB-231 cell lines after 48 h of exposure (Fig. 2). Notably, compounds **3c, 4c** and **5c** demonstrated lower toxicity toward normal fibroblasts (IC₅₀ ≈ 130–183 µg/mL), suggesting a favorable selectivity profile. While, derivatives **3a**, **3b**, **4a**, **5a**, **5b**, and **5d** exhibited moderate cytotoxicity with IC_50_ values in the range of 50–99 µg/mL against both MCF-7 and MDA-MB-231 cell lines after 48 h. On the other hand, compounds **3d**, **4b**, and **5e** showed weak cytotoxicity with IC_50_ > 140 µg/mL. A time-dependent increase in activity was evident for most active derivatives, with lower IC_50_ values observed after 48 h compared to 24 h, indicating that prolonged exposure enhances cytotoxic efficacy. Overall, **3c** and **4c** emerged as the most promising derivatives due to their potent and selective anti-proliferative effects.

**Table 1:** The IC_50_ concentration values of the tested derivatives on the different cell lines after 24h and 48h.

Compound No.	MCF-7		MDA-MB-231		hFB	
	24h	48h	24h	48h	24h	48h
**2**	142.96	235.75	348.31	235.75	368.63	475.94
**3a**	81.65	75.09	90.03	50.44		
**3b**	129.73	67.23	154.42	91.94		
**3c**	65.15	51.73	74.34	51.21	175.45	130.15
**3d**	159.87	157.99	227.33	300.30		
**4a**	63.17	93.36	72.55	51.09		
**4b**	309.59	340.62	406.67	380.91		
**4c**	66.07	41.41	81.28	48.22	172.13	141.27
**5a**	104.67	99.47	140.10	74.26		
**5b**	73.36	67.62	76.66	50.83		
**5c**	108.28	77.61	83.41	66.64	182.72	158.31
**5d**	127.40	83.57	135.10	96.33		
**5e**	140.27	144.45	209.15	223.47

#### Wound healing assay

**Fig. 3:**
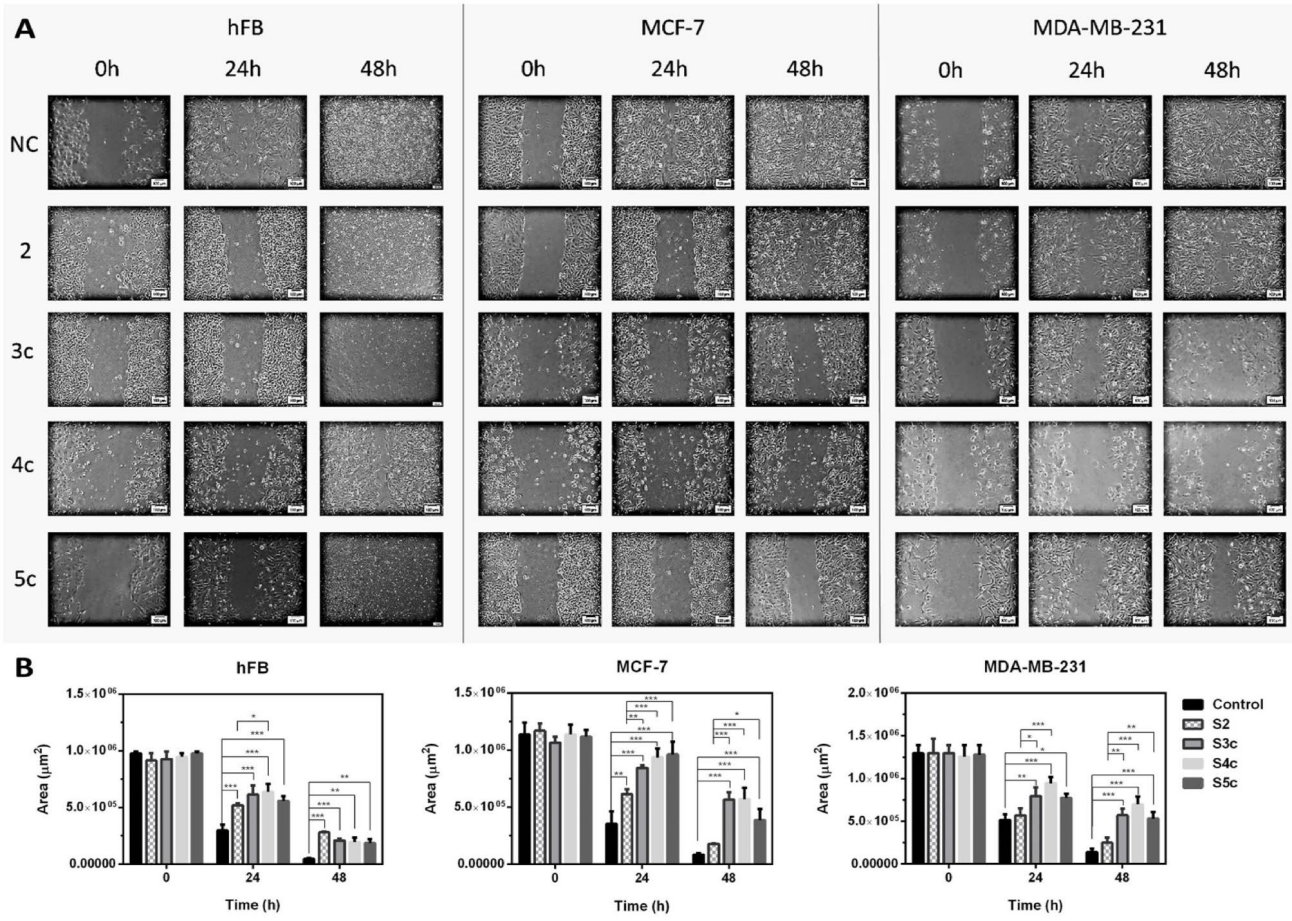
Wound healing assay of hFB, MCF-7, and MDA-MB-231 cell lines after treatment with compounds 2, 3c, 4c, and 5c by half the inhibitory concentration 50 (IC_25_) regarding to non-treated cells (NC) for different time intervals (0, 24, and 48 h.). (**A**) Representative photo panel for the scratched areas of the treated cell lines at different time points with scale bar 100 µm. (**B**) Scoring the closure rate of gap area of migration (µm^2^) regarding to the time (h) in different treatments. Data are presented as mean ± SEM (n = 3 independent biological replicates), and the significance variances were calculated regarding to control or the parent compound; * p < 0.05, ** p < 0.01, and *** p < 0.001.

Wound healing assay was conducted to evaluate the cell migration response to a scratched region (Fig. 3A). Migration areas were quantified in µm^2^ to compare the effects of compounds **2**, **3c**, **4c**, and **5c** against non-treated cells. Breast cancer cell lines (MCF-7 and MDA-MB-231) and human skin fibroblast (hFB) cells were exposed to half the IC_50_ concentration (IC_25_) of the tested compounds for 48 h to identify the most potent compounds to inhibit cell migration effectively (Fig. 3B).

The results illustrated that the delay in cell migration increased with time line, and the derivative compounds (S3c, S4c, and S5c) inhibited cell migration significantly at the same time point compared to the non-treated cells, or the parental compound **2**. For hFB cells, compounds **4c** and **5c** declined cell migration remarkably at p < 0.01, and compounds **2** and **3c** inhibited cell migration highly significantly at P < 0.001 in comparison to non-treated cells after 48h. For MCF-7 and MDA-MB-231 cells, the potential of derivative compounds (**3c**, **4c**, and **5c**) to inhibit closure of the scratched areas was delayed highly significantly (p < 0.001) from the initial time points compared to non-treated cells or cells treated with the principal compound **2**.

The most active 1,2,4-triazole derivatives **3c**, **4c**, and **5c** were further investigated for their cytotoxic mechanisms of action at 37.5 µg/mL for 48 h, in comparison to the parent compound **2**.

#### Cell cycle analysis

**Fig. 4:**
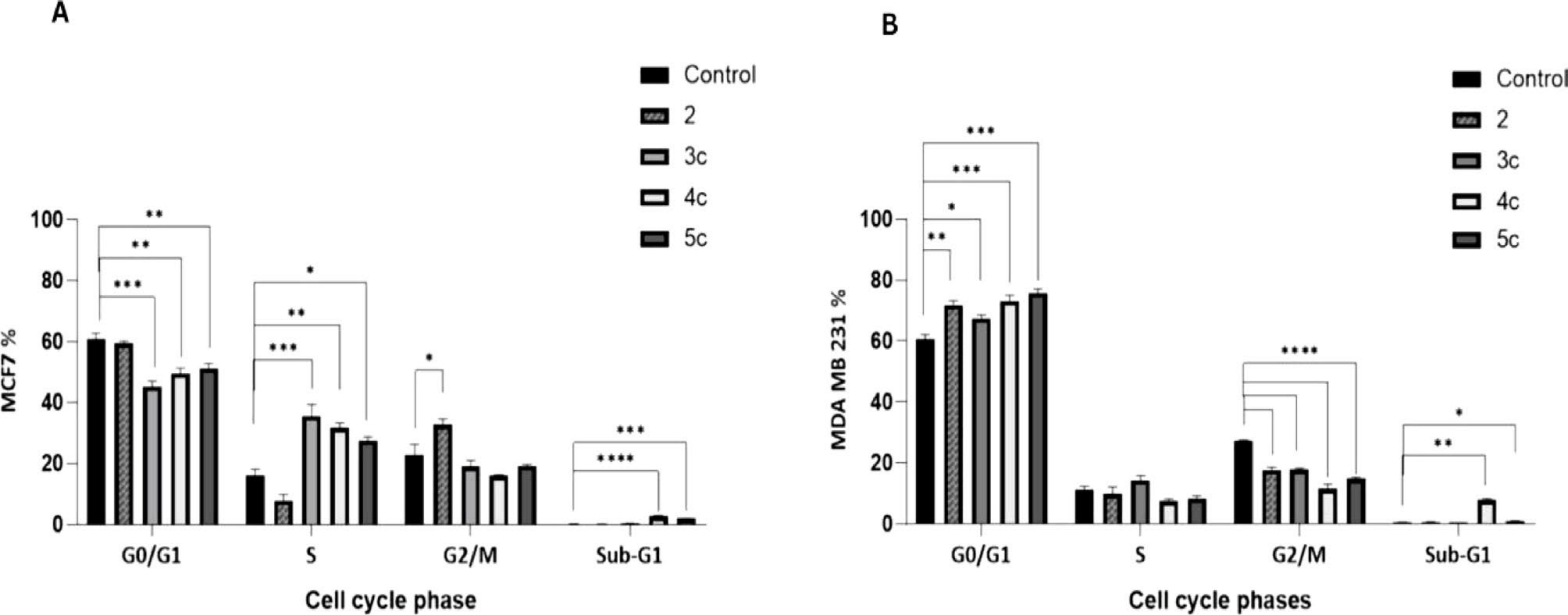
Cell Cycle analysis of MCF7 and MDA-MB-231 cells. (**A**) It represents MCF7 cell frequencies among the cell cycle phases (G0/G1, S, and G2/M) in the different treatment conditions. (**B**) It represents MDA-MB-231 cell frequencies among the cell cycle phases in the different treatment conditions. Data are presented as mean ± SEM (n = 3 independent biological replicates), * 0.05 ≥ P ≥ 0.01; **: 0.01 > P ≥ 0.001; *** P < 0.001, **** < 0.0001. One-way ANOVA followed by Bonferroni’s multiple comparisons test was used for statistical analysis.

Both MCF-7 and MDA-MB-231 cells responded differently to compounds **2**, **3c**, **4c**, and **5c** (37.5 µg/mL, 48 h). In MCF-7 cells, untreated controls displayed typical G1 predominance with minimal sub-G1 apoptosis. Compound **2** significantly reduced S-phase cells while increasing G2/M fraction (G2/M arrest), without substantial sub-G1 induction. Compound **4c** prominently arrested cells at S-phase with marked apoptosis, while **3c** and **5c** induced S-phase arrest with minimal **3c** and moderate **5c** apoptosis, respectively (Fig. 4A). In MDA-MB-231 cells, all four compounds significantly arrested cells at the G1 phase (limiting DNA synthesis and mitosis), with **3c** showing the weakest effect. Compounds **4c** and **5c** markedly elevated sub-G1 populations (apoptosis), with **4c** demonstrating superior potency (Fig. 4B, Supp. Table 2).

#### Apoptosis induction (annexin V/PI)

**Fig. 5:**
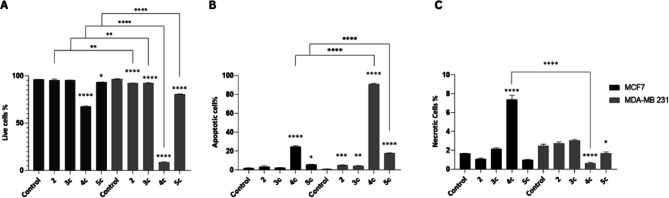
Annexin V-FITC/PI flow cytometric analysis of apoptosis in MCF-7 and MDA-MB-231 cells. The figure shows the percentage of (**A**) viable cells (Annexin V − /PI −), (**B**) early apoptotic (Annexin V + /PI −) and late apoptosis/secondary necrosis (Annexin V + /PI +) populations, and (**C**) primary necrotic cells (Annexin V − /PI +) under the indicated treatment conditions in both cancer cell lines. Data are presented as mean ± SEM (n = 3 independent biological replicates). Statistical significance indicators above the bars represent comparisons between each treated group and the corresponding untreated control, * 0.05 ≥ P ≥ 0.01; ** 0.01 > P ≥ 0.001; *** P < 0.001; **** P < 0.0001. Statistical analysis was performed using one-way ANOVA followed by Bonferroni’s multiple comparisons test.

Supporting the sub-G1 cell cycle data, treatment with compound **4c** significantly reduced the percentage of viable MCF-7 cells compared with untreated controls or compound **2** (Fig. 5A). This reduction was accompanied by a significant increase in early apoptotic (Annexin V + /PI −) and late apoptosis/secondary necrosis (Annexin V + /PI +) cell populations, in addition to a modest elevation in necrotic cells (Annexin V − /PI +) (Fig. 5B,C, Supp. Table 3). Compound **5c** moderately but significantly, increased early apoptotic and late apoptosis/secondary necrosis populations in MCF-7 cells, whereas compounds **2** and **3c** exerted negligible effects (Fig. 5B).

In MDA-MB-231 (TNBC) cells, compound 4c demonstrated markedly stronger pro-apoptotic activity. The percentage of viable cells decreased from 96.56% (control) to 8.54% (Fig. 5A). Early apoptotic cells (Annexin V + /PI −) increased dramatically from 0.4% to 62.04%, while the late apoptosis/secondary necrosis population (Annexin V + /PI +) increased from 0.58% to 28.8%, yielding a total apoptotic of 90.84% (Fig. 5B, Supp. Table 3). In contrast, primary necrotic cells (Annexin V − /PI +) remained minimal (0.62%) (Fig. 5C).

Compound **5c** induced apoptosis in MDA-MB-231 cells, although to a lesser extent (total apoptosis: 17.85%), with early apoptosis increasing to 13.44% and late apoptosis/secondary necrosis to 3.86%, accompanied by a reduction in viable cells to 80.4%. Compounds **2** and **3c** exhibited mild apoptotic effects (5.2% and 4.6% total apoptosis, respectively).

Thus, **4c** most potently drives TNBC apoptosis, with **5c** intermediate between mild **2**/**3c** effects and **4c’s** extreme potency, consistent with prior reports of 1,2,4-triazolo-indolyl conjugates in TNBC models. The synthetic derivatives, particularly **4c**, effectively arrested cell cycle progression in the TNBC MDA-MB-231 cell line at G1 phase while potently inducing programmed cell death (90.84% total apoptosis), thereby inhibiting tumor cell proliferation and migration. These findings align with previous reports demonstrating the cell cycle arrest and ROS-mediated apoptosis induction by 1,2,4-triazolo-linked indolyl conjugates across multiple cancer cell lines, including TNBC MDA-MB-231 models^31^.

#### Gene expression dysregulation

FAK is widely implicated in cancer growth, survival, motility, and drug resistance across multiple malignancies^32^. In breast cancer, FAK expression is elevated in several tumor cell lines, including the TNBC model MDA-MB-231, acting as a key player in mesenchymal cell migration^33,34^.

**Fig. 6:**
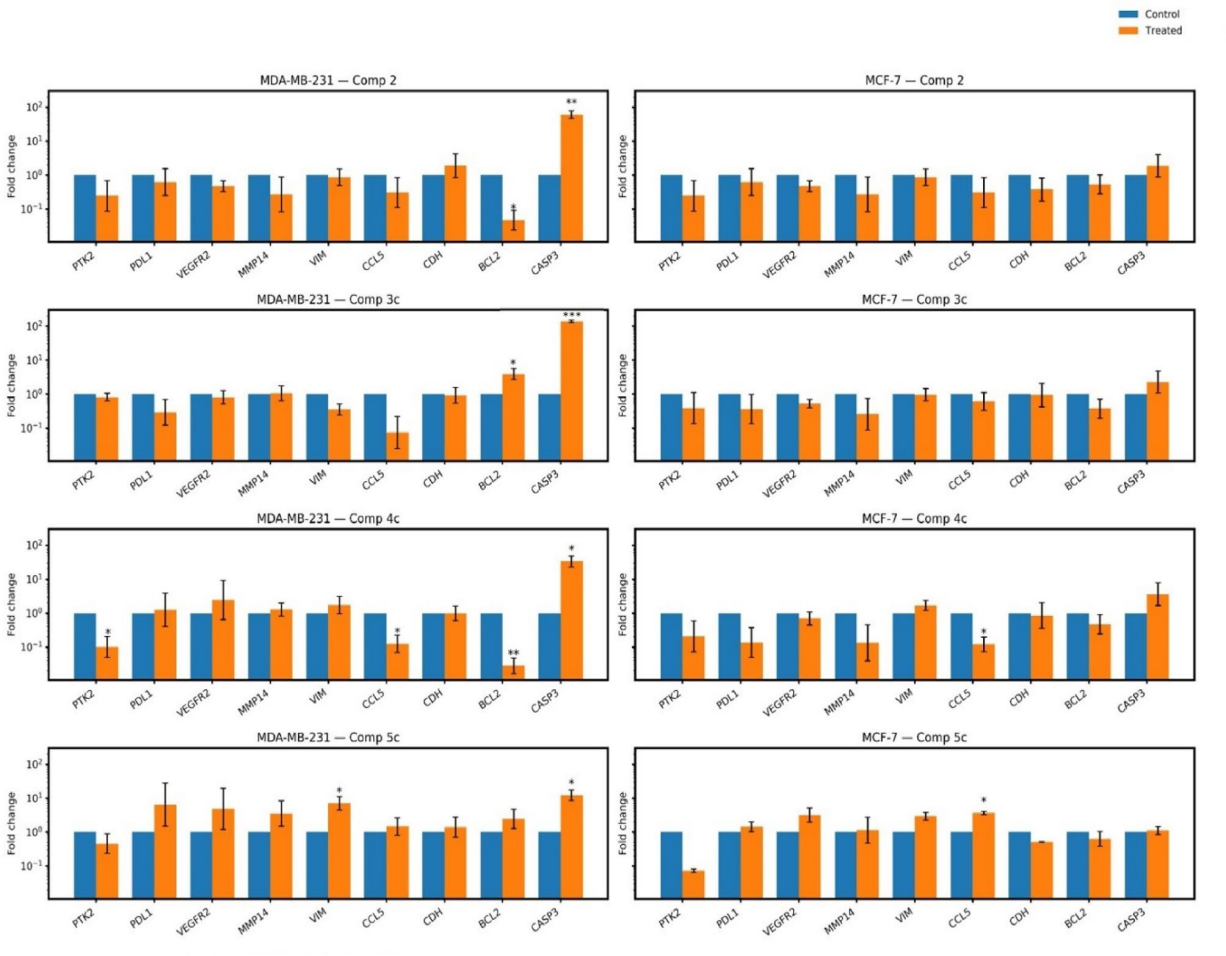
RT-qPCR results of FAK-related genes in breast cancer cell lines following treatment with the most active compounds. The figure shows the expression levels of *PTK2* (FAK), *PDL, VEGFR2, VIM, CCL5, CDH1, BCL2,* and *CASP3* in MDA-MB-231 and MCF-7 cells that were treated with the parent compound (2) and the selected active derivatives (3c, 4c, and 5c) alongside untreated controls. Relative expression is presented as fold change calculated by the comparative method (2^−ΔΔCt^) normalized to GAPDH. Log scale was used to visualize both up- and downregulation. Data are represented as mean ± SEM of three independent biological replicates (n = 3), *p ≤ 0.05, **p ≤ 0.01, ***p ≤ 0.001.

Consequently, targeting FAK/*PTK2* expression and connected genes has emerged as an effective strategy for inhibiting tumor survival, immunosuppression, and migration/metastasis-associated programs^32,35^. Accordingly, we evaluated the effect of the most active synthesized indole-linked 1,2,4-triazole derivatives on the expression of the FAK gene (*PTK2*) and selected downstream functional readouts. Using RT-qPCR, we quantified mRNA expression levels of *PTK2*, apoptosis-linked genes (*BCL2* and *CASP3*), and migration/invasion-linked genes (*CCL5, vimentin, and MMP14*) across two breast cancer cell lines: MDA-MB-231 and MCF-7. Our results demonstrated that the parent compound **2** modulated the expression of apoptosis-related genes in the MDA-MB-231 cell line, markedly decreasing *BCL2* (anti-apoptotic) and increasing *CASP3* (pro-apoptotic) expression, while showing no significant effect in the MCF-7 cell line. Compound **3c** significantly upregulated the levels of both *BCL2* and *CASP3* in MDA-MB-231 cells, without any observable impact on MCF-7 cells. Notably, compound **4c** markedly downregulated the mRNA expression levels of *PTK2* (direct on-target inhibition of FAK), together with strong downregulation of the pro-tumor chemokine *CCL5*, and the anti-apoptotic regulator *BCL2*, while significantly raising the levels of the proapoptotic mediator *CASP3* in the MDA-MB-231 cells. Interestingly, compound **4c** also induced a significant suppression of *CCL5* expression level in MCF-7 cells. In contrast, compound **5c** significantly elevated *VIM* and *CASP3* in the MDA-MB-231 cells and enhanced *CCL5* expression levels in the MCF-7 cell line (Fig. 6). Collectively, these findings suggest that compound **4c** exhibits a strong inhibitory potential against mRNA expression of FAK/*PTK2* and induces concomitant changes in apoptosis- and migration-related transcripts in MDA-MB-231 cells. This is consistent with the findings of Chen et al.^33^, who demonstrated that their newly developed FAK inhibitor activated caspase-3, promoting the formation of cleaved caspase-3. They also reported a dose-dependent inhibition of **Bcl-xL** (a *BCL2* family protein) by their FAK inhibitor, further supporting its apoptotic activity. Furthermore, the downregulated expression of *CCL5* by compound **4c** in MDA-MB 231 can be correlated with the downregulation of FAK/PTK2, as it was previously reported that silenced FAK reduces *CCL5* expression levels, indicating that FAK is a key regulator of *CCL5* expression^36^. Hence, downregulation of *CCL5* is in line with the FAK inhibitory action of our **4c** compound. Importantly, in breast cancer models, CCL5 was found to increase the migration/invasion process^37^ downregulation of CCL5 is associated with decreased cell migration, as observed in our study. Also, it can be deduced that compound **4c** suppressed FAK-induced tumour immune evasion, which is mediated by *CCL5*^38^. However, mechanistic studies are required to establish causality.

#### FAK protein levels reduction

**Fig. 7:**
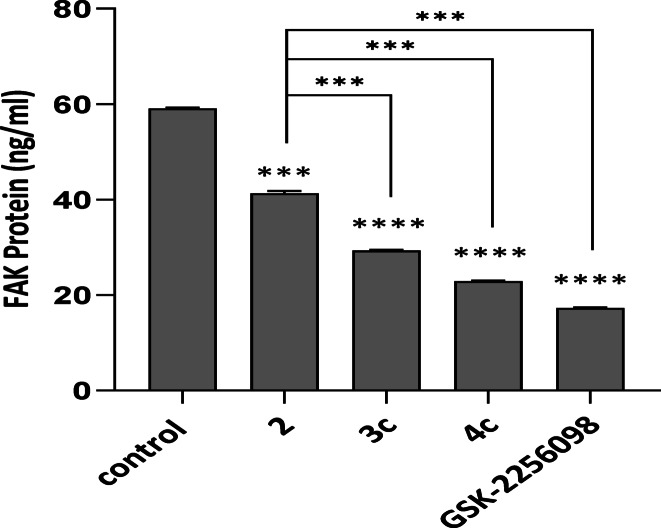
Effect of compounds 2, 3c, and 4c on FAK protein levels in MDA-MB-231 cells Welch’s t test was sued for statistical pairwise comparison. Abbreviations: FAK: Focal adhesion kinase, GSK-2256098: A reference FAK inhibitor.

FAK protein levels were quantified in MDA-MB-231 breast cancer cells following treatment with the IC_50_ concentration of the test compounds using the Human FAK SimpleStep ELISA® Kit (Abcam, ab187395). The highest FAK level (59.09 ± 2.29 ng/mL) was detected in the control untreated cells (Table 2, Fig. 7). Meanwhile, treatment with the compound **4c** significantly reduced FAK levels to 22.88 ± 0.88 ng/mL, representing a 61.3% inhibition relative to the control. 4c-mediated reduction of FAK levels was comparable to that induced by the reference FAK inhibitor GSK-2256098, which lowered FAK expression to 17.28 ± 0.67 ng/mL, corresponding to 70.7% inhibition. As well, compound **3c** treatment significantly decreased FAK levels to 29.33 ± 1.13 ng/mL (50.4% inhibition), while compound **2** caused a moderately significant reduction, resulting in a FAK concentration of 41.31 ± 1.60 ng/mL (30.1% inhibition) (Fig. 7, Table 2). Overall, all tested compounds suppressed FAK expression in MDA-MB-231 cells in a concentration-dependent manner, with compound **4c** showing the most potent inhibitory activity, followed by compounds **3c** and **2**. These findings suggest that the synthesized compounds, especially **4c**, may serve as effective modulators of the FAK pathway comparable to the standard inhibitor.

**Table 2:** FAK protein levels (ng/mL ± SD) in MDA-MB-231 cells following treatment with selected compounds 2, **3c** and **4c**.

Compound No.	FAK protein level (ng/ml)
**2**	41.311 ± 1.60
**3c**	29.330 ± 1.13
**4c**	22.880 ± 0.88
GSK-2256098	17.276 ± 0.67
Control	59.090 ± 2.29

From all the above-mentioned in vitro studies, we suggest that the decline of cell migration together with inhibition of gene and protein expressions of CCL5 and FAK markers might contribute, remarkably, to the anti-migratory potential of the investigated compounds on breast cancer cell lines and, in particular, the TNBC cells. These findings are in agreement with the previous reports that illustrated the direct correlation between cell migration and the protein expressions of chemotactic CCL5^39^, or focal adhesion kinase (FAK) protein markers^40^, which play crucial roles in cell migration, invasion, and metastatic pathways. Furthermore, the new indole-linked 1,2,4-triazole derivatives activated the programmed cell death pathways on the tested cancer cell line (MCF-7 and MDA-MB-231 cells) through inhibition of oncogenic markers BCL2, CCL5 and FAK and activation of apoptotic markers CASP3 and VIM pathways remarkably. This apoptotic response could result from the release of ROS from the indole-linked 1,2,4-triazole derivatives, which enhanced cell cycle arrest remarkably in both breast cancer cell lines. These observations agree with previous reports on numerous cancer cell lines, including TNBC MDA-MB-231 cells, which exhibited apoptotic response, cell cycle arrest and potential release of ROS after treatment with 1,2,4-triazolo-linked-indolyl conjugates^31^.

#### Structure–activity relationship (SAR) analysis

A comparative SAR analysis of the most active compounds **3c**, **4c** and **5c** revealed key structural features likely influencing their biological activity. Compound **4c** exhibited the highest potency, which can be attributed to its bis-indole framework connected through a 1,2,4-triazole ring bearing a thiol group. The dual indole units enhance aromatic interactions, while the triazole and thiol functionalities facilitate hydrogen bonding and sulfur-mediated binding. A benzyl substituent further improved lipophilicity and molecular flexibility, supporting effective target engagement. Compound **3c**, with a single indole and a para-fluorophenyl thioamide substituent, retained essential pharmacophores but shows reduced activity, likely due to lower aromatic density despite beneficial electronic effects from fluorine. Compound 5c displayed increased steric hindrance and structural rigidity arising from extended substitution around the triazole–thioamide region. Overall, these findings emphasize the critical role of enhanced aromaticity, balanced molecular flexibility, and accessible sulfur-containing functionalities in maximizing biological activity, with compound **4c** representing the most favorable structural configuration within this series.

#### In vivo toxicity study

Administration of a single intraperitoneal dose of the synthesized chemical compound of 12.5, 25, and 50 mg/kg to mice showed no clinical toxicity signs, including behavioral alterations or mortality in the following 48 h.

##### The impact of the chemical compound 4c on the histopathological differences in

- Liver

**Fig. 8:**
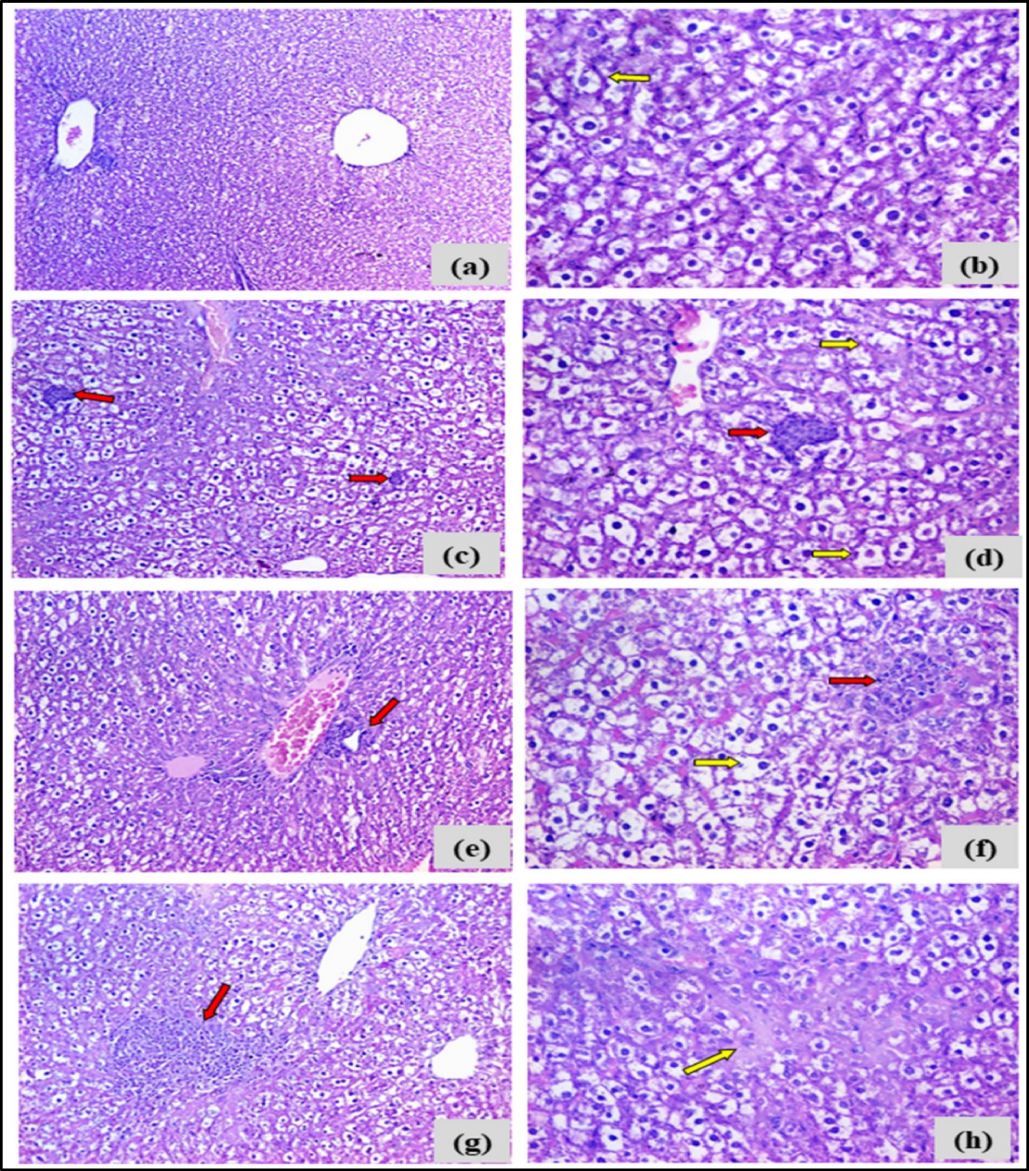
The histopathological findings of the hepatic tissues of different experimental groups receiving a single intraperitoneal dose of the synthesized compound 4c (12.5, 25, and 50 mg/kg), as compared to the negative control group. Photomicrographs (a and b): Sections in the liver of negative control mice showing regular hepatic lobular architecture with mild diffused cloudy swelling of hepatocytes (yellow arrows) (left H&E stain X100; right H&E stain, X400). Photomicrographs (c and d): Sections in the liver of 12.5 mg 4c-treated mice showing preserved hepatic lobular architecture with moderate diffuse hydropic degeneration of hepatocytes (yellow arrow) and focal intra-lobular aggregates of neutrophils (red arrows) (H&E stain, left X100; right, X400). Photomicrographs (e and f): Sections in the liver of 25 mg 4c-treated mice showing moderate diffuse hydropic degeneration of hepatocytes (yellow arrow) and focal portal and intra-lobular aggregates of neutrophils (red arrows) (H&E stain, left X100; right, X400). Photomicrographs (g and h) sections in the liver of 50 mg 4c-treated mice; showing dense focal intra-lobular aggregates of neutrophils (red arrows) with patchy hepatocellular necrosis (H&E stain, left X200; right, X400).

Liver tissues of the negative control group: Demonstrated regular hepatic architecture with mild cloudy swelling of hepatocytes. Liver tissues of 12.5 mg-treated group: Displayed moderate hydropic degeneration and mild intra-lobular neutrophilic infiltration. Liver tissues of the 25 mg treated group: Revealed similar hepatocellular degeneration with more extensive neutrophilic infiltration involving both portal and intra-lobular areas. Liver tissues of the 50 mg treated group: Showed marked histopathological damage, including dense neutrophilic infiltration and patchy necrosis (Fig. 8, Table 3).

**Table 3:** Comparative table demonstrating the histopathological changes in the hepatic tissues of mice administrated a single intraperitoneal dose of the synthesized compound **4c** (12.5, 25, and 50 mg/kg).

Group	Hepatic architecture	Hepatocyte changes	Inflammatory infiltration
Negative Control	Regular lobular	Mild cloudy swelling	None
12.5 mg/kg/one day	Preserved lobular	Moderate hydropic degeneration	Focal intra-lobular neutrophils
25 mg/kg/one day	Preserved lobular	Moderate hydropic degeneration	Portal and intra-lobular neutrophils
50 mg/kg/one day	Altered (focal necrosis)	Patchy necrosis	Dense intra-lobular neutrophils

b. Kidney

**Fig. 9:**
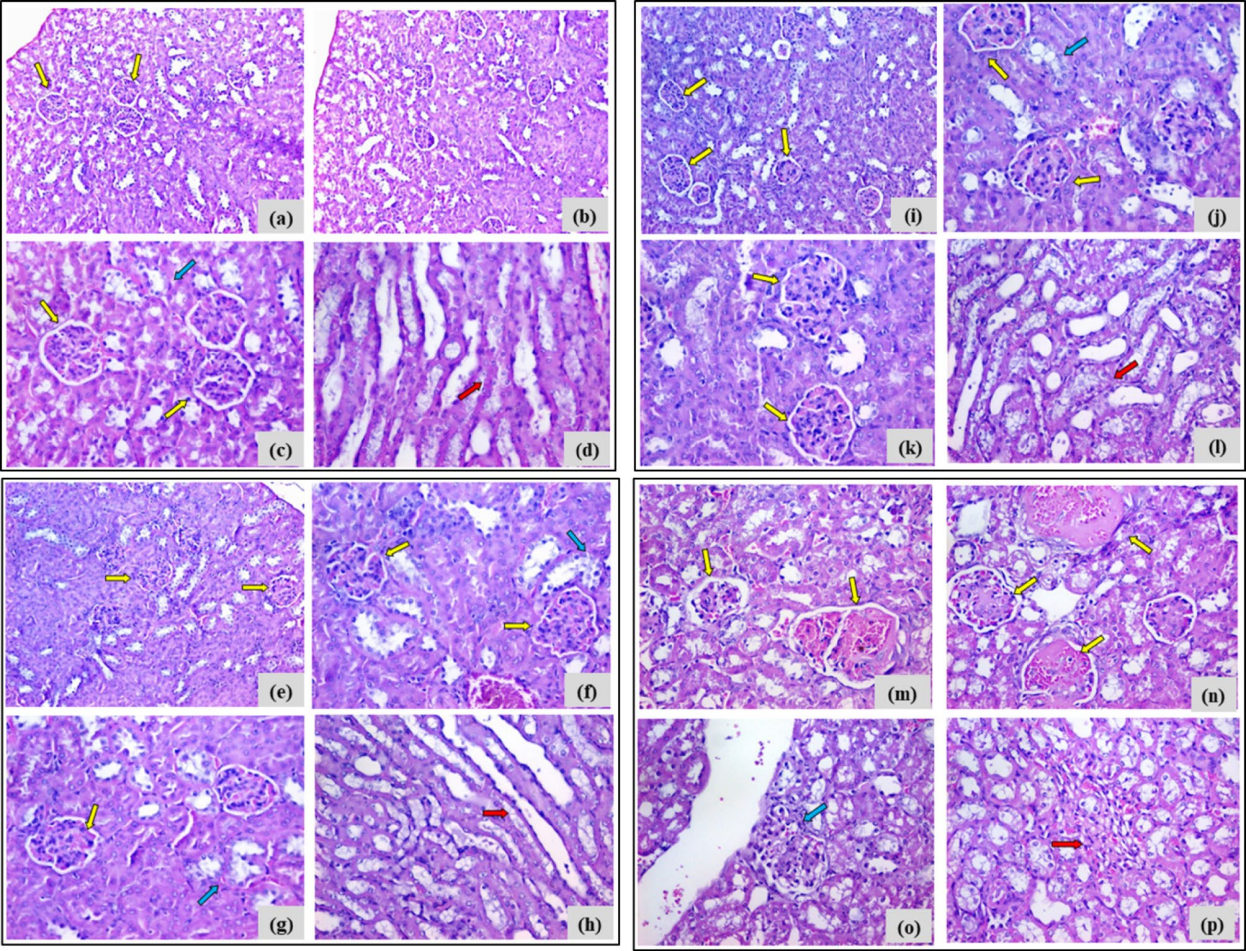
The histopathological findings of the renal tissues of different experimental groups receiving a single intraperitoneal dose of the synthesized compound 4c (12.5, 25, and 50 mg/kg), as compared to the negative control group. Photomicrographs (**a**, **b**, **c**, and **d**) sections: in the kidney of negative control mice showing average glomeruli in number and size (yellow arrows), with intact proximal (blue arrow) and collecting tubules (red arrow) (H&E stain, upper photos X200, lower photos X400). Photomicrographs (**e**, **f**, **g**, and **h**): Sections in the kidney of 12.5 mg 4c-treated mice showing average number and size of glomeruli (yellow arrows), with mild irregularity in the contour as well as mild focal epithelial degeneration of proximal (blue arrow) and collecting tubules (red arrow). (H&E stain; upper left X200; upper right, lower left and lower right X400). Photomicrographs (**i**, **j**, **k**, and **l**): Sections in the kidney of 25 mg 4c-treated mice showing average number and size of glomeruli (yellow arrows), with mild irregularity in the contour as well as mild focal epithelial degeneration of proximal (blue arrow) and collecting tubules (red arrow). (H&E stain, upper left X200; upper right, lower left and lower right X400) Photomicrographs (**m**, **n**, **o**, and **p**): Sections in the kidney of 50 mg 4c-treated mice showing variable glomerular sizes with focal sclerosis (yellow arrows) with focal cortical inflammation (blue arrow) and medullary fibrosis (red arrow) (H&E stain, X400).

Renal tissues of the negative control group: Showed normal histological structure with intact glomeruli and renal tubules. Renal tissues of the 12.5 mg treated group: Demonstrated mild focal epithelial degeneration and slight irregularity in glomerular contours. Renal tissues of the 25 mg treated group: Exhibited changes similar to the 12.5 mg group, with mild degeneration and irregular contours. Renal tissues of the 50 mg treated group: Showed more severe alterations, including variable glomerular sizes, focal sclerosis, cortical inflammation, and medullary fibrosis (Fig. 9, Table 4).

**Table 4:** Comparative table demonstrating the histopathological changes in the renal tissues of mice administrated a single intraperitoneal dose of the synthesized compound **4c** (12.5, 25, and 50 mg/kg).

Group	Glomeruli	Tubules	Other findings
Negative control	Average size and number	Intact proximal and collecting	None
12.5 mg/kg/one day	Mild irregular contour	Mild focal epithelial degeneration	–
25 mg/kg/one day	Mild irregular contour	Mild focal epithelial degeneration	–
50 mg/kg/one day	Variable size, focal sclerosis	Not specified	Focal cortical inflammation, medullary fibrosis

##### Impact of different concentrations of the compound 4c on serum levels of aspartate aminotransferase (AST) and Kidney-injury molecule 1 (KIM-1)

In order to find out which concentration of **4c** could induce hepatotoxicity and to evaluate the proper performance of the liver, serum AST levels were measured. Concentrations of 12.5, 25, and 50 mg/kg caused a non-significant increase in AST levels by 2.7 for a concentration of 12.5 and a significant increase for the other two concentrations by 10.51 and 22.15%, respectively, as compared to negative control mice (Table 5). On the other hand, KIM-1 was employed to evaluate the degree of the inflammation in the renal tissues, the i.p. administration of different concentrations of 12.5, 25, and 50 mg/kg of the **4c** resulted in a significant elevation of serum KIM-1 levels by 295.33, 653.27, and 1035.55%, respectively, as compared to negative control group (Table 5).

**Table 5:** The biochemical changes in the serum biomarkers of hepatic function and renal inflammation in mice administrated a single intraperitoneal dose of the synthesized compound **4c** (12.5, 25, and 50 mg/kg).

Group	Serum AST (IU/L)	Serum KIM-1 (ng/ml)
Negative control	77.97 ± 0.11 ^a^	0.86 ± 0.01 ^a^
12.5 mg	80.07 ± 0.06 ^b^	3.38 ± 0.00 ^b^
25 mg	86.16 ± 0.26 ^c^	6.45 ± 0.01 ^c^
50 mg	95.23 ± 0.29 ^d^	9.72 ± 0.01 ^d^

Data are presented as mean ± standard error of the mean (SEM). Means with different superscripts (a: d) between groups are significantly different at p ≤ 0.05. Statistical analysis was carried out using one-way ANOVA followed by Duncan’s post-hoc test. AST: aspartate aminotransferase; KIM-1: Kidney-injury molecule 1.

##### Impact of different concentrations of the compound 4c on oxidative stress status of lipid peroxidation and nitric oxide (NO) production in the hepatic and renal tissues

The extent of oxidative injury in the liver and kidney was evaluated by estimating levels of malondialdehyde (MDA), an indicator of lipid peroxidation, and nitric oxide (NO). A statistically significant increment in MDA levels was detected in mice that received an intraperitoneal dose of different concentrations of 25 and 50 mg/kg of **4c** by 17.86, and 35.22% respectively, while the dose of 12.5 mg/kg (0.55%) did not cause a significant change, compared to the negative control mice (Table 8). On the other side, hepatic NO contents were significantly increased in mice receiving different concentrations of 12.5, 25, and 50 mg/kg of **4c** by 80.74, 170.64, and 197.19%, respectively, as compared to the negative control mice (Table 6).

**Table 6:** The oxidative status in the hepatic and renal tissues of mice administrated a single intraperitoneal dose of the synthesized compound **4c** (12.5, 25, and 50 mg/kg).

Group	Hepatic tissues		Renal tissues	
	MDA (nmol/g)	NO (µmol/g)	MDA (nmol/g)	NO (µmol/g)
Negative control	174.64 ± 0.02 ^a^	104.95 ± 0.09 ^a^	64.76 ± 0.08 ^a^	72.78 ± 2.39 ^a^
12.5 mg	175.61 ± 0.03 ^b^	189.69 ± 0.09 ^b^	74.37 ± 2.52 ^b^	66.8 ± 1.14 ^a^
25 mg	205.83 ± 0.00 ^c^	284.05 ± 0.00 ^c^	81.49 ± 1.06 ^c^	95.13 ± 9.67 ^b^
50 mg	236.161 ± 0.05 ^d^	311.91 ± 0.00 ^d^	83.21 ± 0.53 ^c^	109.13 ± 6.75 ^b^

Data are presented as mean ± standard error of the mean (SEM). Means with different superscripts (a: d) between groups are significantly different at *p* ≤ 0.05. Statistical analysis was carried out using one-way ANOVA followed by Duncan’s post-hoc test. MDA: Malondialdehyde, NO: Nitric Oxide.

Regarding the stimulated oxidative stress status in the renal tissues, the renal MDA contents were increased significantly in the treated mice by 14.85, 25.85, and 28.50%, respectively, as compared to negative control mice, for concentrations of 12.5, 25, and 50 mg/kg of **4c**. Similarly, renal NO was significantly increased for concentrations of 25 and 50 mg/kg (30.71, and 49.94% respectively), while a concentration of 12.5 mg/kg of **4c** exhibited a non-significant change, compared to the negative control mice (Table 6).

### In silico study

#### Molecular Docking study

**Fig. 10:**
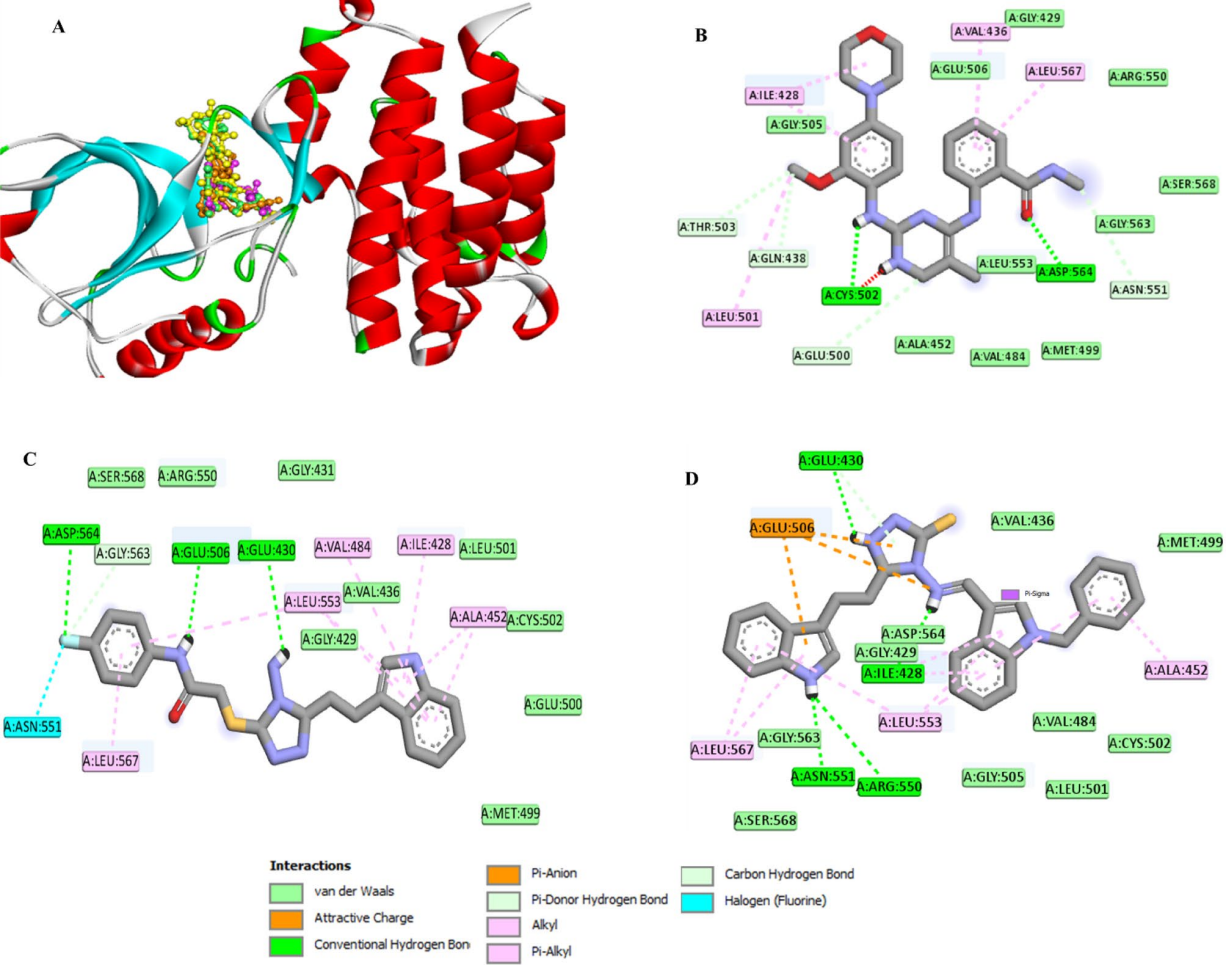
(**A**) The 3D conformations of the co-crystalline ligand, BI9 (yellow), and the re-docked ligand (green), compound 3c (brown), compound 4c (cyan) within the binding pocket of FAK (PDB ID: 2JKK); indicating that they were superimposed in the same position. (**B**) 2D conformations of the re-docked BI9 within the FAK binding pocket (PDB ID: 2JKK). (**C**) 2D conformations of compound 3c within the FAK binding pocket (PDB ID: 2JKK). (**D**) The 2D conformations of the re-docked compound 4c within the FAK binding pocket (PDB ID: 2JKK).

To elucidate the molecular basis of FAK inhibition, docking simulations were conducted for the most active synthesized compounds **3c** and **4c** within the FAK active site (PDB ID: 2JKK). The docking results are summarized in Table 9. The co-crystallized ligand BI9 displayed the best docking score (-10.0 kcal/mol), forming conventional hydrogen bonds with Asp564 and Cys502, in addition to multiple hydrophobic interactions (Table 7, Fig. 10).

**Table 7:** Molecular docking result of the most active synthesized compounds **2**, **3c**, and **4c** inside the active site of Focal Adhesion Kinase (PDB ID: 2JKK).

Compd. No	Score Kcal/mol	Moieties from the compound	Amino acid residues	Type of interaction, Distance Å
Co-crystalline ligand (BI9)	− 10.0	CO, NH	Asp564, Cys502	Conventional H-bond, 2.14, 1.85
		CH_2_, CH_3_	Glu500, Asn551, Gln438, Thr503, Val436, Leu567, Ile428, Leu501	Carbon H-bond, alkyl, π-alkyl
			Glu506, Gly429, Arg550, Ser568, Gly563, Met499, Val484, Ala452, Gly505	Van der Waals
**3c**	− 8.7	NH, NH_2_, F	Glu506, Gly430, Asp564	Conventional H-bond, 2.76, 2.21, 2.16
		F	Gly563, Asn551	Carbon H-bond, Halogen,
		Phenyl, indole moiety	Leu567, Leu553, Val484, Ile428, Ala452	π-alkyl
			Ser568, Arg550, Gly431, Gly429, Val436, Leu501, Vys502, Glu500, Met499	Van der Waals
**4c**	− 9.5	NH_2_, NH,	Glu430, Asn551, Arg550, Ile428	Conventional H-bond, 2.17, 3.03, 2.56, 2.72
		Bis indole moieties, phenyl ring	Glu506, Glu430, Leu567, Leu553, Ala452, Ile428	Attractive charge, π-anion, π-donor H-bon, π-alkyl
			Ser568, Gly563, Gly505, Leu501, Val484, Cys502, Met499, Val436, Asp564, Gly429	Van der Waals

Among the synthesized derivatives, compound **4c** exhibited a high binding affinity (− 9.5 kcal/mol), closely approximating that of BI9. It established several hydrogen bonds with Glu430, Asn551, Arg550, and Ile428, along with attractive charge, π-donor H-bond, π-anion and π-alkyl interactions mediated by its bis-indole and phenyl moieties with key residues, including Glu506, Glu430, Leu567, Leu553, Ala452, and Ile428. These interactions, along with extensive van der Waals contacts, support the formation of a highly stable complex within the ATP-binding cleft of FAK (Fig. 10).

Compound **3c** also showed strong binding affinity (-8.7 kcal/mol), forming three conventional hydrogen bonds with Glu506, Gly430, and Asp564, as well as carbon-hydrogen and halogen bonds involving its fluorine atom and residues Gly563 and Asn551. Its aromatic systems contributed to further stabilization via π-alkyl interactions with Leu567, Leu553, Val484, Ile428 and Ala452 (Fig. 10). Collectively, the docking outcomes correlate well with the ELISA findings, confirming that compound **4c** exhibits the strongest affinity for FAK and achieves the greatest inhibition of FAK protein expression in MDA-MB-231 cells. The presence of bis-indole and amino-triazole-thione functionalities appears to enhance binding through multiple hydrogen bonding and π-π stacking interactions, thereby reinforcing its potential as a promising FAK-targeted anticancer agent.

#### Drug likeliness properties and ADMET prediction

The drug-likeness properties of compounds **3c** and **4c** were evaluated using the *SwissADME* online tool (http://www.swissadme.ch/index.php), and their profiles were compared to the reference drug with doxorubicin. The calculated physicochemical parameters were interpreted according to Lipinski’s Rule of Five and Veber’s criteria, as presented in Table 8^41,42^.

**Table 8:** Physicochemical properties of compounds **3c,** and **4c** using SwissADME online server.

Compd. No	MW g/mol	Log P	HBA	HBD	TPSA Å^2^	Fraction Csp3	MR	nRB	Drug likeness	
									No. Lipinski violation	No. Veber violation
**3c**	410.47	2.77	4	3	126.92	0.15	111.79	8	0	0
**4c**	476.60	4.52	3	1	102.59	0.11	144.07	7	1	0
Doxorubicin	543.52	− 2.1	12	6	206.07	0.44	132.66	5	3	1

MW: Molecular weight; Log P: lipophilicity (log octanol/water partition coefficient); HBA: Hydrogen bond acceptor; HBD: Hydrogen bond donor; TPSA: Topological polar surface area. MR: Molar reactivity, nRB: number of rotatable bonds. Drug likeness (Lipinski Pfizer filter) limits are “Yes, drug-like” for MW ≤ 500, Log p (MLOGP) ≤ 4.15, HBA ≤ 10, and HDD ≤ 5. Veber GSK filter for nRB ≤ 10, TPSA ≤ 140Å^2^.

The results indicated that both compounds generally satisfied Lipinski’s Rule of Five, with compound **4c** displaying a single violation related to its lipophilicity (MLOGP > 4.15). In addition, both compounds complied with Veber’s criteria (nRB ≤ 10 and TPSA ≤ 140 Å^2^), supporting their potential for oral bioavailability. In comparison, the reference drug doxorubicin exhibited multiple violations of both Lipinski’s and Veber’s criteria due to its high molecular weight, elevated TPSA, and excessive numbers of hydrogen bond donors/acceptors.

On the other hand, the ADMET (Absorption, Distribution, Metabolism, Excretion, and Toxicity) properties of compounds **3c** and **4c** were predicted using the *pkCSM* online server (http://biosig.unimelb.edu.au/pkcsm/), and the results were compared with doxorubicin (Table 9).

**Table 9:** Prediction of some of ADMET end points of compounds **3c** and **4c** using the using *pkCSM* server.

Parameters* (unite)	3c	4c	Doxorubicin
Absorption			
Water solubility (log mol/L)	− 4.034	− 5.411	− 3.202
Caco2 permeability (log Papp in 10^−6^ cm/s)	0.327	0.271	0.403
Intestinal absorption (human)	87.39	90.99	62.63
Skin Permeability (log Kp)	− 2.76	− 2.73	− 2.73
P-glycoprotein substrate	Yes	No	Yes
P-glycoprotein I inhibitor	Yes	Yes	No
P-glycoprotein II inhibitor	Yes	Yes	No
Distribution			
VDss (human) (log L/kg)	− 0.182	− 0.403	1.71
Blood–Brain Barrier (log BB)	− 1.223	0.238	− 1.559
CNS permeability (log PS)	− 2.53	− 1.85	− 4.366
Metabolism			
CYP2D6 substrate	No	No	No
CYP3A4 substrate	Yes	Yes	No
CYP1A2 inhibitor	Yes	No	No
CYP2C19 inhibitor	Yes	Yes	No
CYP2C9 inhibitor	Yes	Yes	No
CYP2D6 inhibitor	No	No	No
CYP3A4 inhibitor	Yes	Yes	No
Excretion			
Total Clearance (log ml/min/kg)	− 0.168	0.241	0.817
Renal OCT2 substrate	No	No	No
Toxicity			
Ames mutagenicity	Yes	No	Yes
Max. tolerated dose (human) (log mg/kg/day)	0.5	0.584	0.488
hERG I inhibitor	No	No	No
hERG II inhibitor	Yes	Yes	Yes
Oral Rat Acute Toxicity (LD50, mol/kg)	2.641	3.039	3.693
Oral Rat Chronic Toxicity (log mg/kg_bw/day)	1.874	− 0.235	2.659
Hepatotoxicity	Yes	Yes	No
Skin Sensitization	No	No	No
T.Pyriformis toxicit (log ug/L)	0.314	0.285	0.285
Minnow toxicity (log mM)	− 0.731	− 7.277	7.759

*Solubility is classified by log S values: very soluble (> 0), extremely soluble (− 2 to 0), soluble (− 4 to − 2), moderately soluble (− 6 to − 4), weakly soluble (− 10 to − 6), and insoluble (< − 10). High Caco-2 permeability is indicated by log Papp > 0.9; low skin permeability by log Kp > − 2.5. Volume of distribution (VDss) is low if log VDss < − 0.15 and high if log VDss > 0.45. Compounds with log BB > 0.3 readily cross the blood–brain barrier, while those with log BB < − 1 are poorly distributed to the brain. Central nervous system (CNS) penetration is expected if log PS > − 2, but unlikely if log PS < − 3.

Both compounds showed moderate solubility and high intestinal absorption (87.39% and 90.99%, respectively) suggest good gastrointestinal uptake, markedly higher than doxorubicin (62.63%). Both derivatives exhibited low skin permeability, consistent with limited transdermal diffusion. Notably, 3c was identified as a P-glycoprotein substrate, while **4c** was not, implying that **4c** may have a lower tendency for efflux-mediated resistance. In addition, both compounds were predicted to inhibit P-gp I and II, unlike doxorubicin. The distribution parameters revealed low tissue distribution (log VDss < 0), with **4c** exhibiting moderate blood–brain barrier penetration (log BB = 0.238) and higher CNS permeability than **3c** or doxorubicin. Both derivatives were substrates of CYP3A4, indicating hepatic metabolism via this isoform, and acted as inhibitors of several CYP enzymes, suggesting possible drug–drug interactions during co-administration. The predicted total clearance values revealed that **4c** may be more efficiently cleared than **3c**, although both were lower than doxorubicin.

Toxicity predictions showed 3c mutagenic (Ames test) while **4c** non-mutagenic (like doxorubicin); both hepatotoxic but non-sensitizing, hERG II positive (like doxorubicin), indicating cardiotoxicity risk, with 4c having a higher LD₅₀ than **3c**.

Compound **4c** potent FAK suppression-rivalling GSK-2256098, follows a temporal cascade as predicted by molecular docking: compound **4c** binds FAK’s ATP-binding pocket, forming hydrogen bonds with hinge residues Met499/Glu500/Glu506, confirming immediate kinase inhibition that prevents Y397 autophosphorylation. Inactive FAK cannot recruit Src, disrupting the FAK-Src complex and downstream PI3K/Akt and MAPK/ERK survival pathways^43^. Kinase-dead FAK loses conformational stability and undergoes ubiquitin-proteasomal degradation after 12h, consistent with ATP-competitive inhibitors like GSK2256098^44^. This PI3K/Akt inhibition triggers FoxO3a dephosphorylation and nuclear translocation^45^, contributing to secondary transcriptional repression of PTK2 mRNA. This temporal cascade—kinase inhibition → signaling disruption → protein degradation → mRNA reduction—explain **4c’s** dual suppression of FAK expression at both protein and gene levels.

## Conclusion

In summary, a new series of indole and bis-indole-linked 1,2,4-triazole derivatives was successfully synthesized and characterized, leading to the identification of promising anticancer candidates. Among the synthesized compounds, **4c** exhibited the most potent cytotoxic activity against MCF-7 and MDA-MB-231 breast cancer cells, with significant selectivity toward cancer cells over normal fibroblasts. Mechanistic investigations revealed that **4c** induced S-phase arrest in MCF-7 cells and G1-phase arrest in MDA-MB-231 cells. The striking 4c-mediated effect was apoptosis induction, specifically in MDA-MB-231 cells, approaching almost 90% of the treated population. Furthermore, gene expression studies demonstrated that **4c** markedly downregulated *PTK2* (FAK), *CCL5*, and *BCL2*, while upregulating *CASP3*, highlighting its role as a dual FAK inhibitor and apoptosis inducer. Moreover, **4c** significantly suppressed FAK expression, indicating a FAK-targeted mode of action consistent with its molecular docking profile.

In vivo toxicity study demonstrated that **4c** is well-tolerated at doses up to **12.5 mg/kg,** without detectable hepatotoxicity or nephrotoxicity, this was based on the biochemical and histopathological findings that indicated that the lowest concentration of the compound **4c** (12.5 mg/kg/on day) exhibited the safest toxological profile and could be regarded as a promising candidate as a chemotherapeutic agent. Histopathologically, the study evaluated structural changes in liver and kidney tissues across the three experimental groups, as compared to the negative control group, and the results demonstrated a dose-dependent toxic pattern of tissue alteration in both organs. Mild changes were noted at lower doses (12.5 and 25 mg), while severe tissue damage, including necrosis and fibrosis, was observed at the highest dose (50 mg). These findings suggest potential toxicity risks associated with increasing dosage, underlining the need for cautious dose selection in therapeutic or experimental applications.

Furthermore, the current study demonstrated that the lowest concentration (12.5 mg/kg/on day) was capable of inducing the lowest extent of oxidative stress in the liver and the kidney, in addition, the lowest percentage of change as compared to negative control was recorded for this concentration for both serum levels of AST and KIM-1, as biomarkers related to hepatic dysfunction and renal inflammation. Finally, the histopathological findings of the hepatic and renal tissues run in agreement with the biochemical findings, indicating the lowest toxicity potential of the lowest dose of the synthesized compound **4c**.

Therefore, further experimental studies and safety testing using different doses up to 12.5 mg/kg are required. In addition, further validation in cancer-induced animal models and comparing its therapeutic activities to standard chemodrugs such as doxorubicin or cisplatin is essential to confirm the therapeutic anti-cancer potential and safety of the synthesized compound **4c**.

On the other hand, in silico ADMET predictions supported its favorable pharmacokinetic and safety characteristics. Collectively, these findings highlight compound **4c** as a promising FAK-modulating bis-indole 1,2,4-triazole scaffold with potent anticancer activity, mechanistic selectivity, and an acceptable safety profile, warranting further preclinical optimization and mechanistic exploration as a lead candidate for TNBC therapy.

## Experimental

### Chemistry

#### Materials and reagent

All reagents and solvents were of commercial grade. Indole-3-propionic acid was purchased from Sigma-Aldrich ChemieGmeH, Taufkirchen, Germany. Melting points were determined on the digital melting point apparatus (Electrothermal 9100, Electrothermal Engineering Ltd., serial No. 8694, Rochford, United Kingdom) and are uncorrected. The reaction progress was monitored by thin-layer chromatography (TLC) using silica gel plates (POLYGRAM SILG/UV254, 0.20 mm), which were visualized under UV light 254 and 365 nm. The Fourier Transform Infrared (ATR-FTIR) spectra were obtained employing an FTIR spectrometer (Vertex 70, Bruker); the spectra are determined within a spectral range of 4000–400 cm^−1^ with a spectral resolution of 4 cm^−1^. The ^1^H and ^13^C NMR spectra were recorded using a JEOL-ECA-50 NMR instrument at 500 and 125 MHz, respectively, using TMS as the internal standard, National Research Center, Egypt. Hydrogen coupling patterns are described as (s) singlet, (d) doublet, (t) triplet, (q) quartered, (m) multiple. Chemical shifts were defined as parts per million (ppm) relative to the solvent peak. El-MSElectron ionization mass spectrometry 70 electron volt. Isq thermo scientific Italy 2009. 5-(2-(1*H*-indol-3-yl)ethyl)-4-amino-4*H*-1,2,4- triazole-3-thiol (**2)** was prepared according to the reported methods^46^.

#### General procedure for the preparation of 5-(2-(1*H*-indol-3-yl)ethyl)-4-amino-4*H*-1,2,4-triazole-3-thiol (2)

A mixture of 3-(1*H*-indol-3-yl)propanoic acid (**1,** 0.01mol) and thiocarbohydrazide (0.01 mol) was ground and mixed in a mortar and then heated in an oil bath at 130 °C for 5h. After cooling, a dilute aqueous solution of Na_2_CO_3_ (10 mL, 20%) was added, and the mixture was heated at 70 °C for 30 min. The precipitate formed upon cooling was collected by filtration, washed with water, air-dried, and crystallized from methanol.

#### General procedure for the preparation of 2-((5-(2-(1*H*-Indol-3-yl)ethyl)-4-amino-4*H*-1,2,4-triazol-3-yl)thio)-*N*- acetamide derivatives (3)

A mixture of 5-(2-(1*H*-indol-3-yl)ethyl)-4-amino-4*H*-1,2,4-triazole-3-thiol (**2,** 0.01mol) and 2-chloro acetamide derivatives (0.01mol) in dry acetone (20 mL) containing anhydrous potassium carbonate (0.02 mol), was heated under reflux for 14–16 h (monitored by TLC). The reaction mixture was cooled to room temperature and poured into ice water. The Precipitate formed was filtered off, washed with water, air-dried, and re-crystallized from n-hexane/ethyl acetate.

**2-((5-(2-(1** ***H*** **-indol-3-yl)ethyl)-4-amino-4** ***H*** **-1,2,4-triazol-3-yl)thio)-** ***N*** **-phenylacetamide (3a)**

Gray ppt, m.p*.*112-4 °C; yield: 56% for C_20_H_20_N_6_OS (M.wt 392); IR (cm^−1^) 3414, 3322 (NH_2_), 3233 (NH), 1679 (C=O), 1612 (C=N), 1583 (C=C); ^1^H NMR (500 MHz, DMSO-*d*6) δ 10.77, 10.31 (s, 2H, 2NH), 7.54–6.95 (m, 10H, Ar–H), 5.93 (s, 2H, NH_2_), 4.06 (s, 2H, S-CH_2_), 3.05 (m, 4H, 2CH_2_); ^13^C NMR (125 MHz, DMSO-*d*6) δ 166.71 (C=O), 156.78 (C=N of triazole), 151.43 (C=N of triazole), 139.35, 151.43, 139.35, 136.81, 129.33, 127.50, 124.06, 122.97, 121.50, 119.70, 118.79, 111.93, 39.9 (S-CH_2_), 25.48 (CH_2_), 22.81 (CH_2_).

**2-((5-(2-(1** ***H*** **-indol-3-yl)ethyl)-4-amino-4** ***H*** **-1,2,4-triazol-3-yl)thio)-** ***N*** **-(4-chlorophenyl) acetamide (3b)**

Off white ppt, m.p*.* 111–3 °C; yield: 56%; IR (cm^−1^) 3296 (NH_2_), 3055 (NH), 1668 (C=O), 1630 (C=N), 1592 (C=C);^1^H NMR (500 MHz, DMSO-*d*6) δ 10.77, 10.05 (s, 2H, 2NH), 7.92–6.94 (m, 9H, Ar–H), 5.93 (s, 2H, NH_2_), 4.17 (s, 2H, S-CH_2_), 3.06, 3.02 (m, 4H, 2CH_2_); ^13^C NMR (125 MHz, DMSO-D6) δ 167.88 (C=O), 161.60 (C=N of triazole), 159.83 (C=N of triazole), 156.18, 154.52, 151.38, 136.70, 134.36, 128.08, 127.45, 126.14, 123.16, 121.34, 118.64, 113.75, 111.74, 35.20 (S-CH_2_), 25.45 (CH_2_), 22.88 (CH_2_); m/z: 426/428 (M^+^/M^+^ + 2) for C_20_H_19_ClN_6_OS (M.wt 426.92).

**2-((5-(2-(1** ***H*** **-Indol-3-yl)ethyl)-4-amino-4** ***H*** **-1,2,4-triazol-3-yl)thio)-** ***N*** **-(4-fluorophenyl) acetamide (3c)**

Brown ppt**,** m.p*.* 104-6 °C; yield: 56% for C_20_H_19_FN_6_OS (M.wt 410.47); IR (cm^−1^) 3282 (NH_2_), 3202 (NH), 1656 (C=O), 1605 (C=N), 1546 (C=C) ;^1^H NMR (500 MHz, DMSO-*d*6) δ 10.82, 10.51 (2s, 2H, 2NH), 7.58–7.03 (m, 9H, Ar–H), 5.97 (s, 2H, NH_2_), 4.05 (s, 2H, S-CH_2_), 3.05, 3.02 (m, 4H, 2CH_2_); ^13^C NMR (125 MHz, DMSO-*d*6) δ 184.93 (C=O), 169.23, 166.73, 166.11, 159.63, 157.70, 156.87, 152.30, 151.19, 144.74, 136.85, 135.83, 135.15, 127.57, 122.98, 121.49, 118.77, 115.90, 115.73, 111.94, 56.60 (S-CH_2_), 25.58 (CH_2_), 24.49 (CH_2_).

**2-((5-(2-(1** ***H*** **-Indol-3-yl)ethyl)-4-amino-4** ***H*** **-1,2,4-triazol-3-yl)thio)-** ***N*** **-(4-(** ***N*** **-(pyridin-2-yl) sulfamoyl)phenyl)acetamide (3d)**

Pale brown ppt**,** m.p*.* 79–81 °C; yield: %; IR (cm^−1^) 3353 (NH_2_), 3186, 3052 (NH), 1659 (C=O), 1630 (C=N), 1591 (C=C) ;^1^H NMR (500 MHz, DMSO-*d*6) δ 10.78, 10.59, 10.37 (3s, 3H, 3NH), 7.94–6.67 (m, 13H, Ar–H), 5.91 (s, 2H, NH_2_), 4.06 (s, 2H, S-CH_2_), 3.04–2.95 (m, 4H, 2CH_2_); ^13^C NMR (125 MHz, DMSO-*d*6) δ 175.36 (C=O), 164.72, 163.09, 162.77, 160.94, 151.83, 146.04, 145.30, 134.90, 134.65, 127.68, 123.30, 122.91, 122.83, 116.60, 111.54, 111.38, 101.01, 54.12 (S-CH_2_), 26.46 (CH_2_), 22.79 (CH_2_); m/z: 548 (M^+^) for C_25_H_24_N_8_O_3_S_2_ (M.wt 548.64).

#### General procedure for the preparation of 5-(2-(*N-*substituted-indol-3-yl)ethyl)-4-(((1*H*-indol-3-yl)methylene)amino)-4*H*-1,2,4-triazole-3-thiol (4)

A mixture of 5-(2-(1*H*-indol-3-yl)ethyl)-4-amino-4*H*-1,2,4-triazole-3-thiol (**2**, 0.01mol) and *N*-substituted indole-3-carboxaldehydes (0.01mol) in absolute ethanol (10 mL) containing a few drops of glacial acetic acid was refluxed for 10-12h. After all, starting compounds was consumed as indicated by TLC (n-hexane/ethyl acetate 3:1), the reaction mixture was cooled to room temperature and poured onto ice water. The Precipitate formed was filtered off, washed with water, air-dried, and re-crystallized from n-hexane–ethyl acetate.

**5-(2-(1** ***H*** **-Indol-3-yl)ethyl)-4-(((1** ***H*** **-indol-3-yl)methylene)amino)-4** ***H*** **-1,2,4-triazole-3-thiol (4a)**

Canary ppt**,** m.p*.* 214-6 °C; yield: 95%; IR (cm^−1^) 3278, 3193 (NH), 1631 (C=N), 1591 (C=C), 1241 (C=S); ^1^H NMR (500 MHz, DMSO-*d*6) δ 13.61 (s, 1H, SH), 12.03, 10.78 (2s, 2H, 2NH), 9.50 (s, 1H, CH=N), 8.17 (d, 1H, indolyl H-4^`^), 8.10 (s, 1H, indolyl H-2`), 7.49, 7.43 (2d, 2H, indolyl H-4 & H-7`), 7.26–7.11 (m, 6H, Ar–H), 3.07 (m, 4H, 2CH_2_); ^13^C NMR(125 MHz, DMSO-*d*6) δ 162.55 (S-C=N of triazole), 161.90 (C=N), 151.21 (C=N of triazole), 137.97, 136.78, 135.90, 123.82, 123.10, 122.68, 122.57, 122.12, 121.50, 121.50, 118.80, 111.96, 26.69 (CH_2_), 22.46 (CH_2_); m/z: 386 (M^+^) for C_21_H_18_N_6_S (M.wt 386.48).

**5-(2-(1** ***H*** **-indol-3-yl)ethyl)-4-(((1-ethyl-1** ***H*** **-indol-3-yl)methylene)amino)-4** ***H*** **-1,2,4-triazole-3-thiol (4b)**

Green ppt**,** m.p*.* 234–6 °C; yield: 81%; IR (cm^−1^) 3266 (NH), 1630 (C=N), 1593 (C=C), 1240 (C=S); ^1^H NMR (500 MHz, DMSO-*d*6) δ 13.62 (s, 1H, SH), 10.78 (s, 1H, NH), 9.50 (s, 1H, CH=N), 8.88 (m, 2H, indolyl H-2` & H-4`), 8.42–7.11 (m, 8H, Ar–H), 4.23 (q, 2H, CH_2_), 3.07, 3.06 (m, 4H, 2CH_2_), 1.38 (t, 3H, CH_3_); ^13^C NMR(125 MHz, DMSO-*d*6) δ 162.55 (S-C=N of triazole), 161.85 (C=N), 151.16 (C=N of triazole), 137.69, 137.57, 136.91, 123.08, 122.78, 121.47, 118.75, 118.64, 113.31, 111.92, 111.38, 50.69 (N-CH_2_), 26.65 (CH_2_), 22.43 (CH_2_), 15.69 (CH_3_); m/z: 414 (M^+^) for C_23_H_22_N_6_S (M.wt 414.53).

**5-(2-(1** ***H*** **-indol-3-yl)ethyl)-4-(((1-benzyl-1** ***H*** **-indol-3-yl)methylene)amino)-4** ***H*** **-1,2,4-triazole-3-thiol (4c)**

Brown ppt**,** m.p*.* 176–8 °C; yield: 85%; IR (cm^−1^) 3216 (NH), 2767 (SH), 1617 (C=N), 1593 (C=C);^1^H NMR (500 MHz, DMSO-*d*6) δ 13.63 (s, 1H, SH), 10.78 (s, 1H, NH), 9.53 (s, 1H, CH=N), 8.26, 8.19 (m, 2H, indolyl H-2` & H-4`), 7.30–7.11 (m, 13H, Ar–H), 5.50 (s, 2H, N-CH_2_), 3.08, 3.07 (m, 4H, 2CH_2_); ^13^C NMR(125 MHz, DMSO-*d*6) δ 185.25 (C=S), 171.38 (C=N), 161.91 (C=N), 151.23 (C=N), 137.99, 137.53, 136.79, 135.90, 129.26, 127.84, 123.11, 121.52, 118.82, 113.37, 112.97, 111.97, 60.32 (N-CH_2_), 26.96 (CH_2_), 22.47 (CH_2_); m/z: 476 (M^+^) for C_28_H_24_N_6_S (M.wt 476.60).

#### General procedure for the preparation of 2-((5-(2-(1*H*-indol-3-yl)ethyl)-4-(((1*H*-indol-3-yl)methylene)amino)-4*H*-1,2,4-triazol-3-yl)thio)-*N*-acetamide derivatives (5a-d)

A mixture of Schiff’s base (**4a,** 0.01mol) and 2-chloro acetamide derivatives (0.01mol) in dry acetone (20 mL) containing anhydrous potassium carbonate (0.02 mol), was heated under reflux for 14–16 h (monitored by TLC). The reaction mixture was cooled to room temperature and poured into ice water. The Precipitate formed was filtered off, washed with water, air-dried, and re-crystallized from n-hexane/ethyl acetate.

***2-((5-(2-(1H-indol-3-yl)ethyl)-4-(((1H-indol-3-yl)methylene)amino)-4H-1,2,4-triazol-3-yl)thio)-N-phenyl-acetamide (5a)***

Brown ppt**,** m.p*.* 125–7 °C; yield: %; IR (cm^−1^) 3328, 3227, 3148 (NH), 1675 (C=O), 1612 (C=N), 1580 (C=C); ^1^H NMR (500 MHz, DMSO-*d*6) δ 11.15, 10.81, 10.44 (s, 3H, 3NH), 8.66 (s, 1H, CH=N), 8.20–6.96 (m, 15H, Ar–H), 4.95 (s, 2H, CH_2_), 3.07–2.99 (m, 4H. CH_2_-CH_2_); ^13^C NMR (125 MHz, DMSO-*d*6) δ 176.51 (C=O), 155.77 (S-C=N of triazole), 154.40 (C=N), 142.15 (C=N of triazole), 138.94, 136.81, 132.55, 129.64, 127.75, 126.68, 124.54, 124.30, 122.70, 122.18, 119.74, 118.86, 113.58, 112.50, 11.97, 110.91, 110.15, 53.85 (S-CH_2_), 25.04 (CH_2_), 22.91 (CH_2_); m/z: 519 (M^+^) for C_29_H_25_N_7_OS (M.wt 519.63).

***2-((5-(2-(1H-indol-3-yl)ethyl)-4-(((1H-indol-3-yl)methylene)amino)-4H-1,2,4-triazol-3-yl)thio)-N-(4-chloro-phenyl)acetamide (5b)***

Green ppt**,** m.p*.* 89-91 °C; yield: %; IR (cm^−1^) 3260, 3225, 3165 (NH), 1685 (C=O), 1630 (C=N), 1591 (C=C); ;^1^H NMR (500 MHz, DMSO-*d*6) δ 12.08, 11.51, 10.75 (s, 3H, 3NH), 8.64 (s, 1H, CH=N), 8.15 (d, 1H, indolyl H-4`), 8.00 (s, 1H, indolyl H-2`), 7.81–6.83 (m, 12H, Ar–H), 4.15 (s, 2H, CH_2_), 3.08–2.99 (m, 4H, CH_2_-CH_2_); ^13^C NMR (125 MHz, DMSO-*d*6) δ 168.75 (C=O), 154.39 (C=N of triazole), 141.46 (C=N), 129.54, 128.09, 122.81, 121.47, 119.62, 118.81, 118.74, 113.85, 112.16, 111.87, 54.20 (S-CH_2_), 25.45 (CH_2_), 23.14 (CH_2_); m/z: 588/590 (M^+^/M^+^ + 2) for C_29_H_23_Cl_2_N_7_OS (M.wt 588.51).

***2-((5-(2-(1H-indol-3-yl)ethyl)-4-(((1H-indol-3-yl)methylene)amino)-4H-1,2,4-triazol-3-yl)thio)-N-(4-fluoro-phenyl)acetamide (5c)***

Ppt**,** m.p*.* °C; yield: %; for C_29_H_24_FN_7_OS (537.62); IR (cm^−1^) 3387, 3226 (NH), 1664 (C=O), 1617 (C=N), 1595 (C=C); ^1^H NMR (500 MHz, DMSO-*d*6) δ 12.02, 10.76 (s, 3H, 3NH), 9.52 (s, 1H, CH=N), 8.18 (d, 1H, indolyl H-4`), 8.08 (s, 1H, indolyl H-2`), 7.50–711 (m, 12H, Ar–H), 4.09 (s, 2H, CH_2_), 3.08–3.03 (m, 4H. CH_2_-CH_2_); ^13^C NMR (125 MHz, DMSO-*d*6) 168.65 (C=O), 154.36 (S-C=N of triazole), 141.61 (C=N), 136.51(C=N), 133.95, 130.90, 129.58, 127.83, 126.54, 123.98, 122.22, 119.67, 118.37, 113.47, 112.47, 110.71, 54.08 (S-CH_2_), 25.22 (CH_2_), 22.89 (CH_2_).

***2-((5-(2-(1H-indol-3-yl)ethyl)-4-(((1H-indol-3-yl)methylene)amino)-4H-1,2,4-triazol-3-yl)thio)-N-(4-methoxyphenyl)acetamide (5d)***

Pale green ppt**,** m.p*.* 75–7 °C; yield: %; for C_30_H_27_N_7_O_2_S (549.65) IR (cm^−1^) 3267, 3187, 3115 (NH), 1680 (C=O), 1631 (C=N), 1591 (C=C); ^1^H NMR (500 MHz, DMSO-*d*6) δ 12.02, 10.76 (s, 3H, 3NH), 9.52 (s, 1H, CH=N), 8.18 (d, 1H, indolyl H-4`), 8.08 (s, 1H, indolyl H-2`), 7.50–6.79 (m, 12H, Ar–H), 4.13 (s, 2H, CH_2_), 3.35 (s, 3H, OCH_3_), 3.08–3.03 (m, 4H. CH_2_-CH_2_); ^13^C NMR (125 MHz, DMSO-*d*6) δ 167.18 (C=O), 156.36 (C=N of triazole), 141.69 (C=N), 137.06, 136.03, 132.21, 127.81, 126.78, 124.21, 122.96, 122.17, 121.42, 119.58, 118.86, 114.49, 113.93, 112.11, 111.89, 110.85, 110.34, 55.84 (OCH_3_), 54.02 (CH_2_), 25.24 (CH_2_), 23.21 (CH_2_).

***2-((5-(2-(1H-indol-3-yl)ethyl)-4-(((1H-indol-3-yl)methylene)amino)-4H-1,2,4-triazol-3-yl)thio)-N-(4-(N-(pyridin-2-yl)sulfamoyl)phenyl)acetamide (5e)***

Brown ppt**,** m.p*.* 180–2 °C; yield: %; IR (cm^−1^) 3260, 3225, 3202, 3182 (NH), 1664 (C=O), 1630 (C=N), 1591 (C=C); ^1^H NMR (500 MHz, DMSO-*d*6) δ 12.78, 10.69 (s, 4H, 4NH), 8.64 (s, 1H, CH=N), 8.12–6.71 (m, 18H, Ar–H), 4.14 (s, 2H, CH_2_), 3.08–3.01 (m, 4H. CH_2_-CH_2_); ^13^CNMR (125 MHz, DMSO-*d*6) δ 166.86 (C=O), 163.82 (S-C=N of triazole), 155.55 (C=N), 153.10 (C=N of pyridine), 145.13 (C=N), 139.63, 137.94, 136.74, 128.20, 122.35, 121.42, 119.12, 118.71, 114.18, 111.91, 56.97 (S-CH_2_), 26.55 (CH_2_), 23.06 (CH_2_); m/z: 675 (M^+^) for C_34_H_29_N_9_O_3_S_2_ (675.79).

### Biological activities

#### Ethical approval

All care and procedures for cell culture experiments and the in vivo studies used in this study were performed in accordance with relevant guidelines and regulations of the Declaration of Helsinki, and in accordance with ARRIVE guidelines. Approval was granted by the Medical Research Ethics Committee (MREC) of the NRC, Egypt (Approval no. 13060130).

#### Cytotoxicity (MTT) assay

The adenocarcinoma breast cancer cell lines MDA-MB-231 and MCF-7 were purchased from the American Type Culture Collection (ATCC, Rockville, MD, USA), and the normal human skin fibroblast (hFB) cell line was obtained from the Banco de Células de Rio de Janeiro (BCRJ, Brazil). The tested cell lines were cultured in RPMI-1640 medium (MCF-7 and hFB cells) or DMEM (MDA-MB-231 cells) containing 10% fetal bovine serum (heat-inactivated), supplemented with 5% antibiotics (penicillin and streptomycin), and incubated in a humidified air chamber with 5% CO_2_. The cell lines were propagated in cell culture flasks until the monolayer reached 85% confluency. Subsequently, the cells were sub-cultured in 96-well plates at a density of 5,000 cells/ well and treated with the synthesized compounds at variable concentrations for different time intervals (24 h and 48 h). The cytotoxic activity was estimated using the 3-(4,5-dimethyl-2-thiazolyl)-2,5-diphenyl-2H-tetrazolium bromide (MTT) assay as described by Hansen et al.^47^.

Briefly, after the treatment period, 20 µL of MTT solution (5 mg/mL in PBS) was added to each well and incubated for 4 h at 37 °C. The medium was then carefully removed, and the resulting insoluble formazan crystals were dissolved in 100 µL of DMSO, and the absorbance was measured at 570 nm using a microplate reader. The data were expressed as the mean percentage of viable cells as compared to the respective control cultures treated with the solvent from three independent experiments (n = 3). The half maximal growth inhibitory concentration IC_50_ values were calculated from the line equation of the dose–dependent curve of each compound.

#### Evaluation of cell migration, chemotaxis, and invasion capacity using the cell migration assay

In this protocol, we used the standard methodology for wound healing assay using live-cell microscopy as described by^48^.

##### Sample preparation: creating the gap

A cell-free gap was created in a cell monolayer by physical exclusion, as a “cell exclusion” collective cell migration assay, to simulate wounding and to create a gap by scratching a confluent monolayer with a pipette tip. The cultured plates were washed twice with phosphate buffer saline to remove debris from damaged or dead cells, particularly after physical scratching. Then they were incubated with complete media containing the tested chemicals for different time intervals 24, and 48h. The cultured plates were rinsed twice with phosphate-buffered saline, and image acquisition is started at each related time point.

##### Image processing and analysis

Once the digital images were recorded, the gap size was measured as a function of time using Olympus microscopy with a digital camera (PowerShot A20, Canon, USA).

Having measured the gap area for each frame in the wound healing experiment, we plotted the gap area as a function of time to derive the cell migration rate v _migration_ and also the t1/2gap value according to^48^.

In addition, by plotting the gap area versus time, the cell migration rate was readily extrapolated from the data well before the gap had fully closed in, and the actual cell migration rate was calculated according to real-world units of µm^2^/hr.

#### Cell cycle analysis

Cell cycle analysis was performed according to Nunez ^49^. MDA-MB-231 (TNBC) and MCF-7 (non-TNBC) cells were cultured in DMEM HG or RPMI, respectively, supplemented with 100 U/mL penicillin, 100 mg/mL streptomycin, and 10% heat-inactivated FBS at 37 °C in a humidified atmosphere containing 5% CO₂. After 48h treatment with test compounds, 1 × 10^5^ cells were trypsinized, washed twice with ice-cold PBS (pH 7.4), fixed in 60% ice-cold ethanol (4 °C, 1h), then stained with RNase A (50 µg/mL) and propidium iodide (10 µg/mL) for 20 min at 37 °C in the dark. DNA content was analyzed by flow cytometry (FL2 channel, λex/em 535/617 nm; ACEA Novocyte™, ACEA Biosciences), acquiring 12,000 events/sample, with distribution calculated using NovoExpress™ software. All experiments were performed in three independent biological replicates (n = 3), each derived from separate cell cultures and conducted on different days. One sample per treatment condition was analyzed within each biological replicate.

#### Apoptosis and necrosis analysis

After treatment with the TNBC and non-TNBC with the test compounds for 48h, apoptosis and necrosis cell populations are determined using the Annexin V-FITC apoptosis detection kit (Abcam Inc., Cambridge Science Park, Cambridge, UK) according to the manufacturer’s instructions. Briefly, 1 × 10^5^ cells were collected by trypsinization and washed twice with ice-cold PBS (pH 7.4). Then, cells were incubated in the dark with 0.5 ml of Annexin V-FITC/PI solution for 30 min at room temperature. After staining, cells are injected via ACEA Novocyte™ flowcytometer (ACEA Biosciences Inc., San Diego, CA, USA) and analyzed for FITC and PI fluorescent signals using FL1 and FL2 signal detectors, respectively (λex/em 488/530 nm for FITC and λex/em 535/617 nm for PI). A minimum of 12,000 events were acquired per sample.

Initial gating was performed on forward scatter (FSC) versus side scatter (SSC) plots to exclude cellular debris and aggregates. Doublet discrimination was applied using FSC-area versus FSC-height parameters to ensure analysis of single-cell populations only. Quadrant gates were subsequently established on Annexin V-FITC versus PI dot plots using unstained, single-stained, and compensation controls. Cell populations were defined as follows:

- Annexin V − /PI − : viable cells
- Annexin V + /PI − : early apoptotic cells
- Annexin V + /PI + : late apoptosis/secondary necrosis
- Annexin V − /PI + : primary necrotic cells

All apoptosis experiments were conducted in three independent biological replicates (n** = **3) performed on separate days from independently cultured cells.

#### Real-time quantitative PCR for gene expression

Total RNA was isolated from treated and untreated MDA-MB-231 and MCF-7 cell lines using a total RNA purification kit (NORGEN, Canada) following the manufacturer’s instructions. RNA concentration and purity were checked using a NanoDrop 2000 spectrophotometer (Thermo Fisher Scientific, USA). cDNA was reverse-transcribed from 1 μg of extracted RNA using Revert Aid First Strand cDNA synthesis Kit (Thermo Fisher Scientific, Lithuania). Quantitative real time-PCR reaction was performed in an ABI 7500 fast PCR system (Applied Biosystems, USA) using Hera plus SYBR Green qPCR kit (Willow fort, Birmingham, UK). The primers of the endogenous control gene (GAPDH) and the target genes were designed using net primer software (https://www.premierbiosoft.com/netprimer/), NCBI primer blast software (https://www.ncbi.nlm.nih.gov/tools/primer-blast), and UCSC software (https://genome.ucsc.edu/cgi-bin/hgPcr). Designed primers were purchased from willowfort.co.uk. and listed in Table 10. Melting curve analysis was done to ensure the specificity of the amplification product. The ΔCT method was used for gene expression calculations^35,50^. Experiments were performed with three independent biological replicates (n** = **3) per condition for each cell line. Each biological replicate corresponded to an independently seeded and treated cell culture which was processed independently through RNA extraction, cDNA synthesis, and qPCR measurement^51^. Results are presented as mean value ± SEM.

**Table 10:** Primers in this study.

No	RT-qPCR primer Name	Sequences(5’-3’)
1	CDH1-F	TTAGAGGTGGGTGACTACAAAATC
2	CDH1-R	AGCAAGAGCAGCAGAATCAG
3	BCL2-F	ACTTCGCCGAGATGTCCAG
4	BCL2-R	AGTTCCACAAAGGCATCCC
5	CASP 3-F	TACATGGAAGCGAATCAATGG
6	CASP 3-R	TCCTTTTGCTGTGATCTTCTTTAG
7	GAPDH-F	AAGGCTGGGGCTCATTTG
8	GAPDH-R	TGCTGATGATCTTGAGGCTG
9	PDL-F	TGCCGACTACAAGCGAATTACTG
10	PDL-R	CTGCTTGTCCAGATGACTTCGG
11	VEGFR2-F	CGGACAGTGGTATGGTTCTTGC
12	VEGFR2-R	GTGGTGTCTGTGTCATCGGAGTG
13	PTK2-F	AGATCCTGTCTCCAGTCTAC
14	PTK2-R	AATGGTTTGCACTTGAGTGA
15	CCL5-F	ATCCTCATTGCTACTGCCCTC
16	CCL5-R	ACCTGTGGACGACTGCTGG
17	MMP14-F	TGACGGGAACTTTGACACC
18	MMP14-R	TTTGCCATCCTTCCTCTCGT
19	Vim-F	CTCCTCCCCCTGTCACATAC
20	Vim-R	TGATTGGCATCAGGACCGTT

#### ELISA analysis of FAK expression

Focal adhesion kinase (FAK) expression, in the MDA-MB-231 cell line without and after treatment with compounds **2**, **3c**, or **4c**, was quantified using the Human SimpleStep ELISA® Kit (Abcam, Cambridge, UK; catalog no. ab187395) following the manufacturer’s protocol^22^. Briefly, Samples or standard serials were incubated for 1 h with the supplied antibody cocktail at room temperature on a shaker set to 400 rpm. The ELISA plates were then washed extensively to remove unconjugated antibodies. Then, 3, 3’, 5, 5’-Tetramethylbenzidine (TMB) substrate was added for 10 min in the dark, then the reaction was stopped, and absorbance was measured at 450 nm. The average zero standard was subtracted from all readings. Duplicate readings of the positive control dilutions were averaged and plotted against their concentrations. A smooth curve was fitted through these points to construct the standard curve. Protein concentrations of unknown samples were then calculated based on this standard curve.

#### In vivo acute toxicity study

##### Chemicals and reagents

Biochemical analyses of aspartate aminotransferase (AST), malondialdehyde (MDA), and Nitric oxide (NO) were conducted using commercially available colorimetric kits from Biodignostic Chemical Company, Egypt. The ELISA kit for Kidney Injury Molecule-1 (KIM-1) was purchased from CUSABIO (catalog No: CCSB-E08808r).

##### Animal source and care

Albino white mice weighing between 25 and 30 g were sourced from the animal house of the National Research Centre (NRC), Egypt. The animals were housed in the animal house of NRC. The mice were kept in cages and maintained under standard conditions (12:12 h light/dark cycle, controlled room temperature (23 ± 2 °C), stress-free, ad libitum water, standard diets, and sanitary conditions). Before commencing the experiment, the mice were allowed to acclimatize for a period of one week to reduce stress.

##### Acute toxicity study

To evaluate the toxicity of the synthesized compound **4c**, an in vivo toxicity assay was conducted by treating mice with a single intraperitoneal (i.p.) injection of three different concentrations of this compound, and then biochemical and histopathological analyses following treatment were performed. The control group was administered solely with the vehicle, DMSO, while the other three groups of mice received a single i.p. dose of increasing concentrations of **4c** (12.5, 25, and 50 mg/kg/day) dissolved in the vehicle, DMSO.

##### Experimental design

Sixteen albino mice (n = 4) were used and allocated randomly into four different groups:

Group (1): Negative control mice receiving a single intraperitoneal (i.p.) dose of the vehicle, DMSO.

Group (2): (12.5 mg/kg 4c received group) Mice received a single i.p. dose of 12.5 mg/kg of **4c**.

Group (3): (25 mg/kg 4c received group) Mice received a single i.p. dose of 25 mg/kg of compound **4c**.

Group (4): (50 mg/kg 4c received group) Mice received a single i.p. dose of 50 mg/kg of compound **4c**.

The mice were observed for the next 48 h, for morphological features, behavioral alterations, or mortality and other signs of toxicity.

##### Collection and preparation of blood and tissue samples

At the end of the experiment, Thiopental sodium (50 mg/kg, i.p., Thiopental®, Biochemie GmbH, Vienna, Austria) was used to anesthetize mice that were fasted overnight, with free access to water; then blood was collected from the orbital plexus, and the anesthetized mice were sacrificed by cervical dislocation. Blood samples were centrifuged at 3000 rpm and stored at -80 ^°^C for further biochemical examinations. Under deep anaesthesia, the mice were slaughtered and sacrificed by cervical dislocation. Livers and kidneys were rapidly excised, cleaned, dissected, and washed with normal saline. Tissue homogenates were prepared by homogenization of the liver and kidney samples in cold Phosphate-buffered saline (PBS; pH 7.4). Subsequently, homogenates were centrifuged (10,000×*g* for 20 min at 4 °C), and the clear supernatants were quickly collected and stored at -80 °C until use. Liver and kidney samples (n = 3) were specified for biochemical analysis of oxidative stress.

##### Biochemical investigations

Biochemical assays in mice serum and tissue homogenates were carried out on a UV/Vis Spectrophotometer (Aglient Technologies, India). To evaluate hepatic function, serum levels of aminotransferase (AST) was estimated^52^. To evaluate renal function, serum levels of kidney-injury molecule-1 (Kim-1) was measured colorimetrically using the ELISA technique.

##### Evaluation of oxidative stress status in the hepatic and renal tissues

*Lipid peroxidation (LPO)* Lipid peroxidation in the hepatic and renal tissues was estimated colorimetrically, according to Ohkawa et al.^53^. Lipid peroxidation levels were estimated *by* measuring thiobarbituric acid reactive substances (TBARS) as malondialdehyde (MDA), a product of lipid peroxidation. The reaction between thiobarbituric acid and MDA, in acidic medium, resulted in pink colored end-product that was measured spectrophotometrically at 534 nm. The results were expressed as nmol/g of wet tissue.

*Determination of NO content* Nitric oxide (NO) production in the hepatic and renal tissues was determined by estimating the total nitrite level, which is determined by reaction with Griess reagent using sodium nitrite as a standard according to Montgomery and Dymock^54^, spectrophotometrically at 540 nm. The data were expressed as µmol/g of wet tissue.

##### Histopathological investigation

Samples of liver and kidneys were collected from all groups, immediately fixed in 10% neutral buffered formalin, and routinely processed according to Suvarna et al.^55^. Sections of 4-5μm thickness were prepared and stained with Hematoxylin and Eosin (H&E) for histopathological investigation by a light microscope (Olympus BX50, Tokyo, Japan). Signs of organ tissue damage are recorded^56^. The histology of each organ was compared with that of the negative control organ.

##### Statistical analysis

Data are presented mean ± standard error of the mean (SEM) from three independent experiments. Statistical analyses were conducted using GraphPad Prism version 8.0.2 or SPSS version 20. The normality of data distribution was evaluated with the Shapiro–Wilk test. For pairwise comparisons, Welch’s t-test was applied to parametric data, while the Mann–Whitney test was utilized for nonparametric data. Multiple group comparisons were performed using one-way analysis of variance (ANOVA) followed by Tukey’s, or Bonferroni post hoc tests. A p-value less than 0.05 was considered statistically significant.

### In-silico studies

#### Molecular Docking

Molecular docking was done using PyRx version 8 AutoDock Vina^57^. Focal Adhesion Kinase (PDB ID: 2JKK) three-dimensional structures were obtained from the RCSB protein data bank (https://www.rcsb.org/access on 25 September 2025)^58^. The unwanted co-crystallized ligands and water molecules were removed, and the enzymes were prepared using VEGA ZZ, saved as pdb and converted to PDBQT format by Autodock vina tools. The chemical structure of co-crystalline ligand, 2-({5-chloro-2-[(2-methoxy-4-morpholin-4-ylphenyl)amino]pyrimidin-4-yl}amino)-n-methylbenzamide** (BI9)** was obtained from the PubChem database as an SDF file. While the structure of the specified molecules was constructed with ChemDraw ultra-10.0 and saved as an SDF files. After that, all the SDF files were minimized utilizing the MMFF94 force field, then converted to a pdbqt file by OpenBable tools in pyrx software. AutoDock Tools establish the grid box size and center. The FAK active site was centered at x = 8.28, y = 0.137, and z = 26.55, with dimensions of 19.01 × 18.56 × 24.12 Å. The co-crystalline ligand was re-docked into the enzyme active sites to verify our docking protocol. PyRx software presents the 8 most suitable docking poses of the ligand–protein complex after the docking is completed and subsequently ranked according to the binding energy. We have selected the first docking pose, which is the most suitable pose where the ligands have the lowest binding energy, zero root-mean-square deviation (RMSD) and strongly interact with the protein’s catalytic cavity and visualized them using BIOVIA Discovery Studio Visualizer to have a great insight into ligand binding position in the protein cavity.

#### Drug likeness and ADMET prediction

Drug likeness and some ADMET endpoints of the most active compounds, **3c,** and **4c,** were predicted utilizing the SwissADME tool, accessible at http://www.swissadme.ch/index.php and *pkCSM* website (http://biosig.unimelb.edu.au/pkcsm/)^59,60^. Drug likeness analyses were performed on the computed physicochemical descriptors, such as molecular MW, LogP, HBA, HBD, nRB, MR, and TPSA, using Lipinski’s rule of five and the Veber filter rule in consideration^41,42^.

## Supplementary Information

Below is the link to the electronic supplementary material.

Supplementary Material 1

## Data Availability

The IR, NMR and Mass spectra are available in the supporting information file.
