# Supplementary material for: New indole-linked 1,2,4-triazole derivatives as dual FAK inhibitors and apoptosis inducers targeting survival and migration in triple-negative breast cancer in-vitro
Source: Sci Rep. 2026 Apr 22;16:13134. doi: 10.1038/s41598-026-41032-1 (PMC13103447; doi:10.1038/s41598-026-41032-1)
Supplement: Supplementary file 1 — Supplementary Material 1 [file 41598_2026_41032_MOESM1_ESM.pdf]

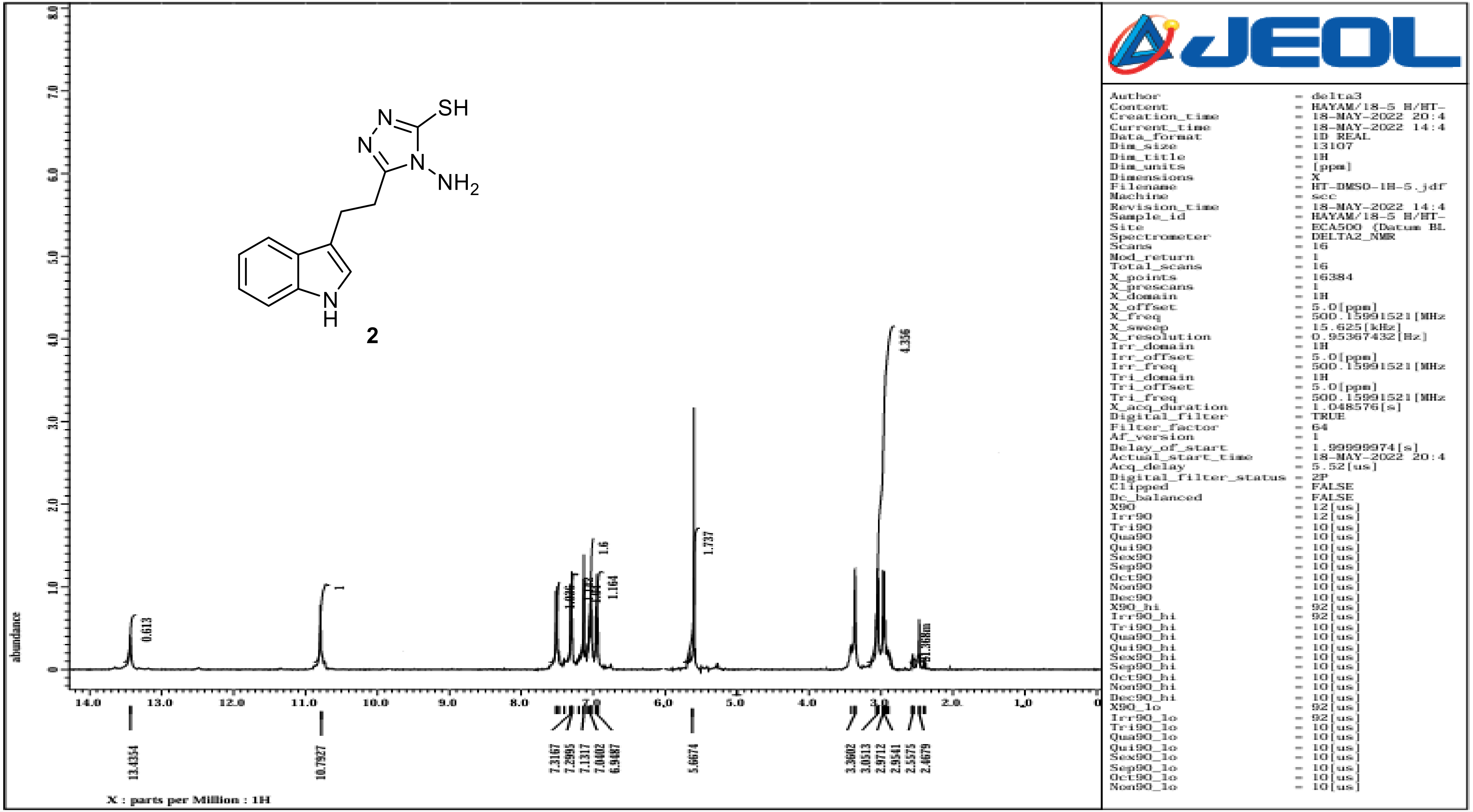
SH
N
N
N
NH2
N
H
2
JEOL
Author = delta3
Content = HAYAM/18-5 H/HT-
Creation_time = 18-MAY-2022 20:4
Current_time = 18-MAY-2022 14:4
Data_format = 1D REAL
Dim_size = 13107
Dim_title = 1H
Dim_units = [ppm]
Dimensions = X
Filename = HT-DMSO-1H-5.jdf
Machine = scc
Revision_time = 18-MAY-2022 14:4
Sample_id = HAYAM/18-5 H/HT-
Site = ECA500 (Datum BL
Spectrometer = DELTA2_NMR
Scans = 16
Mod_return = 1
Total_scans = 16
X_points = 16384
X_prescans = 1
X_domain = 1H
X_offset = 5.0[ppm]
X_freq = 500.15991521[MHz
X_sweep = 15.625[kHz]
X_resolution = 0.95367432[Hz]
Irr_domain = 1H
Irr_offset = 5.0[ppm]
Irr_freq = 500.15991521[MHz
Tri_domain = 1H
Tri_offset = 5.0[ppm]
Tri_freq = 500.15991521[MHz
X_acq_duration = 1.048576[s]
Digital_filter = TRUE
Filter_factor = 64
Af_version = 1
Delay_of_start = 1.99999974[s]
Actual_start_time = 18-MAY-2022 20:4
Acq_delay = 5.52[us]
Digital_filter_status = 2P
Clipped = FALSE
Dc_balanced = FALSE
X90 = 12[us]
Irr90 = 12[us]
Tri90 = 10[us]
Qua90 = 10[us]
Qui90 = 10[us]
Sex90 = 10[us]
Sep90 = 10[us]
Oct90 = 10[us]
Non90 = 10[us]
Dec90 = 10[us]
X90_hi = 92[us]
Irr90_hi = 92[us]
Tri90_hi = 10[us]
Qua90_hi = 10[us]
Qui90_hi = 10[us]
Sex90_hi = 10[us]
Sep90_hi = 10[us]
Oct90_hi = 10[us]
Non90_hi = 10[us]
Dec90_hi = 10[us]
X90_lo = 92[us]
Irr90_lo = 92[us]
Tri90_lo = 10[us]
Qua90_lo = 10[us]
Qui90_lo = 10[us]
Sex90_lo = 10[us]
Sep90_lo = 10[us]
Oct90_lo = 10[us]
Non90_lo = 10[us]
abundance
X : parts per Million : 1H
0.613
1
1.164
1.6
1.737
4.356
13.4354
10.7927
7.3167
7.2995
7.1317
7.0402
6.9487
5.6674
3.3602
3.0513
2.9712
2.9541
2.5575
2.4679

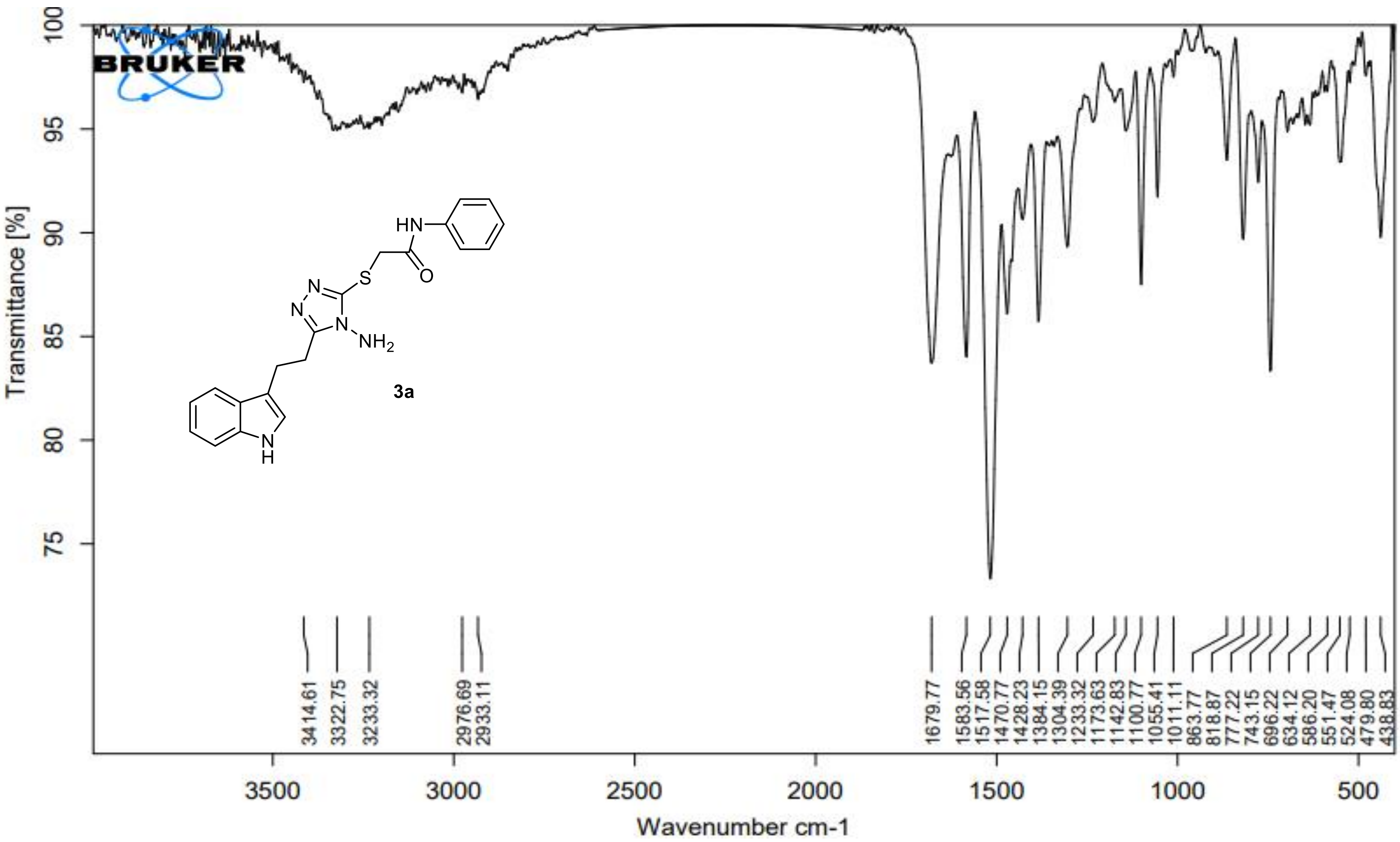
BRUKER
Transmittance [%]
Wavenumber cm-1
100
95
90
85
80
75
3500
3000
2500
2000
1500
1000
500
3414.61
3322.75
3233.32
2976.69
2933.11
1679.77
1583.56
1517.58
1470.77
1428.23
1384.15
1304.39
1233.32
1173.63
1142.83
1100.77
1055.41
1011.11
863.77
818.87
777.22
743.15
696.22
634.12
586.20
551.47
524.08
479.80
438.83
HN
S
O
N
N
N
NH2
N
H
3a

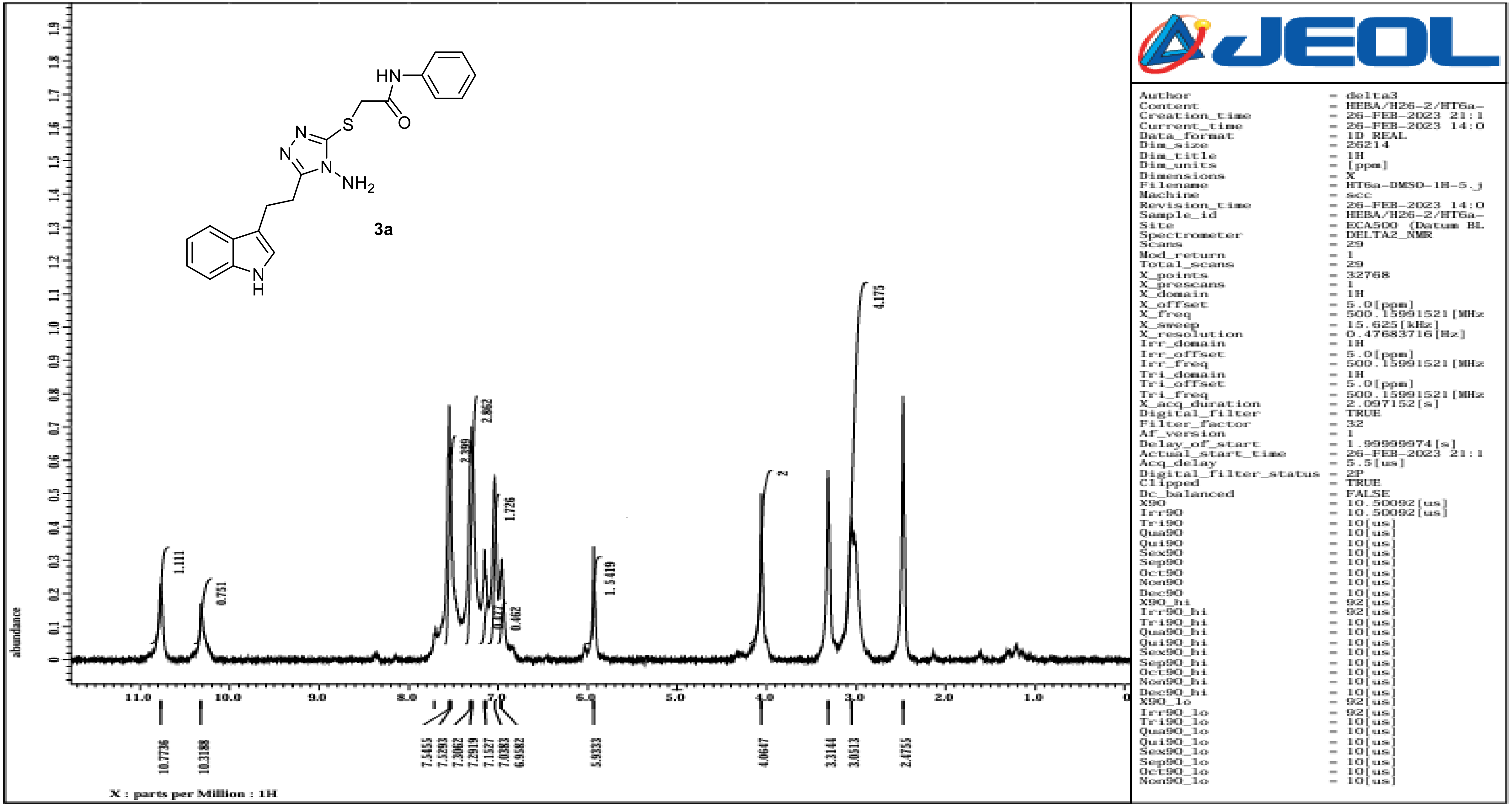

HN
S
O
N
N
N
NH2
N
H
3a
abundance
X : parts per Million : 1H
1.111
0.751
2.399
2.862
1.726
0.462
1.5419
2
4.175
10.7736
10.3188
7.5455
7.5293
7.3062
7.2919
7.1527
7.0383
6.9582
5.9333
4.0647
3.3144
3.0513
2.4755
JEOL
Author = delta3
Content = HEBA/H26-2/HT6a-
Creation_time = 26-FEB-2023 21:1
Current_time = 26-FEB-2023 14:0
Data_format = 1D REAL
Dim_size = 26214
Dim_title = 1H
Dim_units = [ppm]
Dimensions = X
Filename = HT6a-DMSO-1H-5.j
Machine = scc
Revision_time = 26-FEB-2023 14:0
Sample_id = HEBA/H26-2/HT6a-
Site = ECA500 (Datum BL
Spectrometer = DELTA2_NMR
Scans = 29
Mod_return = 1
Total_scans = 29
X_points = 32768
X_prescans = 1
X_domain = 1H
X_offset = 5.0[ppm]
X_freq = 500.15991521[MHz
X_sweep = 15.625[kHz]
X_resolution = 0.47683716[Hz]
Irr_domain = 1H
Irr_offset = 5.0[ppm]
Irr_freq = 500.15991521[MHz
Tri_domain = 1H
Tri_offset = 5.0[ppm]
Tri_freq = 500.15991521[MHz
X_acq_duration = 2.097152[s]
Digital_filter = TRUE
Filter_factor = 32
Af_version = 1
Delay_of_start = 1.99999974[s]
Actual_start_time = 26-FEB-2023 21:1
Acq_delay = 5.5[us]
Digital_filter_status = 2P
Clipped = TRUE
Dc_balanced = FALSE
X90 = 10.50092[us]
Irr90 = 10.50092[us]
Tri90 = 10[us]
Qua90 = 10[us]
Qui90 = 10[us]
Sex90 = 10[us]
Sep90 = 10[us]
Oct90 = 10[us]
Non90 = 10[us]
Dec90 = 10[us]
X90_hi = 92[us]
Irr90_hi = 92[us]
Tri90_hi = 10[us]
Qua90_hi = 10[us]
Qui90_hi = 10[us]
Sex90_hi = 10[us]
Sep90_hi = 10[us]
Oct90_hi = 10[us]
Non90_hi = 10[us]
Dec90_hi = 10[us]
X90_lo = 92[us]
Irr90_lo = 92[us]
Tri90_lo = 10[us]
Qua90_lo = 10[us]
Qui90_lo = 10[us]
Sex90_lo = 10[us]
Sep90_lo = 10[us]
Oct90_lo = 10[us]
Non90_lo = 10[us]

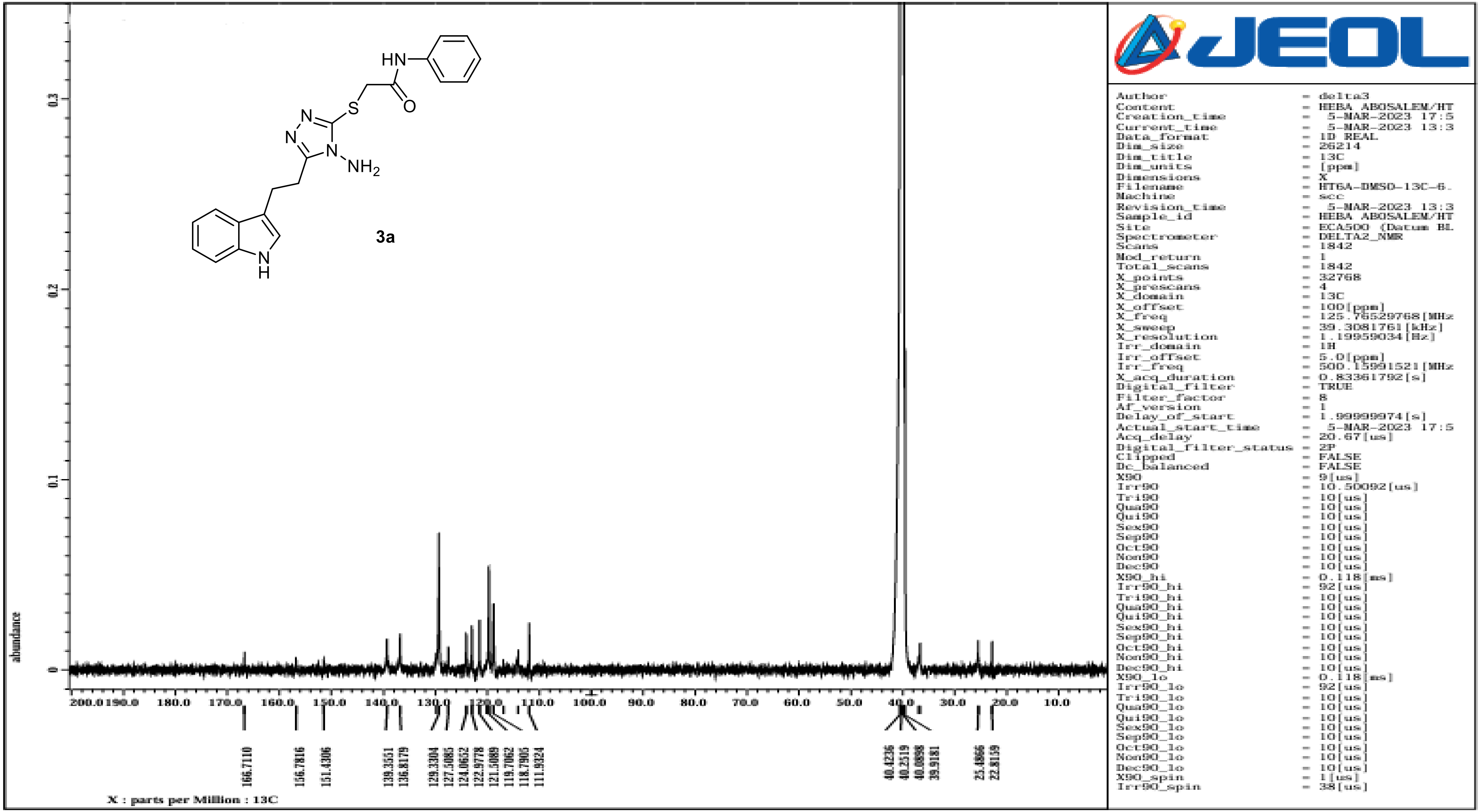
3a
HN
S
O
N
N
N
NH2
N
H
abundance
0.3
0.2
0.1
0
X : parts per Million : 13C
166.7110
156.7816
151.4306
139.3551
136.8179
129.3304
127.5085
124.0652
122.9778
121.5089
119.7062
118.7905
111.9324
40.4236
40.2519
40.0898
39.9181
25.4866
22.8159
JEOL
Author = delta3
Content = HEBA ABOSALEM/HT
Creation_time = 5-MAR-2023 17:5
Current_time = 5-MAR-2023 13:3
Data_format = 1D REAL
Dim_size = 26214
Dim_title = 13C
Dim_units = [ppm]
Dimensions = X
Filename = HT6A-DMSO-13C-6.
Machine = scc
Revision_time = 5-MAR-2023 13:3
Sample_id = HEBA ABOSALEM/HT
Site = ECA500 (Datum BL
Spectrometer = DELTA2_NMR
Scans = 1842
Mod_return = 1
Total_scans = 1842
X_points = 32768
X_prescans = 4
X_domain = 13C
X_offset = 100[ppm]
X_freq = 125.76529768[MHz
X_sweep = 39.3081761[kHz]
X_resolution = 1.19959034[Hz]
Irr_domain = 1H
Irr_offset = 5.0[ppm]
Irr_freq = 500.15991521[MHz
X_acq_duration = 0.83361792[s]
Digital_filter = TRUE
Filter_factor = 8
Af_version = 1
Delay_of_start = 1.99999974[s]
Actual_start_time = 5-MAR-2023 17:5
Acq_delay = 20.67[us]
Digital_filter_status = 2P
Clipped = FALSE
Dc_balanced = FALSE
X90 = 9[us]
Irr90 = 10.50092[us]
Tri90 = 10[us]
Qua90 = 10[us]
Qui90 = 10[us]
Sex90 = 10[us]
Sep90 = 10[us]
Oct90 = 10[us]
Non90 = 10[us]
Dec90 = 10[us]
X90_hi = 0.118[ms]
Irr90_hi = 92[us]
Tri90_hi = 10[us]
Qua90_hi = 10[us]
Qui90_hi = 10[us]
Sex90_hi = 10[us]
Sep90_hi = 10[us]
Oct90_hi = 10[us]
Non90_hi = 10[us]
Dec90_hi = 10[us]
X90_lo = 0.118[ms]
Irr90_lo = 92[us]
Tri90_lo = 10[us]
Qua90_lo = 10[us]
Qui90_lo = 10[us]
Sex90_lo = 10[us]
Sep90_lo = 10[us]
Oct90_lo = 10[us]
Non90_lo = 10[us]
Dec90_lo = 10[us]
X90_spin = 1[us]
Irr90_spin = 38[us]

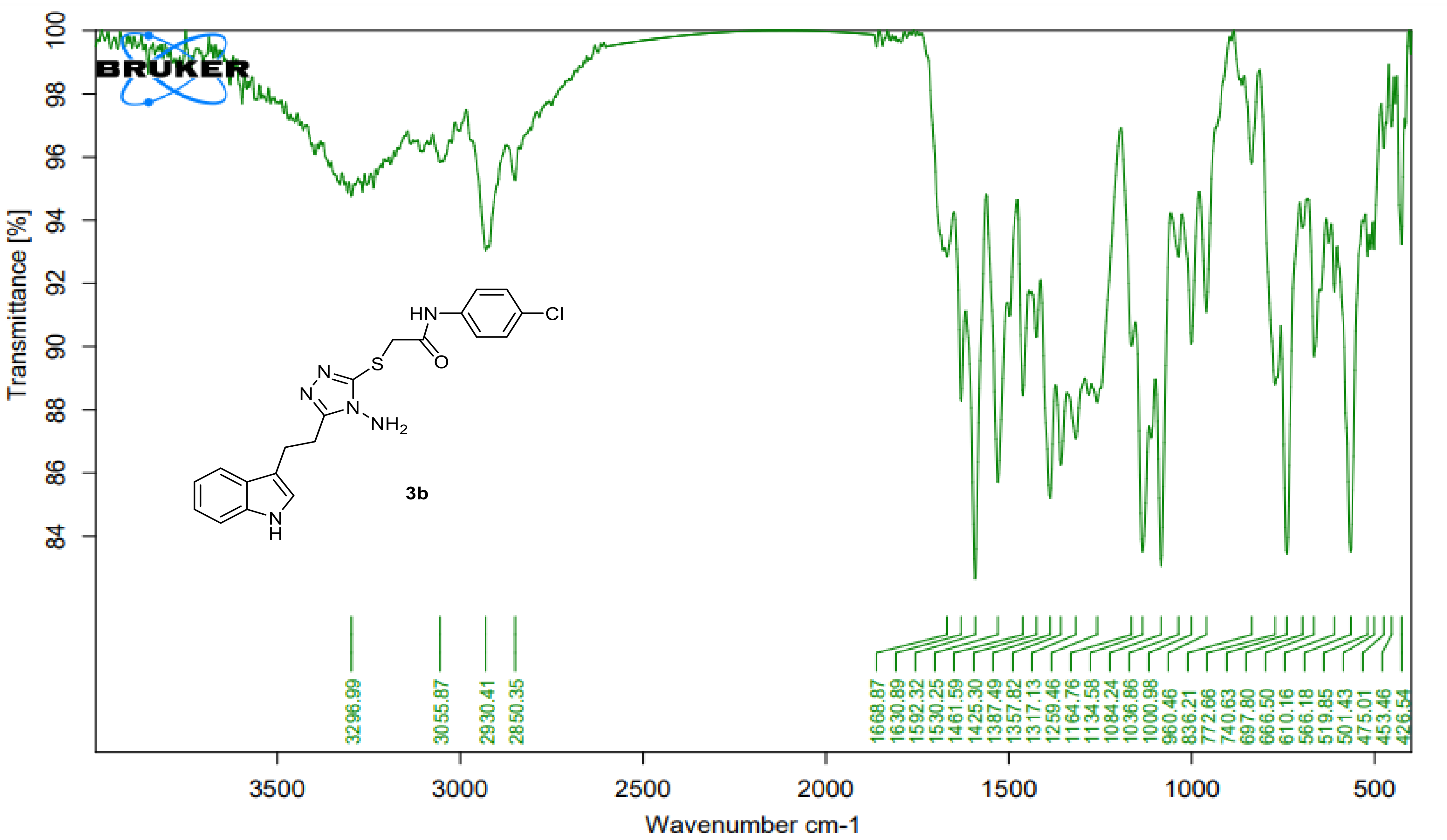
BRUKER
Transmittance [%]
100
98
96
94
92
90
88
86
84
HN
Cl
S
O
N
N
N
NH2
N
H
3b
3296.99
3055.87
2930.41
2850.35
1668.87
1630.89
1592.32
1530.25
1461.59
1425.30
1387.49
1357.82
1317.13
1259.46
1164.76
1134.58
1084.24
1036.86
1000.98
960.46
836.21
772.66
740.63
697.80
666.50
610.16
566.18
519.85
501.43
475.01
453.46
426.54
3500
3000
2500
2000
1500
1000
500
Wavenumber cm-1

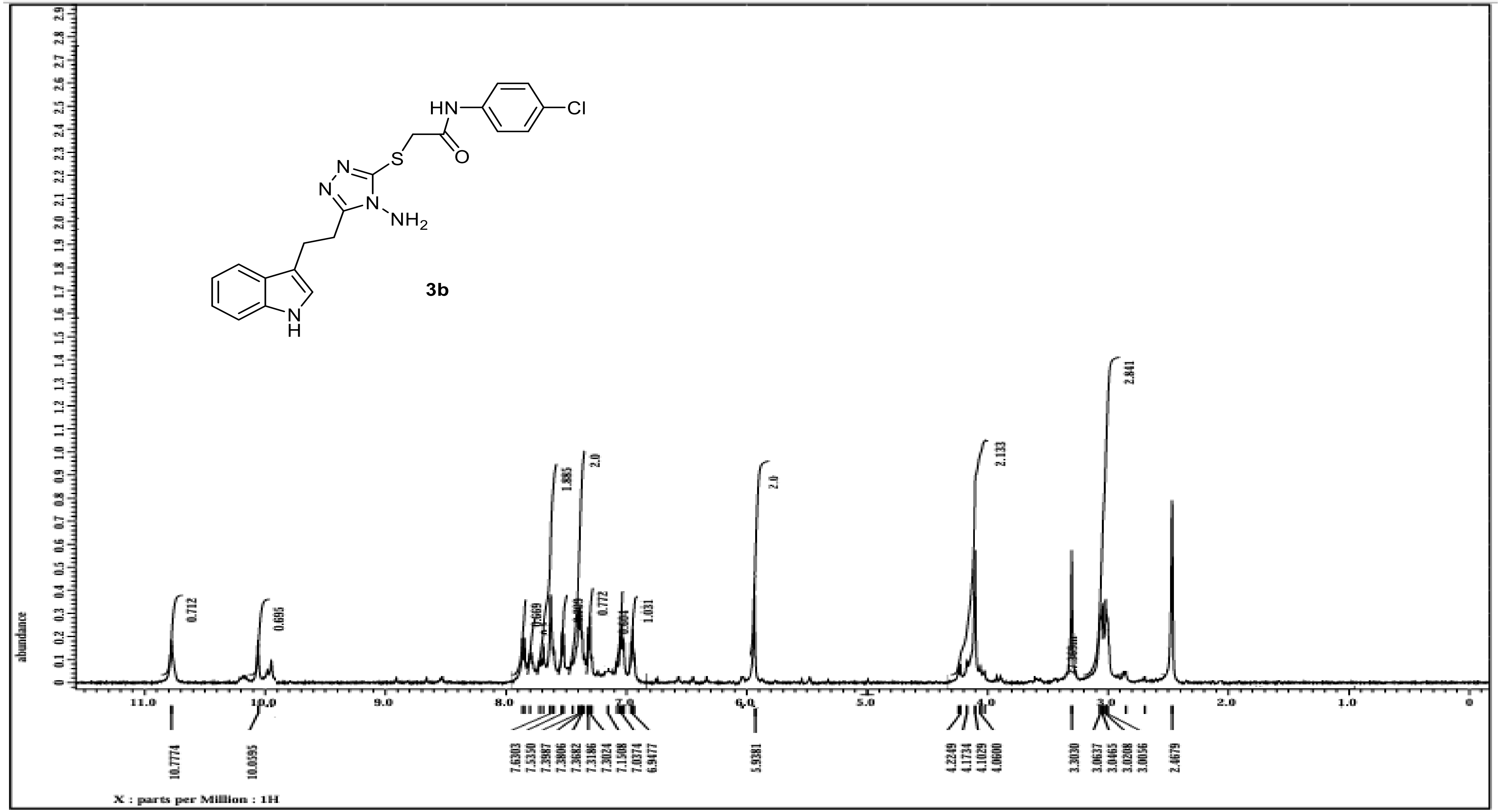

HN
Cl
S
O
N
N
N
NH2
N
H
3b
abundance
X : parts per Million : 1H
0.712
0.695
1.885
2.0
0.772
1.031
2.0
2.133
2.841
10.7774
10.0595
7.6303
7.5350
7.3987
7.3806
7.3682
7.3186
7.3024
7.1508
7.0374
6.9477
5.9381
4.2249
4.1734
4.1029
4.0600
3.3030
3.0637
3.0465
3.0208
3.0056
2.4679

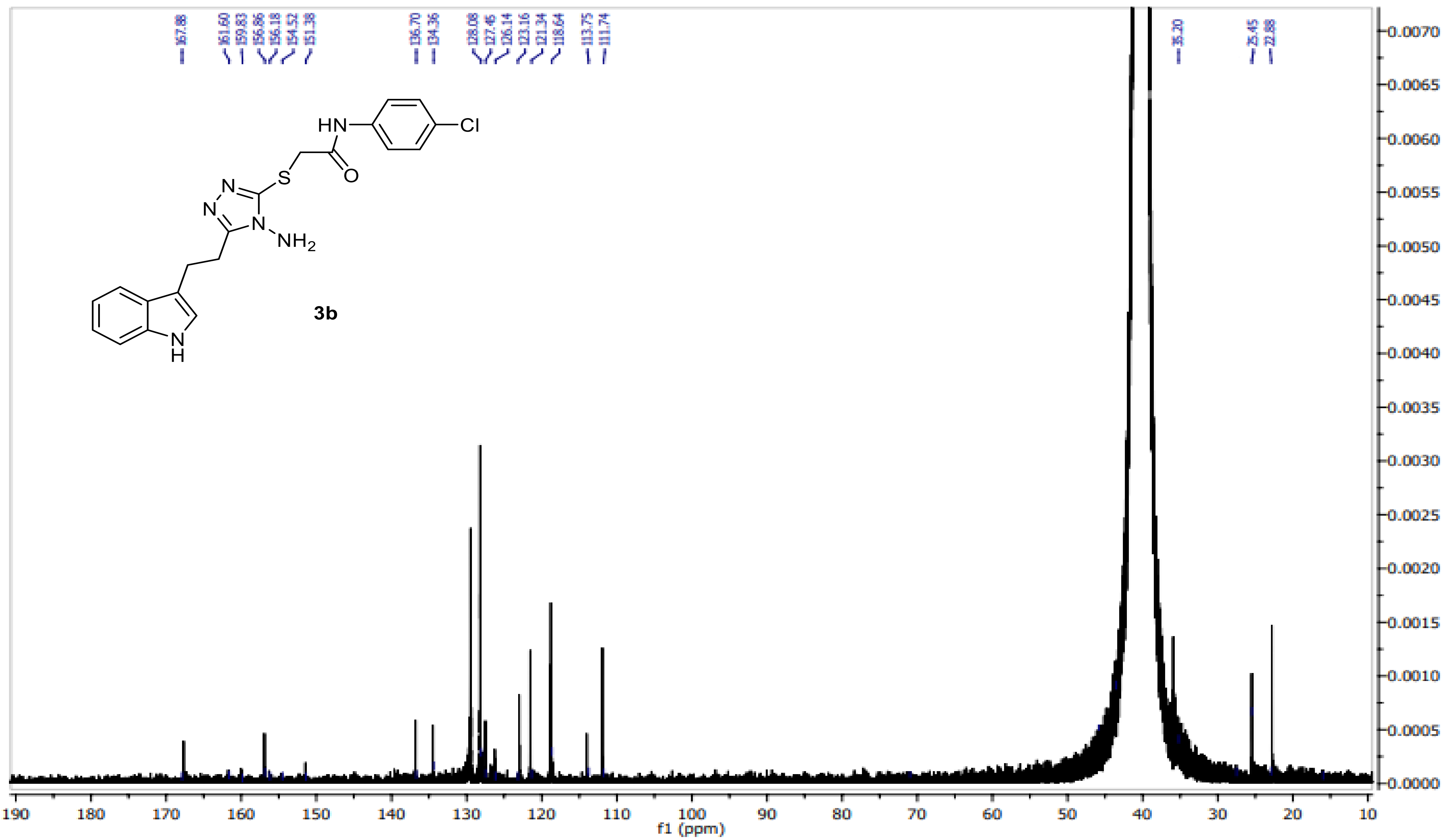

167.88
161.60
159.83
156.86
156.18
154.52
151.38
136.70
134.36
128.08
127.46
126.14
123.16
121.34
118.64
113.75
111.74
35.20
25.45
22.88
HN
Cl
S
O
N
N
N
NH2
N
H
3b
f1 (ppm)

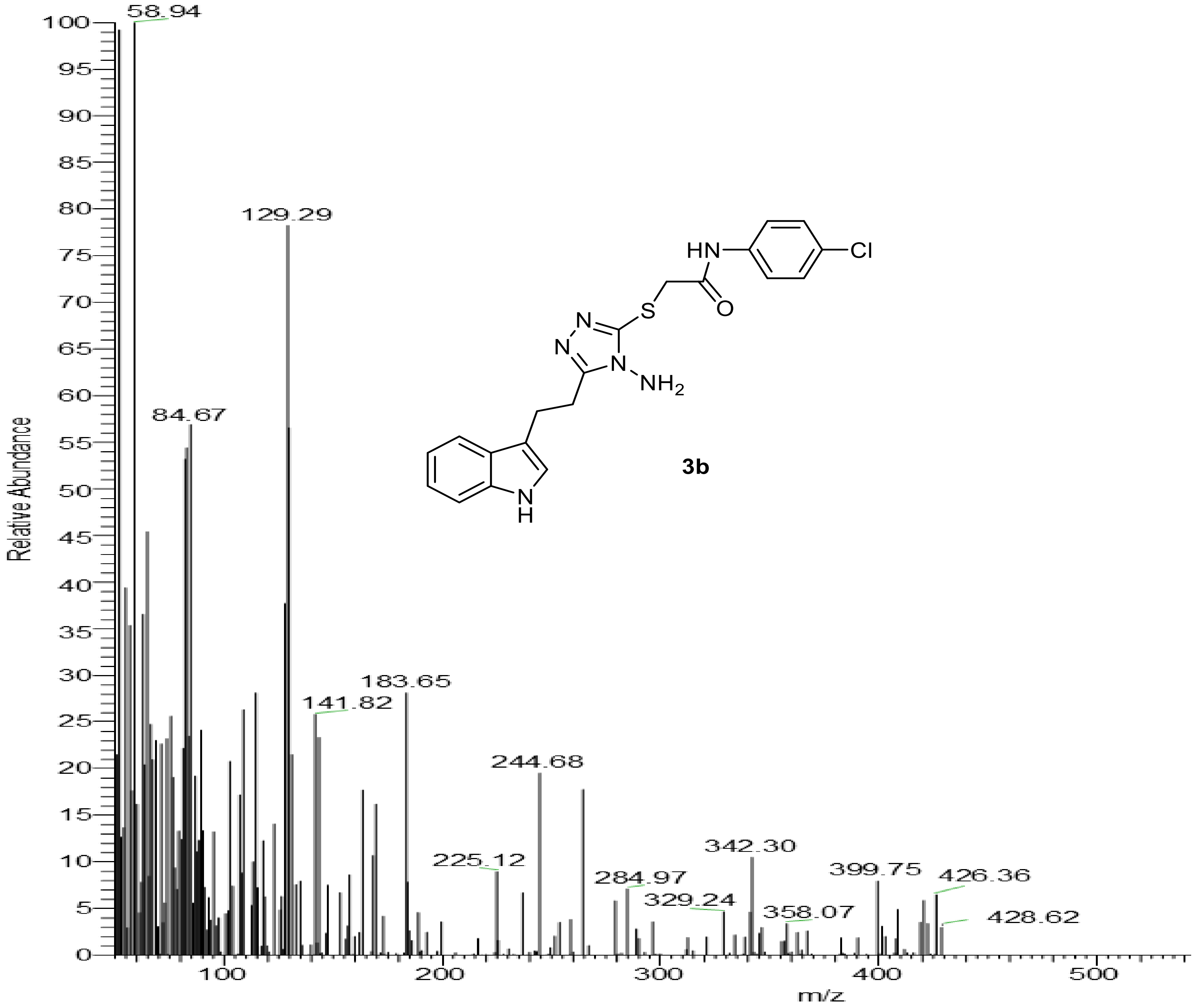
58.94
129.29
84.67
141.82
183.65
244.68
225.12
284.97
342.30
329.24
358.07
399.75
426.36
428.62
HN
Cl
S
O
N
N
N
N
NH2
N
H
3b
Relative Abundance
m/z

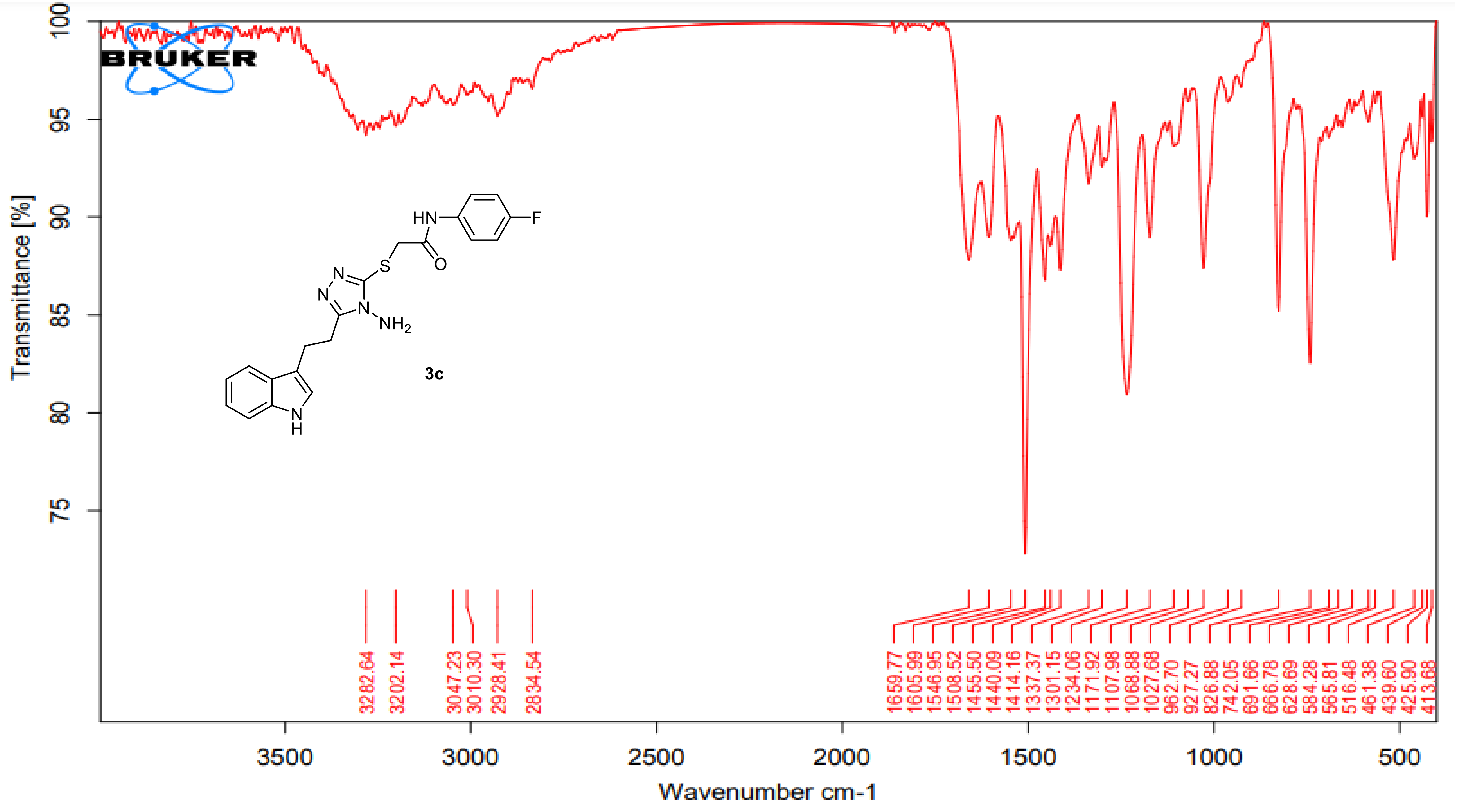
BRUKER
Transmittance [%]
100
95
90
85
80
75
3c
3282.64
3202.14
3047.23
3010.30
2928.41
2834.54
1659.77
1605.99
1546.95
1508.52
1455.50
1440.09
1414.16
1337.37
1301.15
1234.06
1171.92
1107.98
1068.88
1027.68
962.70
927.27
826.88
742.05
691.66
666.78
628.69
584.28
565.81
516.48
461.38
439.60
425.90
413.68
3500
3000
2500
2000
1500
1000
500
Wavenumber cm-1

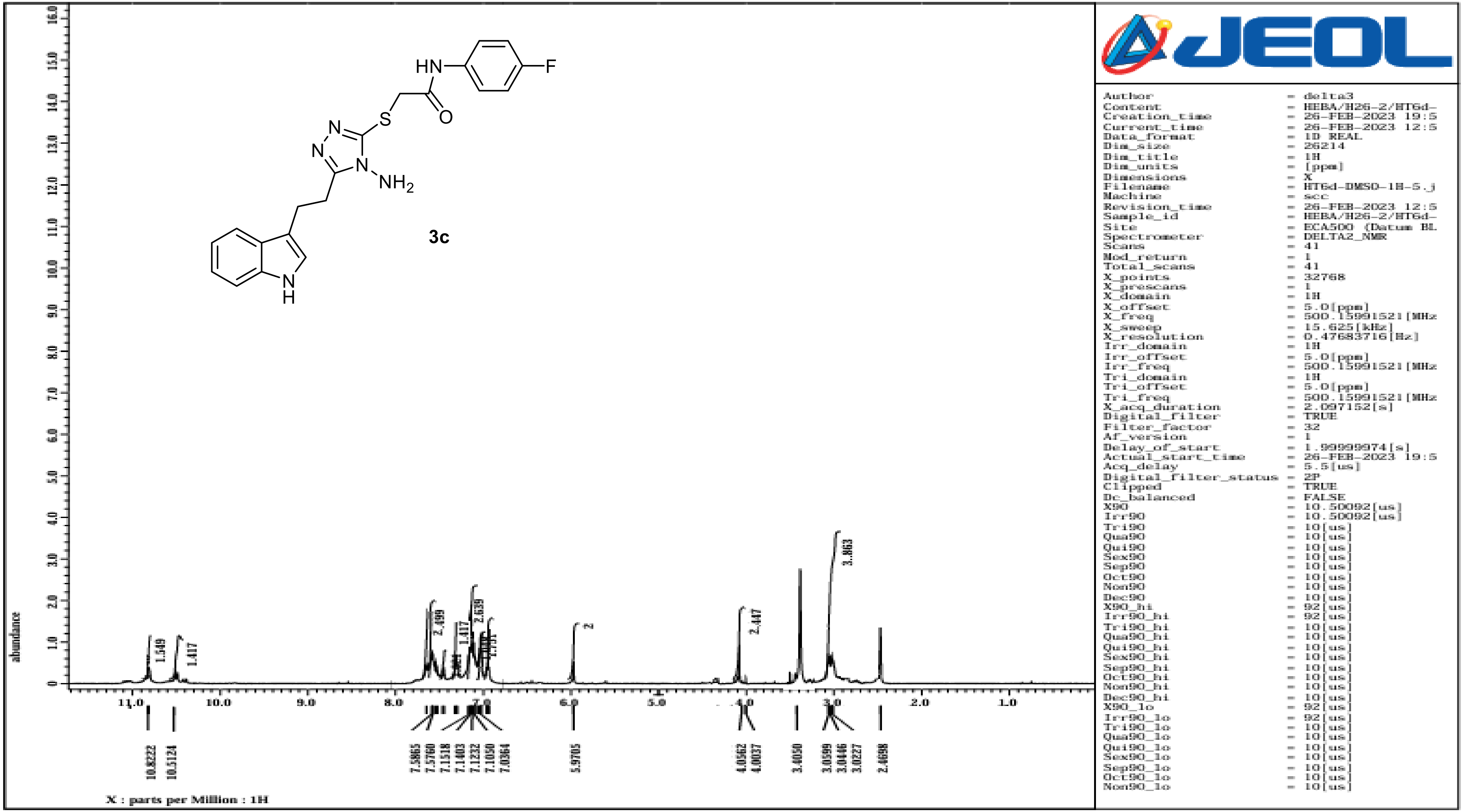
3c
HN
F
S
O
N
N
N
NH2
NH
JEOL
Author = delta3
Content = HEBA/H26-2/HT6d-
Creation_time = 26-FEB-2023 19:5
Current_time = 26-FEB-2023 12:5
Data_format = 1D REAL
Dim_size = 26214
Dim_title = 1H
Dim_units = [ppm]
Dimensions = X
Filename = HT6d-DMSO-1H-5.j
Machine = scc
Revision_time = 26-FEB-2023 12:5
Sample_id = HEBA/H26-2/HT6d-
Site = ECA500 (Datum BL
Spectrometer = DELTA2_NMR
Scans = 41
Mod_return = 1
Total_scans = 41
X_points = 32768
X_prescans = 1
X_domain = 1H
X_offset = 5.0[ppm]
X_freq = 500.15991521[MHz
X_sweep = 15.625[kHz]
X_resolution = 0.47683716[Hz]
Irr_domain = 1H
Irr_offset = 5.0[ppm]
Irr_freq = 500.15991521[MHz
Tri_domain = 1H
Tri_offset = 5.0[ppm]
Tri_freq = 500.15991521[MHz
X_acq_duration = 2.097152[s]
Digital_filter = TRUE
Filter_factor = 32
Af_version = 1
Delay_of_start = 1.99999974[s]
Actual_start_time = 26-FEB-2023 19:5
Acq_delay = 5.5[us]
Digital_filter_status = 2P
Clipped = TRUE
Dc_balanced = FALSE
X90 = 10.50092[us]
Irr90 = 10.50092[us]
Tri90 = 10[us]
Qua90 = 10[us]
Qui90 = 10[us]
Sex90 = 10[us]
Sep90 = 10[us]
Oct90 = 10[us]
Non90 = 10[us]
Dec90 = 10[us]
X90_hi = 92[us]
Irr90_hi = 92[us]
Tri90_hi = 10[us]
Qua90_hi = 10[us]
Qui90_hi = 10[us]
Sex90_hi = 10[us]
Sep90_hi = 10[us]
Oct90_hi = 10[us]
Non90_hi = 10[us]
Dec90_hi = 10[us]
X90_lo = 92[us]
Irr90_lo = 92[us]
Tri90_lo = 10[us]
Qua90_lo = 10[us]
Qui90_lo = 10[us]
Sex90_lo = 10[us]
Sep90_lo = 10[us]
Oct90_lo = 10[us]
Non90_lo = 10[us]
abundance
X : parts per Million : 1H
1.549
1.417
2.499
1.417
2.639
2
2.447
3.863
10.8222
10.5124
7.5865
7.5760
7.1518
7.1403
7.1232
7.1050
7.0364
5.9705
4.0562
4.0037
3.4050
3.0599
3.0446
3.0227
2.4698

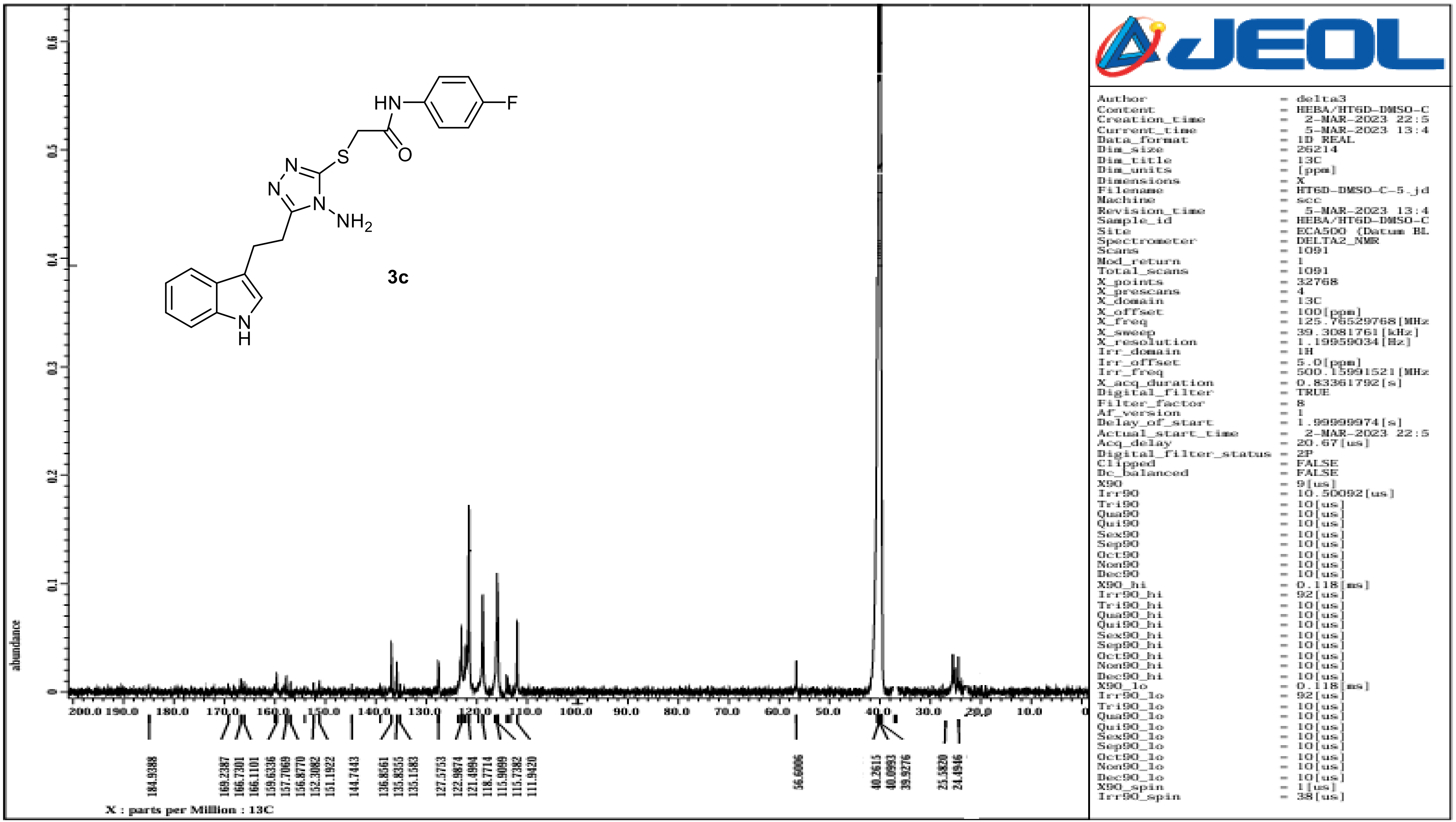

3c
HN
F
S
O
N
N
N
NH2
H
abundance
0
0.1
0.2
0.3
0.4
0.5
0.6
200.0 190.0 180.0 170.0 160.0 150.0 140.0 130.0 120.0 110.0 100.0 90.0 80.0 70.0 60.0 50.0 40.0 30.0 20.0 10.0 0
184.9388
169.2387
166.7301
166.1101
159.6336
157.7069
156.8770
152.3082
151.1922
144.7443
136.8561
135.8355
135.1583
127.5753
122.9874
121.4994
118.7714
115.9099
115.7382
111.9420
56.6006
40.2615
40.0993
39.9276
25.5820
24.4946
X : parts per Million : 13C
JEOL
Author = delta3
Content = HEBA/HT6D-DMSO-C
Creation_time = 2-MAR-2023 22:5
Current_time = 5-MAR-2023 13:4
Data_format = 1D REAL
Dim_size = 26214
Dim_title = 13C
Dim_units = [ppm]
Dimensions = X
Filename = HT6D-DMSO-C-5.jd
Machine = scc
Revision_time = 5-MAR-2023 13:4
Sample_id = HEBA/HT6D-DMSO-C
Site = ECA500 (Datum BL
Spectrometer = DELTA2_NMR
Scans = 1091
Mod_return = 1
Total_scans = 1091
X_points = 32768
X_prescans = 4
X_domain = 13C
X_offset = 100[ppm]
X_freq = 125.76529768[MHz
X_sweep = 39.3081761[kHz]
X_resolution = 1.19959034[Hz]
Irr_domain = 1H
Irr_offset = 5.0[ppm]
Irr_freq = 500.15991521[MHz
X_acq_duration = 0.83361792[s]
Digital_filter = TRUE
Filter_factor = 8
Af_version = 1
Delay_of_start = 1.99999974[s]
Actual_start_time = 2-MAR-2023 22:5
Acq_delay = 20.67[us]
Digital_filter_status = 2P
Clipped = FALSE
Dc_balanced = FALSE
X90 = 9[us]
Irr90 = 10.50092[us]
Tri90 = 10[us]
Qua90 = 10[us]
Qui90 = 10[us]
Sex90 = 10[us]
Sep90 = 10[us]
Oct90 = 10[us]
Non90 = 10[us]
Dec90 = 10[us]
X90_hi = 0.118[ms]
Irr90_hi = 92[us]
Tri90_hi = 10[us]
Qua90_hi = 10[us]
Qui90_hi = 10[us]
Sex90_hi = 10[us]
Sep90_hi = 10[us]
Oct90_hi = 10[us]
Non90_hi = 10[us]
Dec90_hi = 10[us]
X90_lo = 0.118[ms]
Irr90_lo = 92[us]
Tri90_lo = 10[us]
Qua90_lo = 10[us]
Qui90_lo = 10[us]
Sex90_lo = 10[us]
Sep90_lo = 10[us]
Oct90_lo = 10[us]
Non90_lo = 10[us]
Dec90_lo = 10[us]
X90_spin = 1[us]
Irr90_spin = 38[us]

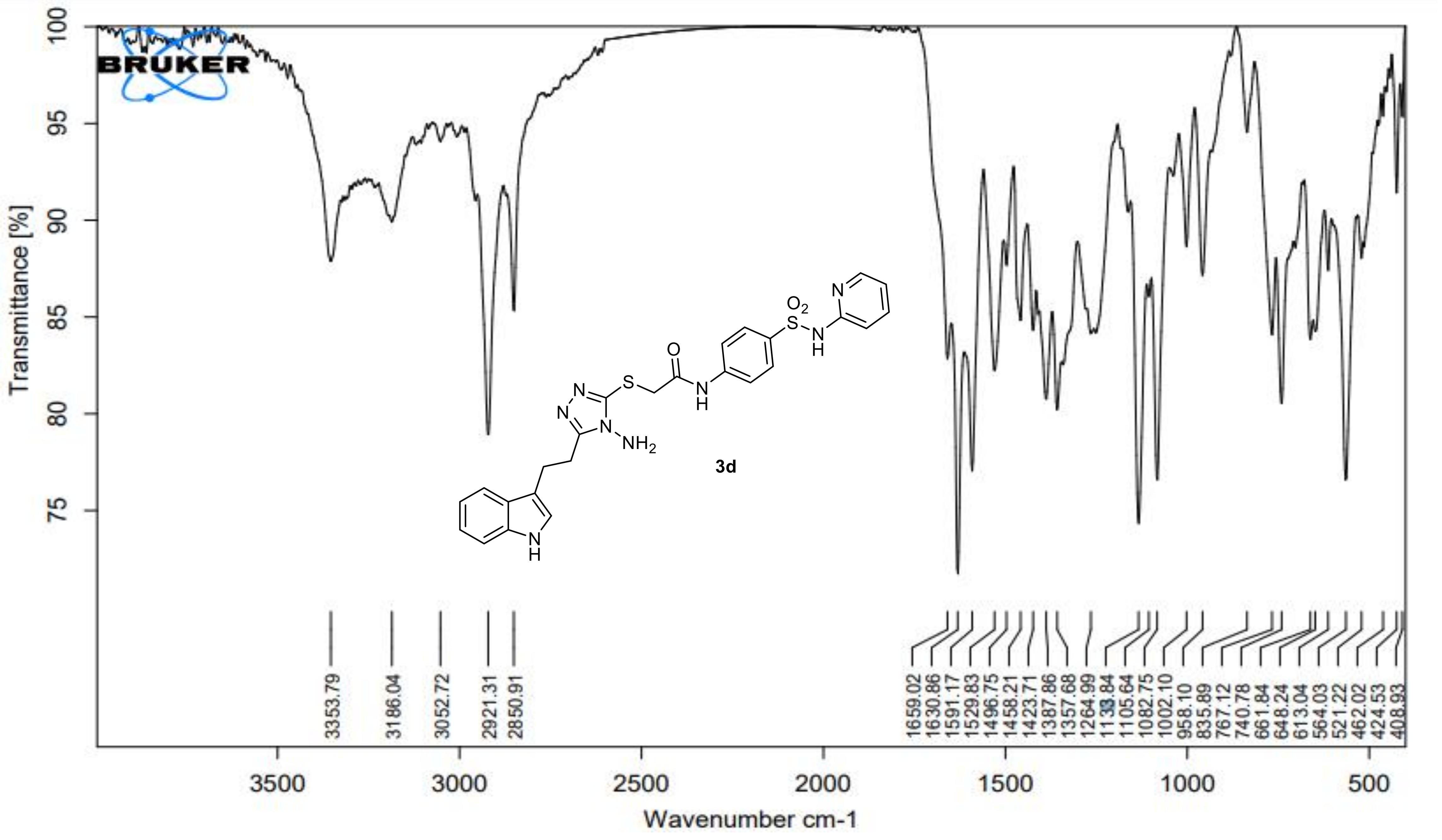

BRUKER
Transmittance [%]
100
95
90
85
80
75
Wavenumber cm-1
3500
3000
2500
2000
1500
1000
500
3353.79
3186.04
3052.72
2921.31
2850.91
1659.02
1630.86
1591.17
1529.83
1496.75
1458.21
1423.71
1387.86
1357.68
1264.99
1133.84
1105.64
1082.75
1002.10
958.10
835.89
767.12
740.78
661.84
648.24
613.04
564.03
521.22
462.02
424.53
408.93
3d

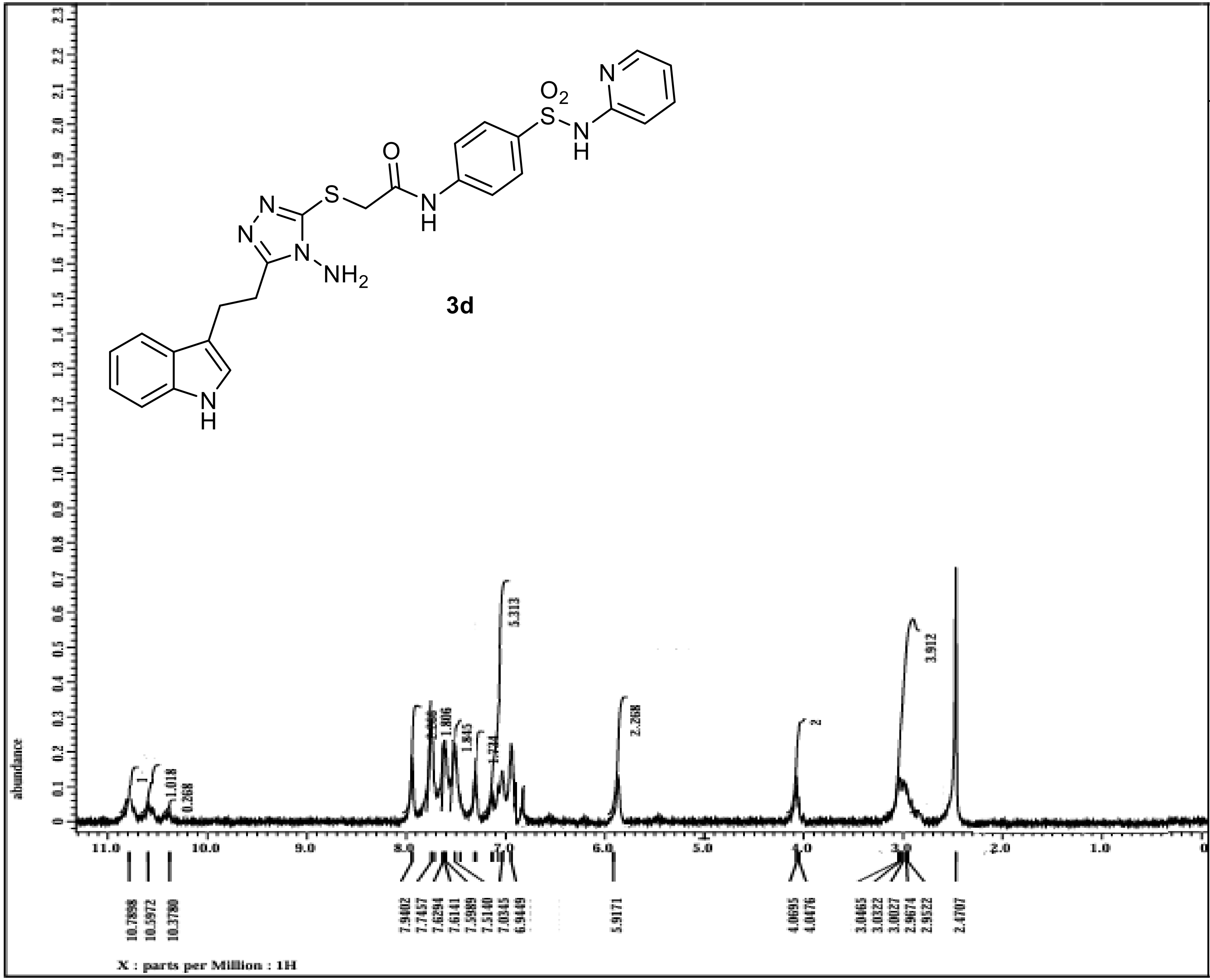

Author	=	delta3
Content	=	HEBA/H26-2/HT6F-
Creation_time	=	26-FEB-2023 21:2
Current_time	=	26-FEB-2023 14:1
Data_format	=	1D REAL
Dim_size	=	26214
Dim_title	=	1H
Dim_units	=	[ppm]
Dimensions	=	X
Filename	=	HT6F-DMSO-1H-5.j
Machine	=	scc
Revision_time	=	26-FEB-2023 14:1
Sample_id	=	HEBA/H26-2/HT6F-
Site	=	ECA500 (Datum BL
Spectrometer	=	DELTA2_NMR
Scans	=	27
Mod_return	=	1
Total_scans	=	27
X_points	=	32768
X_prescans	=	1
X_domain	=	1H
X_offset	=	5.0[ppm]
X_freq	=	500.15991521[MHz
X_sweep	=	15.625[kHz]
X_resolution	=	0.47683716[Hz]
Irr_domain	=	1H
Irr_offset	=	5.0[ppm]
Irr_freq	=	500.15991521[MHz
Tri_domain	=	1H
Tri_offset	=	5.0[ppm]
Tri_freq	=	500.15991521[MHz
X_acq_duration	=	2.097152[s]
Digital_filter	=	TRUE
Filter_factor	=	32
Af_version	=	1
Delay_of_start	=	1.99999974[s]
Actual_start_time	=	26-FEB-2023 21:2
Acq_delay	=	5.5[us]
Digital_filter_status	=	2P
Clipped	=	TRUE
Dc_balanced	=	FALSE
X90	=	10.50092[us]
Irr90	=	10.50092[us]
Tri90	=	10[us]
Qua90	=	10[us]
Qui90	=	10[us]
Sex90	=	10[us]
Sep90	=	10[us]
Oct90	=	10[us]
Non90	=	10[us]
Dec90	=	10[us]
X90_hi	=	92[us]
Irr90_hi	=	92[us]
Tri90_hi	=	10[us]
Qua90_hi	=	10[us]
Qui90_hi	=	10[us]
Sex90_hi	=	10[us]
Sep90_hi	=	10[us]
Oct90_hi	=	10[us]
Non90_hi	=	10[us]
Dec90_hi	=	10[us]
X90_lo	=	92[us]
Irr90_lo	=	92[us]
Tri90_lo	=	10[us]
Qua90_lo	=	10[us]
Qui90_lo	=	10[us]
Sex90_lo	=	10[us]
Sep90_lo	=	10[us]
Oct90_lo	=	10[us]
Non90_lo	=	10[us]

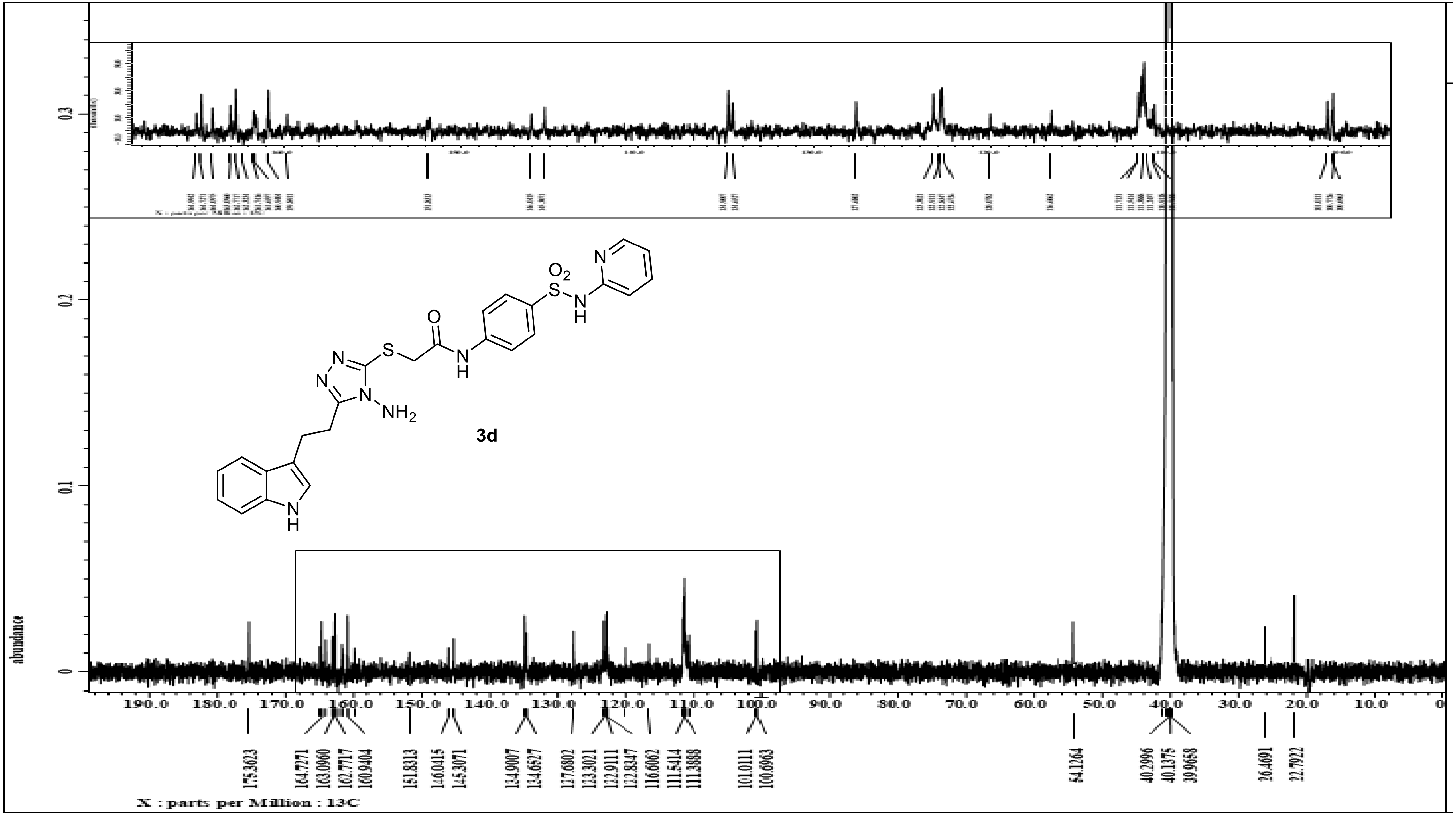

abundance
0.3
0.2
0.1
0
3d
175.3623
164.7271
163.0960
162.7717
160.9404
151.8313
146.0415
145.3071
134.9007
134.6527
127.6802
123.3021
122.9111
122.8347
116.6062
111.5414
111.3888
101.0111
100.6963
54.1264
40.2996
40.1375
39.9658
26.4691
22.7922
X : parts per Million : 13C

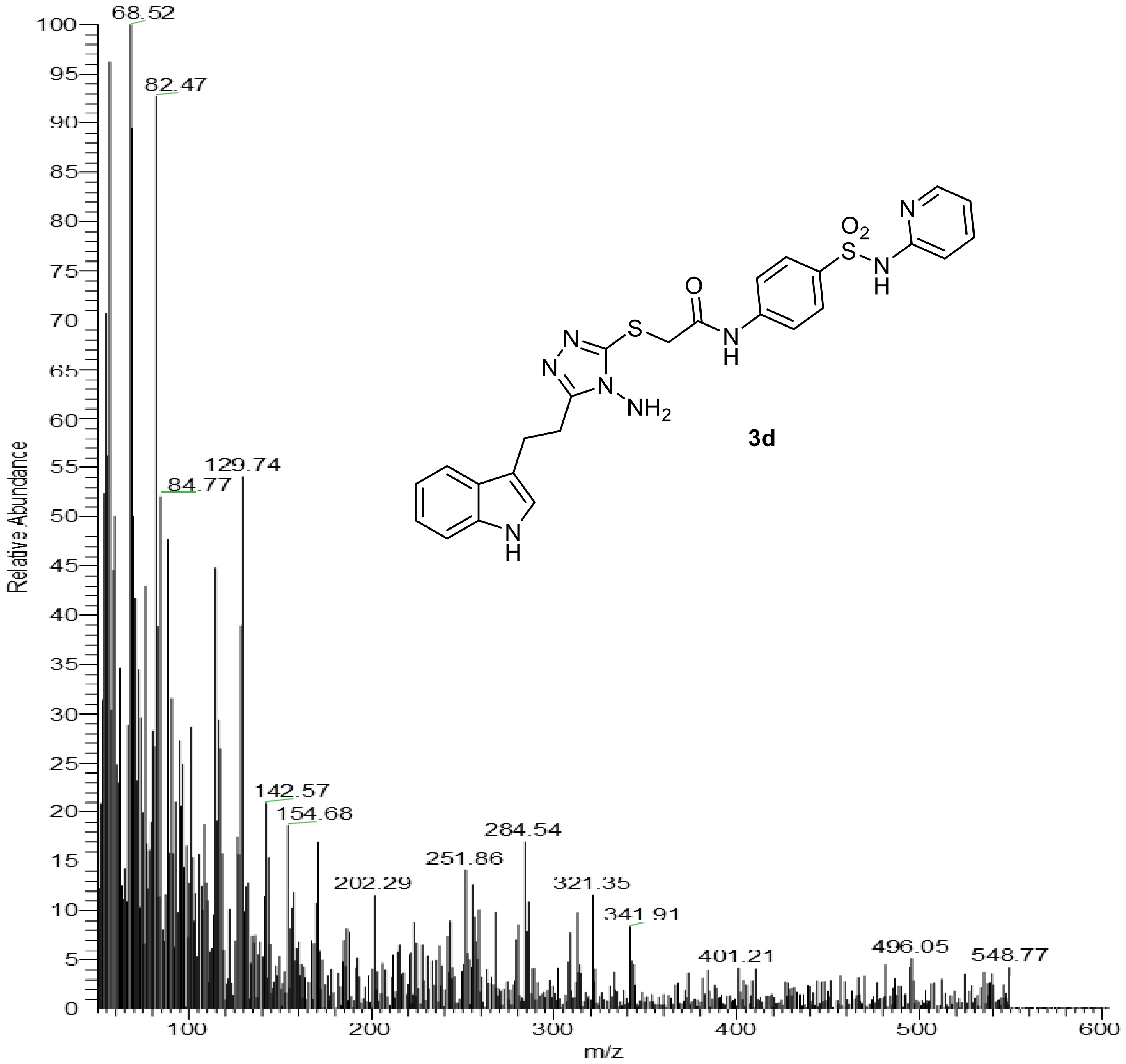
68.52
82.47
84.77
129.74
142.57
154.68
202.29
251.86
284.54
321.35
341.91
401.21
496.05
548.77
Relative Abundance
m/z
O
O2
S
N
H
NH2
3d

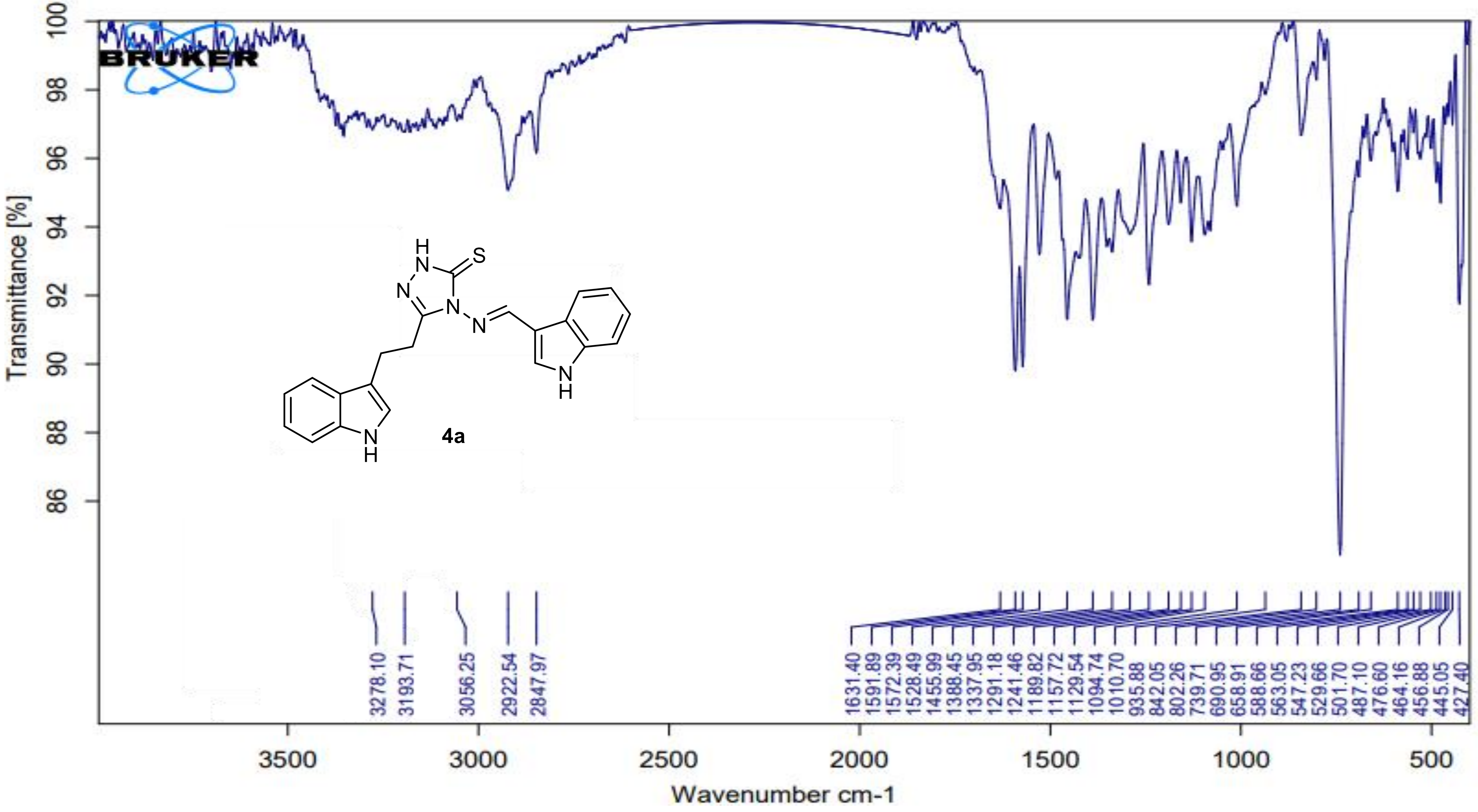

Transmittance [%]
100
98
96
94
92
90
88
86
BRUKER
4a
3278.10
3193.71
3056.25
2922.54
2847.97
1631.40
1591.89
1572.39
1528.49
1455.99
1388.45
1337.95
1291.18
1241.46
1189.82
1157.72
1129.54
1094.74
1010.70
935.88
842.05
802.26
739.71
690.95
658.91
588.66
563.05
547.23
529.66
501.70
487.10
476.60
464.16
456.88
445.05
427.40
3500
3000
2500
2000
1500
1000
500
Wavenumber cm-1

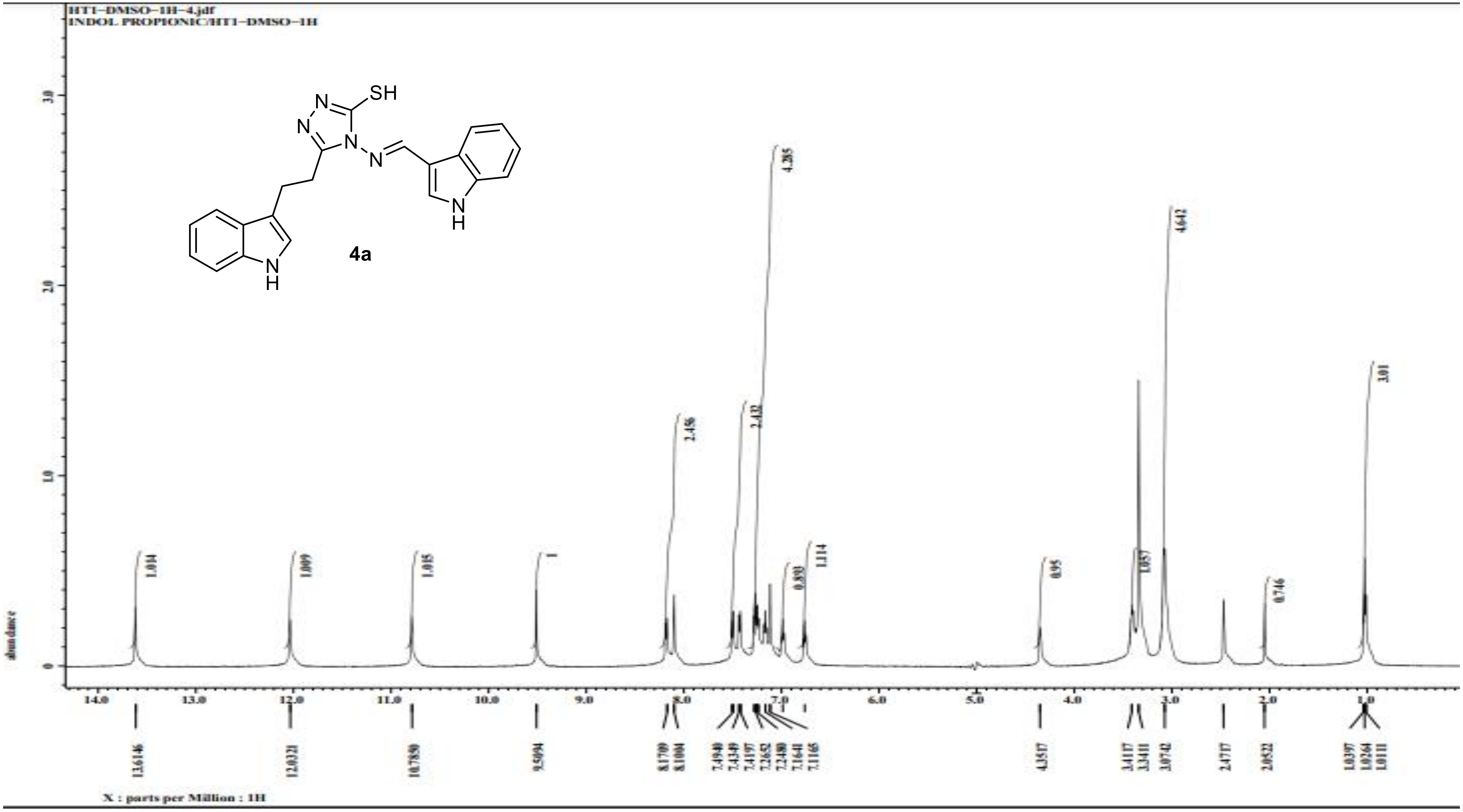
HT1–DMSO–1H–4.jdf
INDOL PROPIONIC/HT1–DMSO–1H
SH
4a
abundance
X : parts per Million : 1H

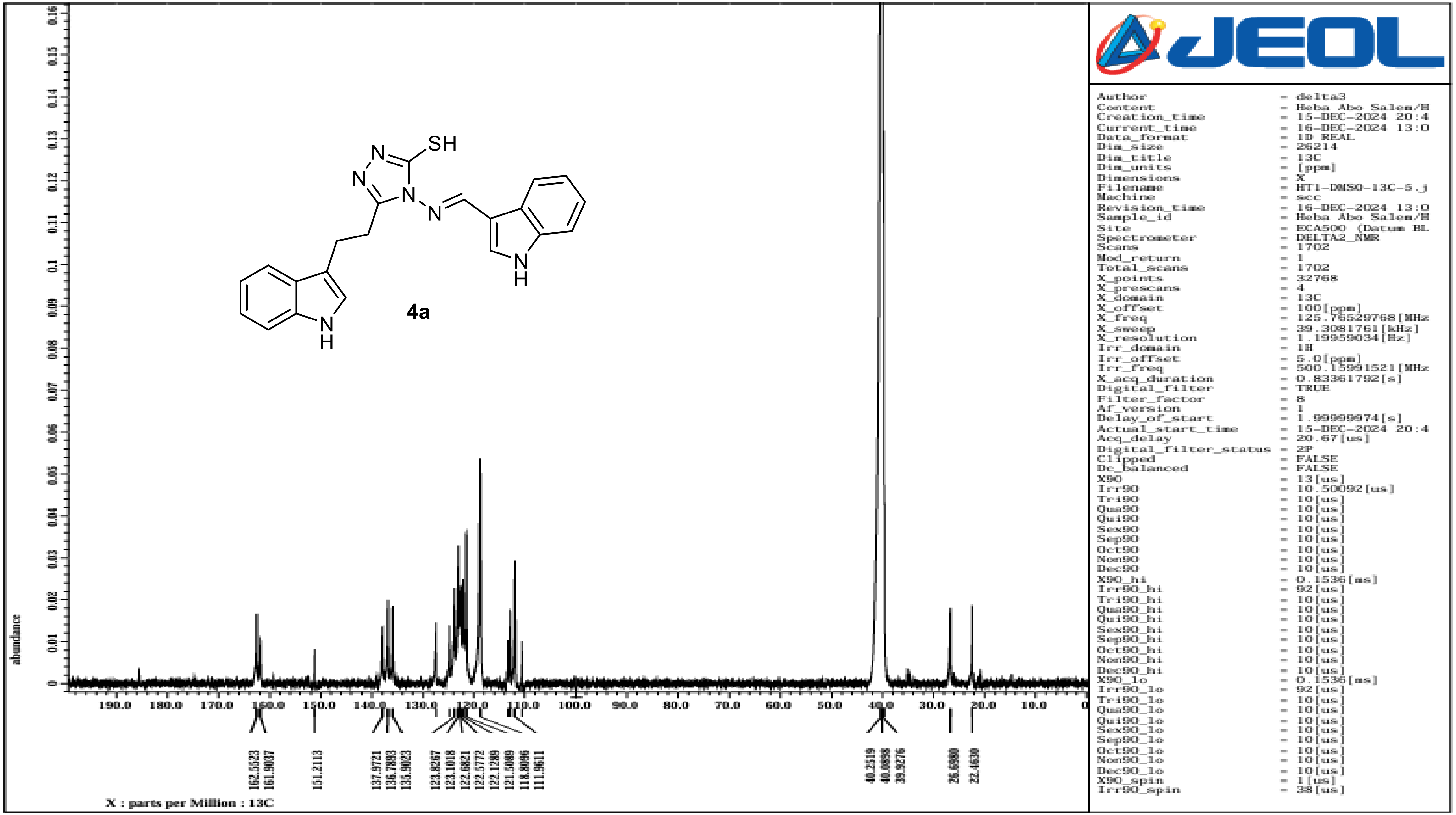
SH
N
N
N
N
N
N
H
N
H
4a
JEOL
abundance
X : parts per Million : 13C
162.5523
161.9037
151.2113
137.9721
136.7893
135.9023
123.8267
123.1018
122.6821
122.5772
122.1289
121.5089
118.8096
111.9611
40.2519
40.0898
39.9276
26.6980
22.4630
Author = delta3
Content = Heba Abo Salem/H
Creation_time = 15-DEC-2024 20:4
Current_time = 16-DEC-2024 13:0
Data_format = 1D REAL
Dim_size = 26214
Dim_title = 13C
Dim_units = [ppm]
Dimensions = X
Filename = HT1-DMSO-13C-5.j
Machine = scc
Revision_time = 16-DEC-2024 13:0
Sample_id = Heba Abo Salem/H
Site = ECA500 (Datum BL
Spectrometer = DELTA2_NMR
Scans = 1702
Mod_return = 1
Total_scans = 1702
X_points = 32768
X_prescans = 4
X_domain = 13C
X_offset = 100[ppm]
X_freq = 125.76529768[MHz
X_sweep = 39.3081761[kHz]
X_resolution = 1.19959034[Hz]
Irr_domain = 1H
Irr_offset = 5.0[ppm]
Irr_freq = 500.15991521[MHz
X_acq_duration = 0.83361792[s]
Digital_filter = TRUE
Filter_factor = 8
Af_version = 1
Delay_of_start = 1.99999974[s]
Actual_start_time = 15-DEC-2024 20:4
Acq_delay = 20.67[us]
Digital_filter_status = 2P
Clipped = FALSE
Dc_balanced = FALSE
X90 = 13[us]
Irr90 = 10.50092[us]
Tri90 = 10[us]
Qua90 = 10[us]
Qui90 = 10[us]
Sex90 = 10[us]
Sep90 = 10[us]
Oct90 = 10[us]
Non90 = 10[us]
Dec90 = 10[us]
X90_hi = 0.1536[ms]
Irr90_hi = 92[us]
Tri90_hi = 10[us]
Qua90_hi = 10[us]
Qui90_hi = 10[us]
Sex90_hi = 10[us]
Sep90_hi = 10[us]
Oct90_hi = 10[us]
Non90_hi = 10[us]
Dec90_hi = 10[us]
X90_lo = 0.1536[ms]
Irr90_lo = 92[us]
Tri90_lo = 10[us]
Qua90_lo = 10[us]
Qui90_lo = 10[us]
Sex90_lo = 10[us]
Sep90_lo = 10[us]
Oct90_lo = 10[us]
Non90_lo = 10[us]
Dec90_lo = 10[us]
X90_spin = 1[us]
Irr90_spin = 38[us]

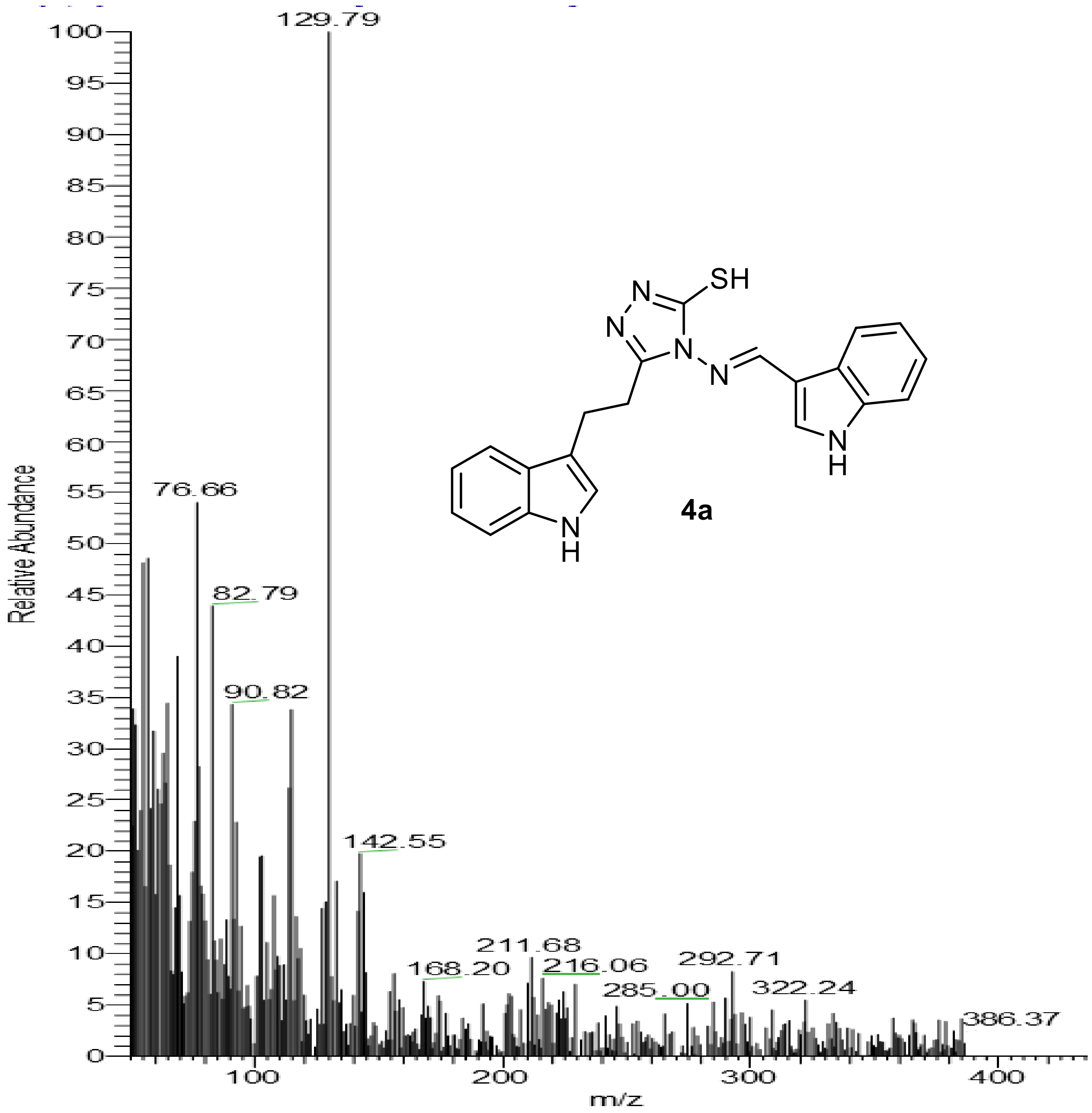
129.79
76.66
82.79
90.82
142.55
168.20
211.68
216.06
285.00
292.71
322.24
386.37
Relative Abundance
m/z
SH
N
N
N
N
NH
NH
4a

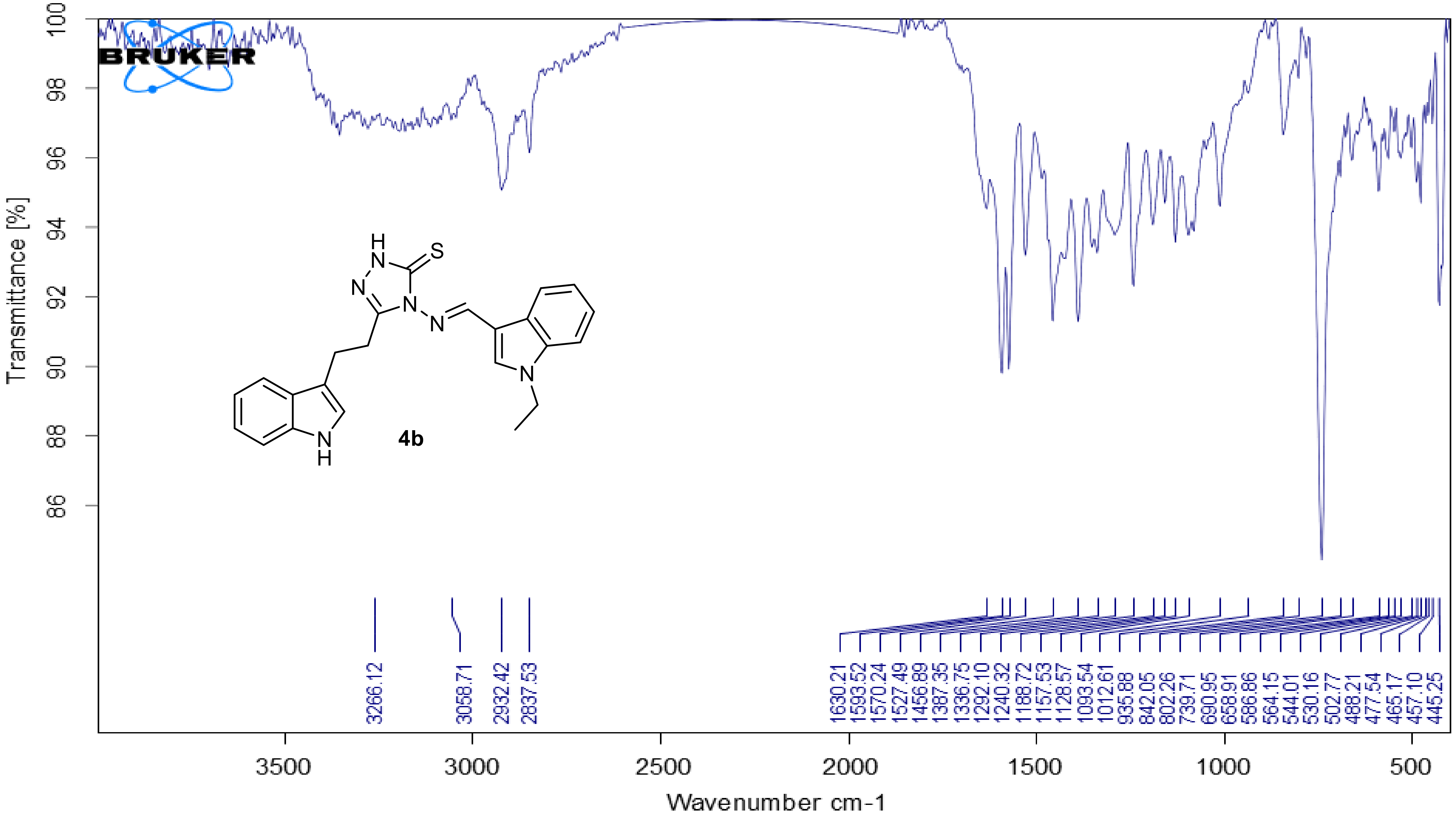
BRUKER
Transmittance [%]
Wavenumber cm-1
3266.12
3058.71
2932.42
2837.53
1630.21
1593.52
1570.24
1527.49
1456.89
1387.35
1336.75
1292.10
1240.32
1188.72
1157.53
1128.57
1093.54
1012.61
935.88
842.05
802.26
739.71
690.95
658.91
586.86
564.15
544.01
530.16
502.77
488.21
477.54
465.17
457.10
445.25
4b

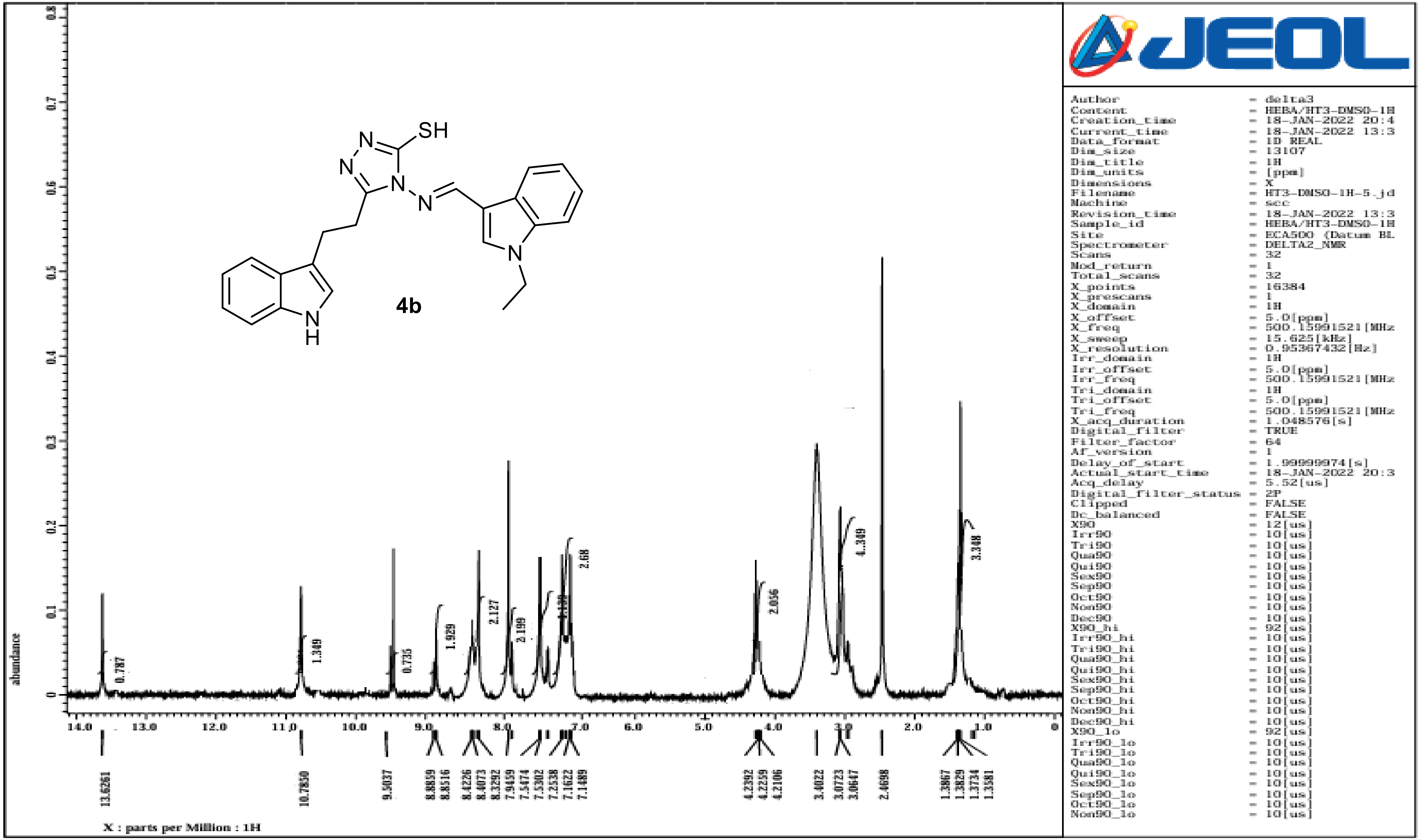

SH
N
N
N
N
N
N
H
4b
abundance
X : parts per Million : 1H
13.6261
10.7850
9.5037
8.8859
8.8516
8.4226
8.4073
8.3292
7.9459
7.5474
7.5302
7.2538
7.1622
7.1489
4.2392
4.2259
4.2106
3.4022
3.0723
3.0647
2.4698
1.3867
1.3829
1.3734
1.3581
0.787
1.349
0.735
1.929
2.127
2.199
2.68
2.056
4.349
3.348
JEOL
Author = delta3
Content = HEBA/HT3-DMSO-1H
Creation_time = 18-JAN-2022 20:4
Current_time = 18-JAN-2022 13:3
Data_format = 1D REAL
Dim_size = 13107
Dim_title = 1H
Dim_units = [ppm]
Dimensions = X
Filename = HT3-DMSO-1H-5.jd
Machine = scc
Revision_time = 18-JAN-2022 13:3
Sample_id = HEBA/HT3-DMSO-1H
Site = ECA500 (Datum BL
Spectrometer = DELTA2_NMR
Scans = 32
Mod_return = 1
Total_scans = 32
X_points = 16384
X_prescans = 1
X_domain = 1H
X_offset = 5.0[ppm]
X_freq = 500.15991521[MHz
X_sweep = 15.625[kHz]
X_resolution = 0.95367432[Hz]
Irr_domain = 1H
Irr_offset = 5.0[ppm]
Irr_freq = 500.15991521[MHz
Tri_domain = 1H
Tri_offset = 5.0[ppm]
Tri_freq = 500.15991521[MHz
X_acq_duration = 1.048576[s]
Digital_filter = TRUE
Filter_factor = 64
Af_version = 1
Delay_of_start = 1.99999974[s]
Actual_start_time = 18-JAN-2022 20:3
Acq_delay = 5.52[us]
Digital_filter_status = 2P
Clipped = FALSE
Dc_balanced = FALSE
X90 = 12[us]
Irr90 = 10[us]
Tri90 = 10[us]
Qua90 = 10[us]
Qui90 = 10[us]
Sex90 = 10[us]
Sep90 = 10[us]
Oct90 = 10[us]
Non90 = 10[us]
Dec90 = 10[us]
X90_hi = 92[us]
Irr90_hi = 10[us]
Tri90_hi = 10[us]
Qua90_hi = 10[us]
Qui90_hi = 10[us]
Sex90_hi = 10[us]
Sep90_hi = 10[us]
Oct90_hi = 10[us]
Non90_hi = 10[us]
Dec90_hi = 10[us]
X90_lo = 92[us]
Irr90_lo = 10[us]
Tri90_lo = 10[us]
Qua90_lo = 10[us]
Qui90_lo = 10[us]
Sex90_lo = 10[us]
Sep90_lo = 10[us]
Oct90_lo = 10[us]
Non90_lo = 10[us]

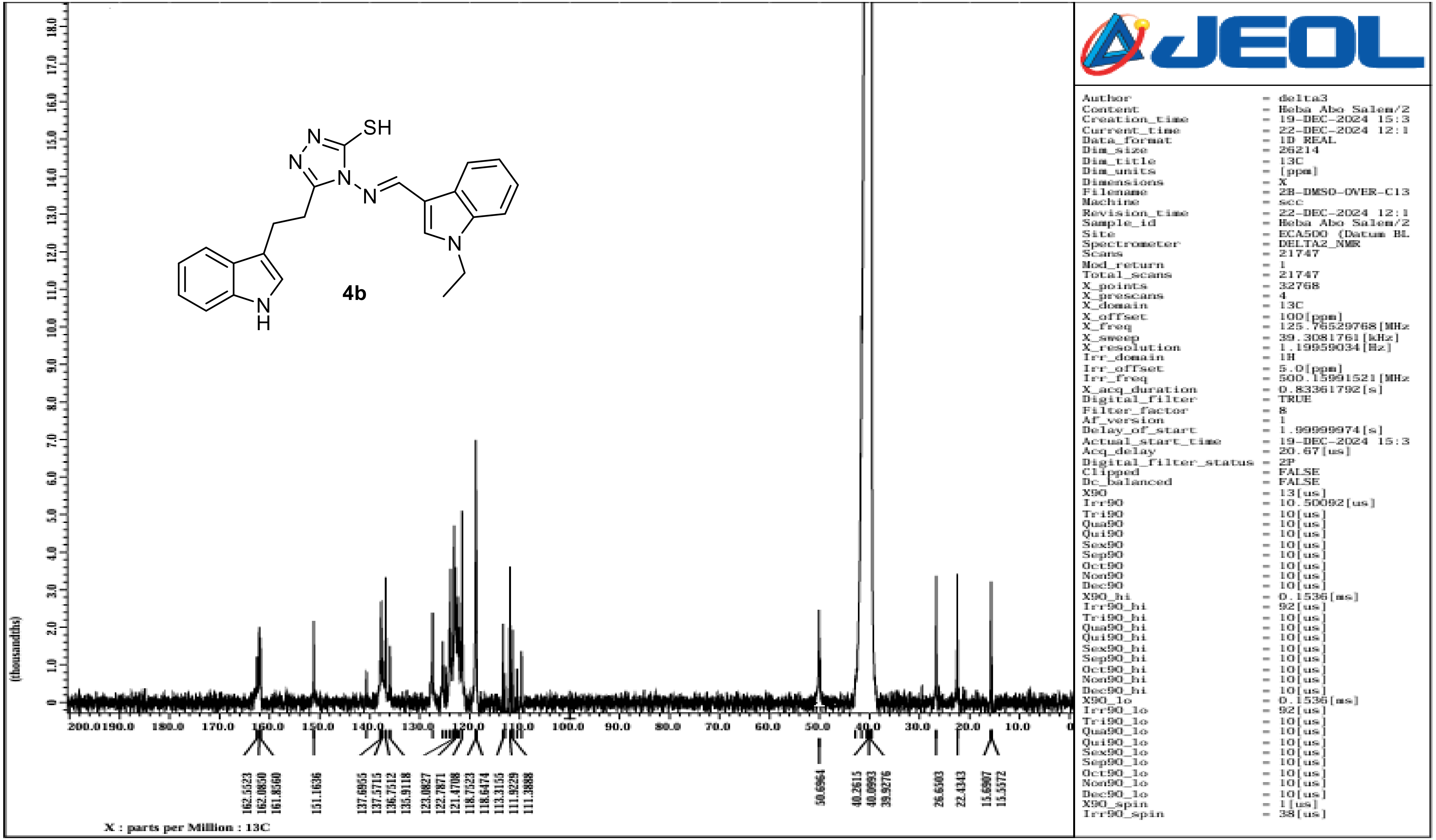
4b
SH
N
N
N
N
N
N
H
(thousandths)
X : parts per Million : 13C
162.5523
162.0850
161.8560
151.1636
137.6955
137.5715
136.7512
135.9118
123.0827
122.7871
121.4708
118.7523
118.6474
113.3155
111.9229
111.3888
50.6964
40.2615
40.0993
39.9276
26.6503
22.4343
15.6907
15.5372
Author = delta3
Content = Heba Abo Salem/2
Creation_time = 19-DEC-2024 15:3
Current_time = 22-DEC-2024 12:1
Data_format = 1D REAL
Dim_size = 26214
Dim_title = 13C
Dim_units = [ppm]
Dimensions = X
Filename = 2B-DMSO-OVER-C13
Machine = scc
Revision_time = 22-DEC-2024 12:1
Sample_id = Heba Abo Salem/2
Site = ECA500 (Datum BL
Spectrometer = DELTA2_NMR
Scans = 21747
Mod_return = 1
Total_scans = 21747
X_points = 32768
X_prescans = 4
X_domain = 13C
X_offset = 100[ppm]
X_freq = 125.76529768[MHz
X_sweep = 39.3081761[kHz]
X_resolution = 1.19959034[Hz]
Irr_domain = 1H
Irr_offset = 5.0[ppm]
Irr_freq = 500.15991521[MHz
X_acq_duration = 0.83361792[s]
Digital_filter = TRUE
Filter_factor = 8
Af_version = 1
Delay_of_start = 1.99999974[s]
Actual_start_time = 19-DEC-2024 15:3
Acq_delay = 20.67[us]
Digital_filter_status = 2P
Clipped = FALSE
Dc_balanced = FALSE
X90 = 13[us]
Irr90 = 10.50092[us]
X90_hi = 0.1536[ms]
Irr90_hi = 92[us]
X90_lo = 0.1536[ms]
Irr90_lo = 92[us]
X90_spin = 1[us]
Irr90_spin = 38[us]

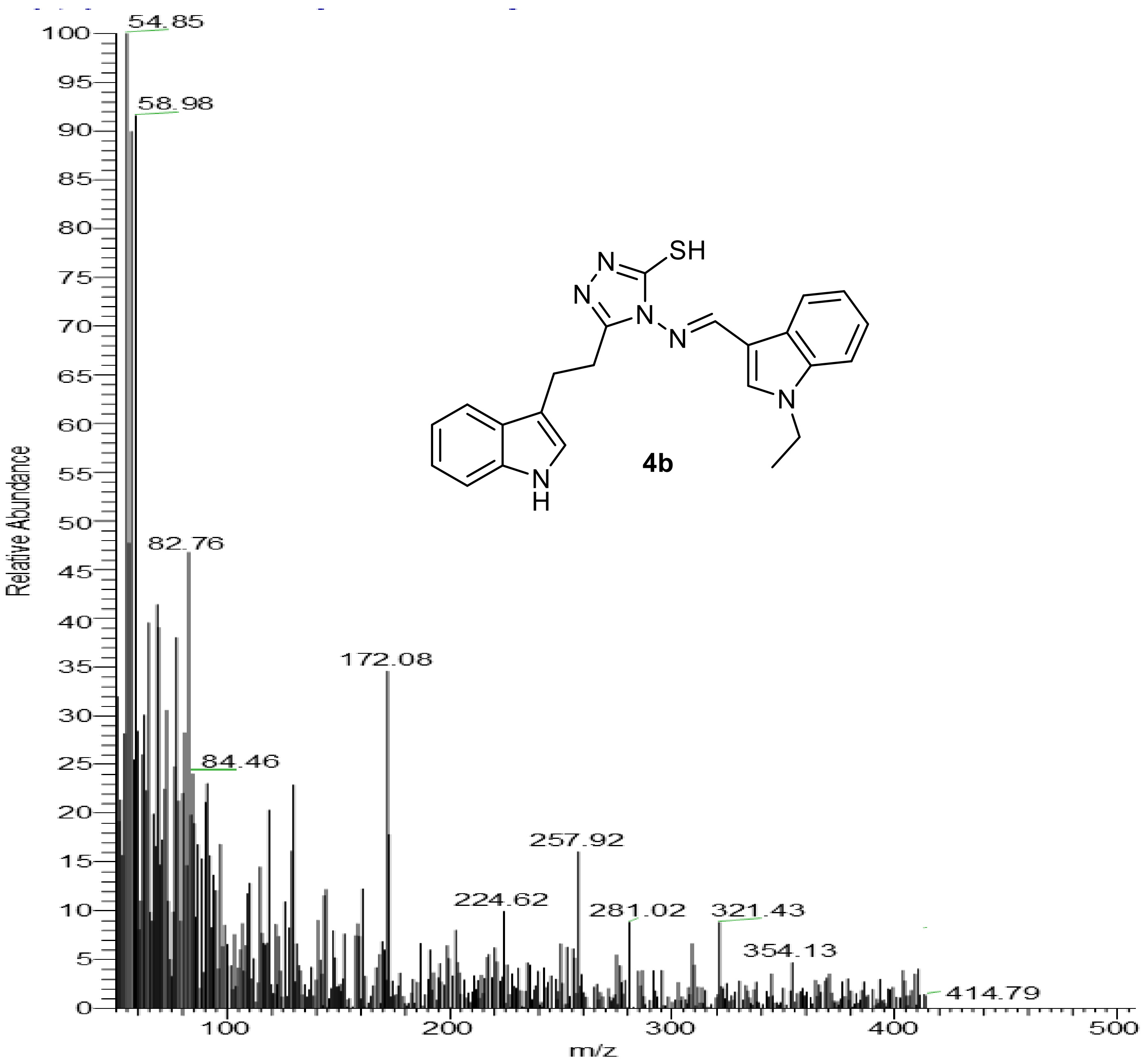
54.85
58.98
82.76
84.46
172.08
224.62
257.92
281.02
321.43
354.13
414.79
Relative Abundance
m/z
SH
N
N
N
N
N
NH
N
4b

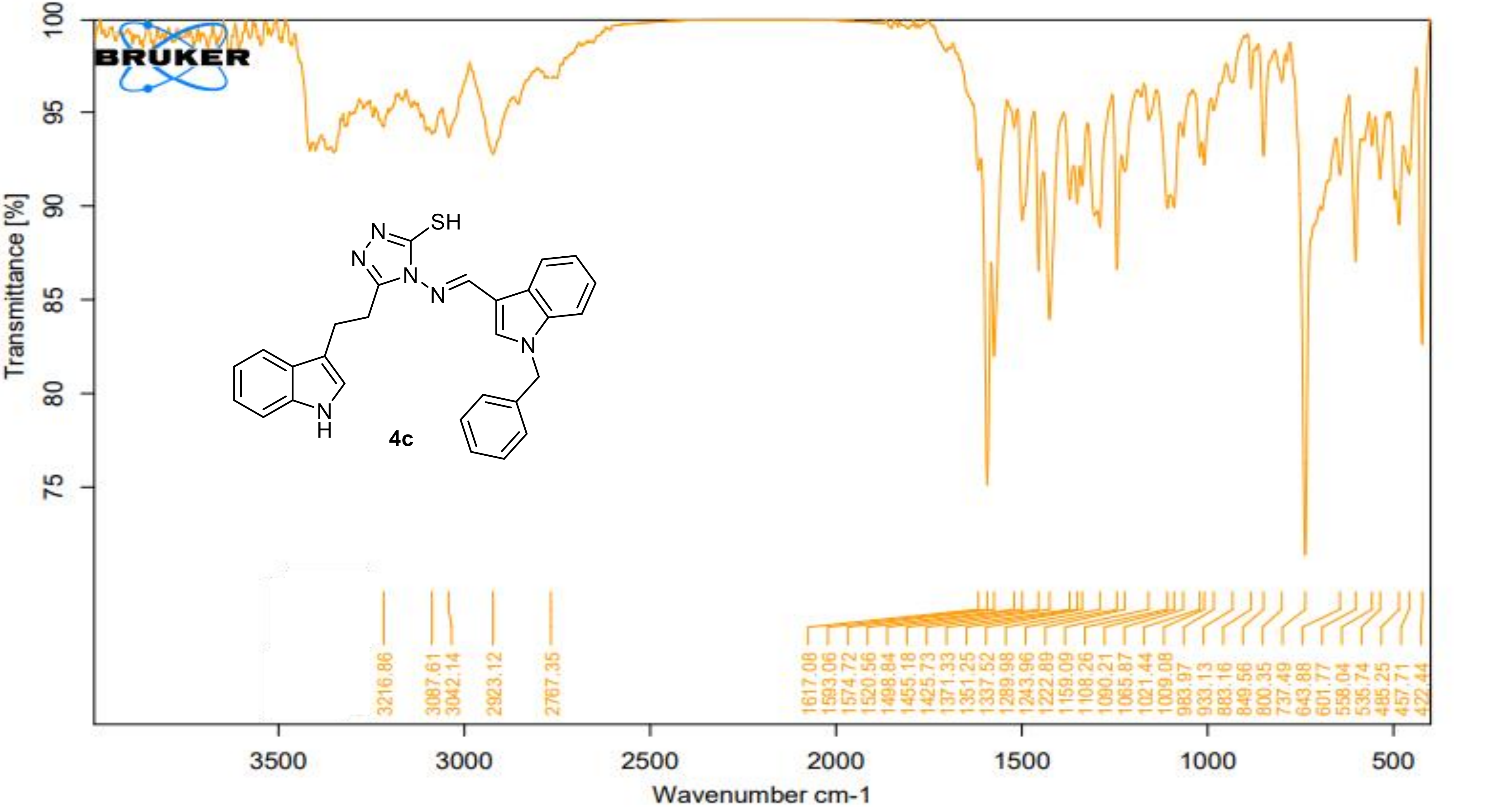
Transmittance [%]
100
95
90
85
80
75
Wavenumber cm-1
3500
3000
2500
2000
1500
1000
500
3216.86
3087.61
3042.14
2923.12
2767.35
1617.08
1593.06
1574.72
1520.56
1498.84
1455.18
1425.73
1371.33
1351.25
1337.52
1289.98
1243.96
1222.89
1159.09
1108.26
1090.21
1065.87
1021.44
1009.08
983.97
933.13
883.16
849.56
800.35
737.49
643.88
601.77
558.04
535.74
485.25
457.71
422.44
SH
N
N
N
N
N
N
H
4c

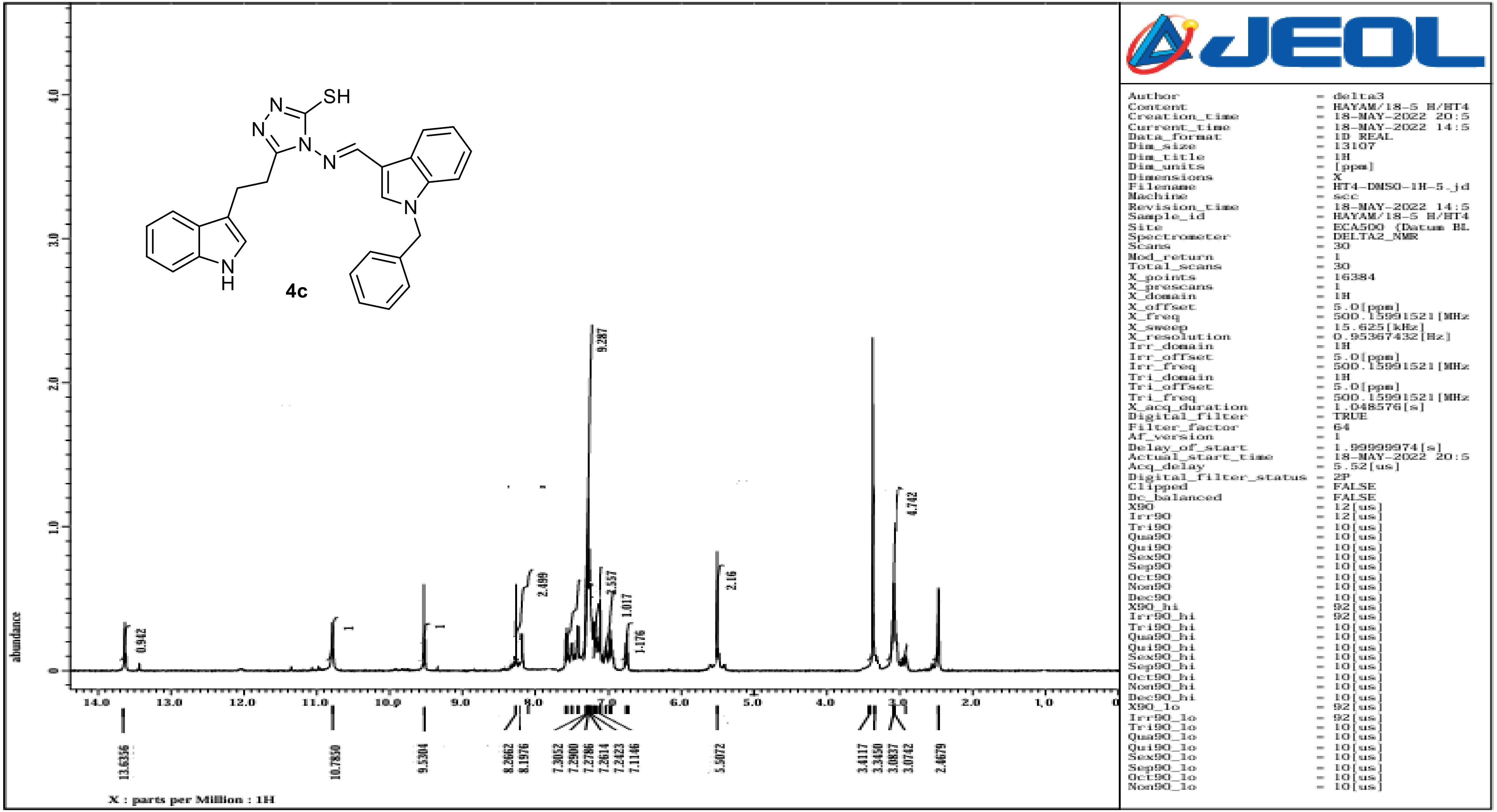

SH
N
N
N
N
N
NH
H
4c
abundance
X : parts per Million : 1H
0.942
1
1
2.499
9.287
3.557
1.017
1.176
2.16
4.742
13.6356
10.7850
9.5304
8.2662
8.1976
7.3052
7.2900
7.2786
7.2614
7.2423
7.1146
5.5072
3.4117
3.3450
3.0837
3.0742
2.4679
Author = delta3
Content = HAYAM/18-5 H/HT4
Creation_time = 18-MAY-2022 20:5
Current_time = 18-MAY-2022 14:5
Data_format = 1D REAL
Dim_size = 13107
Dim_title = 1H
Dim_units = [ppm]
Dimensions = X
Filename = HT4-DMSO-1H-5.jd
Machine = scc
Revision_time = 18-MAY-2022 14:5
Sample_id = HAYAM/18-5 H/HT4
Site = ECA500 (Datum BL
Spectrometer = DELTA2_NMR
Scans = 30
Mod_return = 1
Total_scans = 30
X_points = 16384
X_prescans = 1
X_domain = 1H
X_offset = 5.0[ppm]
X_freq = 500.15991521[MHz
X_sweep = 15.625[kHz]
X_resolution = 0.95367432[Hz]
Irr_domain = 1H
Irr_offset = 5.0[ppm]
Irr_freq = 500.15991521[MHz
Tri_domain = 1H
Tri_offset = 5.0[ppm]
Tri_freq = 500.15991521[MHz
X_acq_duration = 1.048576[s]
Digital_filter = TRUE
Filter_factor = 64
Af_version = 1
Delay_of_start = 1.99999974[s]
Actual_start_time = 18-MAY-2022 20:5
Acq_delay = 5.52[us]
Digital_filter_status = 2P
Clipped = FALSE
Dc_balanced = FALSE
X90 = 12[us]
Irr90 = 12[us]
Tri90 = 10[us]
Qua90 = 10[us]
Qui90 = 10[us]
Sex90 = 10[us]
Sep90 = 10[us]
Oct90 = 10[us]
Non90 = 10[us]
Dec90 = 10[us]
X90_hi = 92[us]
Irr90_hi = 92[us]
Tri90_hi = 10[us]
Qua90_hi = 10[us]
Qui90_hi = 10[us]
Sex90_hi = 10[us]
Sep90_hi = 10[us]
Oct90_hi = 10[us]
Non90_hi = 10[us]
Dec90_hi = 10[us]
X90_lo = 92[us]
Irr90_lo = 92[us]
Tri90_lo = 10[us]
Qua90_lo = 10[us]
Qui90_lo = 10[us]
Sex90_lo = 10[us]
Sep90_lo = 10[us]
Oct90_lo = 10[us]
Non90_lo = 10[us]

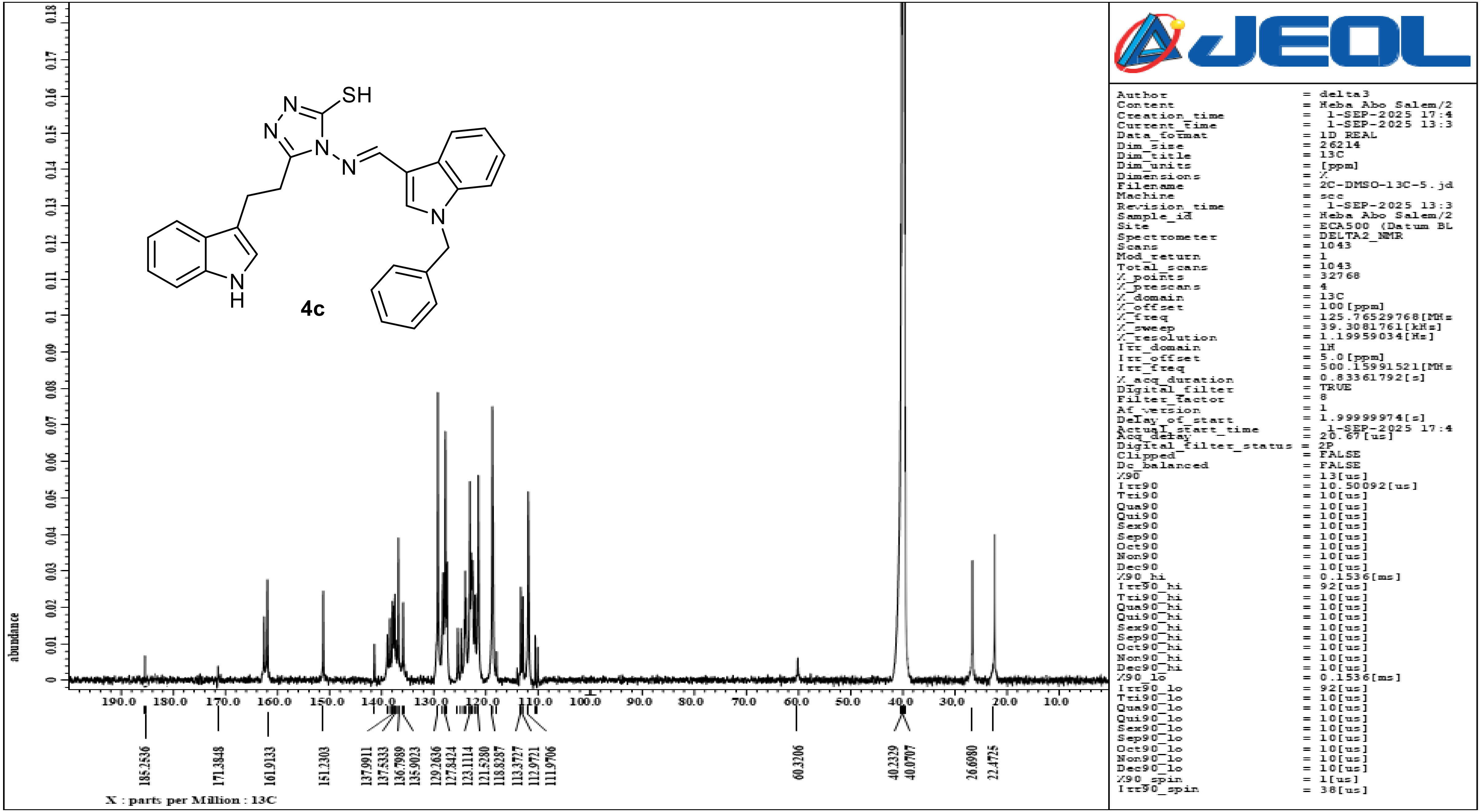

SH
N
N
N
N
N
N
N
H
4c
abundance
X : parts per Million : 13C
185.2536
171.3848
161.9133
151.2303
137.9911
137.5333
136.7989
135.9023
129.2636
127.8424
123.1114
121.5280
118.8287
113.3727
112.9721
111.9706
60.3206
40.2329
40.0707
26.6980
22.4725
JEOL
Author = delta3
Content = Heba Abo Salem/2
Creation_time = 1-SEP-2025 17:4
Current_time = 1-SEP-2025 13:3
Data_format = 1D REAL
Dim_size = 26214
Dim_title = 13C
Dim_units = [ppm]
Dimensions = X
Filename = 2C-DMSO-13C-5.jd
Machine = scc
Revision_time = 1-SEP-2025 13:3
Sample_id = Heba Abo Salem/2
Site = ECA500 (Datum BL
Spectrometer = DELTA2_NMR
Scans = 1043
Mod_return = 1
Total_scans = 1043
X_points = 32768
X_prescans = 4
X_domain = 13C
X_offset = 100[ppm]
X_freq = 125.76529768[MHz
X_sweep = 39.3081761[kHz]
X_resolution = 1.19959034[Hz]
Irr_domain = 1H
Irr_offset = 5.0[ppm]
Irr_freq = 500.15991521[MHz
X_acq_duration = 0.83361792[s]
Digital_filter = TRUE
Filter_factor = 8
Af_version = 1
Delay_of_start = 1.99999974[s]
Actual_start_time = 1-SEP-2025 17:4
Acq_delay = 20.67[us]
Digital_filter_status = 2P
Clipped = FALSE
Dc_balanced = FALSE
X90 = 13[us]
Irr90 = 10.50092[us]
Tri90 = 10[us]
Qua90 = 10[us]
Qui90 = 10[us]
Sex90 = 10[us]
Sep90 = 10[us]
Oct90 = 10[us]
Non90 = 10[us]
Dec90 = 10[us]
X90_hi = 0.1536[ms]
Irr90_hi = 92[us]
Tri90_hi = 10[us]
Qua90_hi = 10[us]
Qui90_hi = 10[us]
Sex90_hi = 10[us]
Sep90_hi = 10[us]
Oct90_hi = 10[us]
Non90_hi = 10[us]
Dec90_hi = 10[us]
X90_lo = 0.1536[ms]
Irr90_lo = 92[us]
Tri90_lo = 10[us]
Qua90_lo = 10[us]
Qui90_lo = 10[us]
Sex90_lo = 10[us]
Sep90_lo = 10[us]
Oct90_lo = 10[us]
Non90_lo = 10[us]
Dec90_lo = 10[us]
X90_spin = 1[us]
Irr90_spin = 38[us]

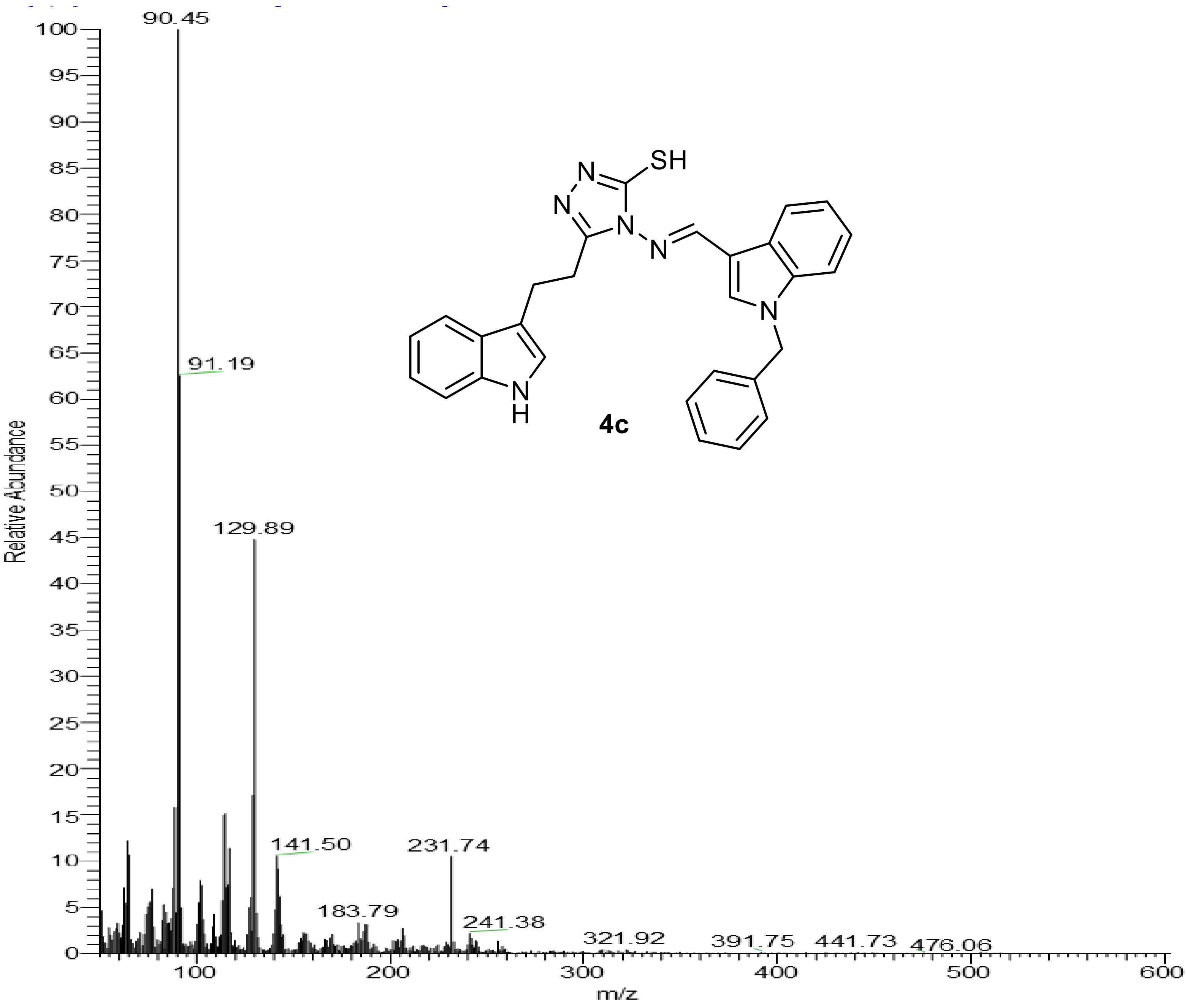
90.45
91.19
129.89
141.50
231.74
183.79
241.38
321.92
391.75
441.73
476.06
Relative Abundance
m/z
SH
N
H
4c

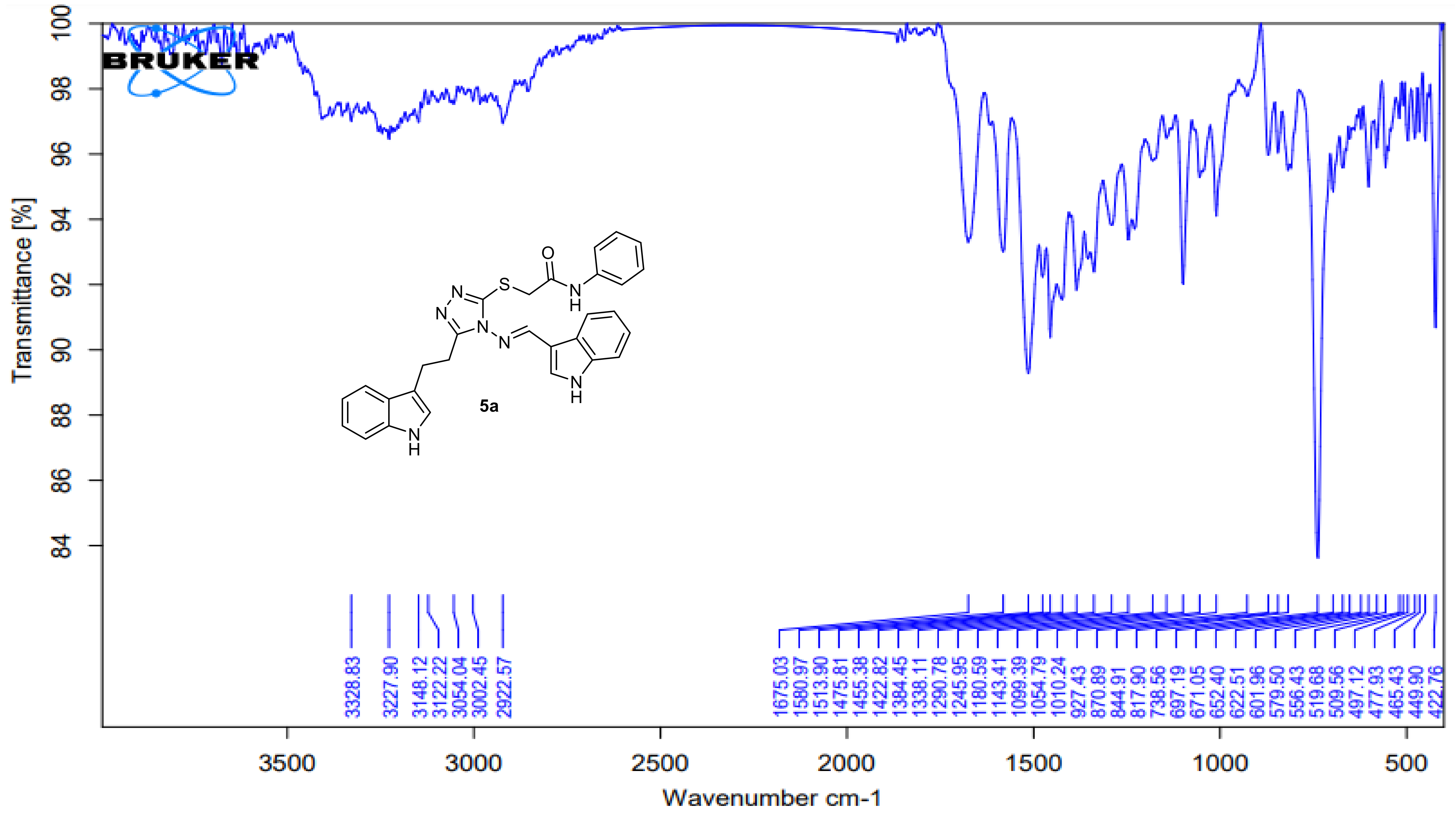

BRUKER
Transmittance [%]
100
98
96
94
92
90
88
86
84
Wavenumber cm-1
3500
3000
2500
2000
1500
1000
500
3328.83
3227.90
3148.12
3122.22
3054.04
3002.45
2922.57
1675.03
1580.97
1513.90
1475.81
1455.38
1422.82
1384.45
1338.11
1290.78
1245.95
1180.59
1143.41
1099.39
1054.79
1010.24
927.43
870.89
844.91
817.90
738.56
697.19
671.05
652.40
622.51
601.96
579.50
556.43
519.68
509.56
497.12
477.93
465.43
449.90
422.76
5a

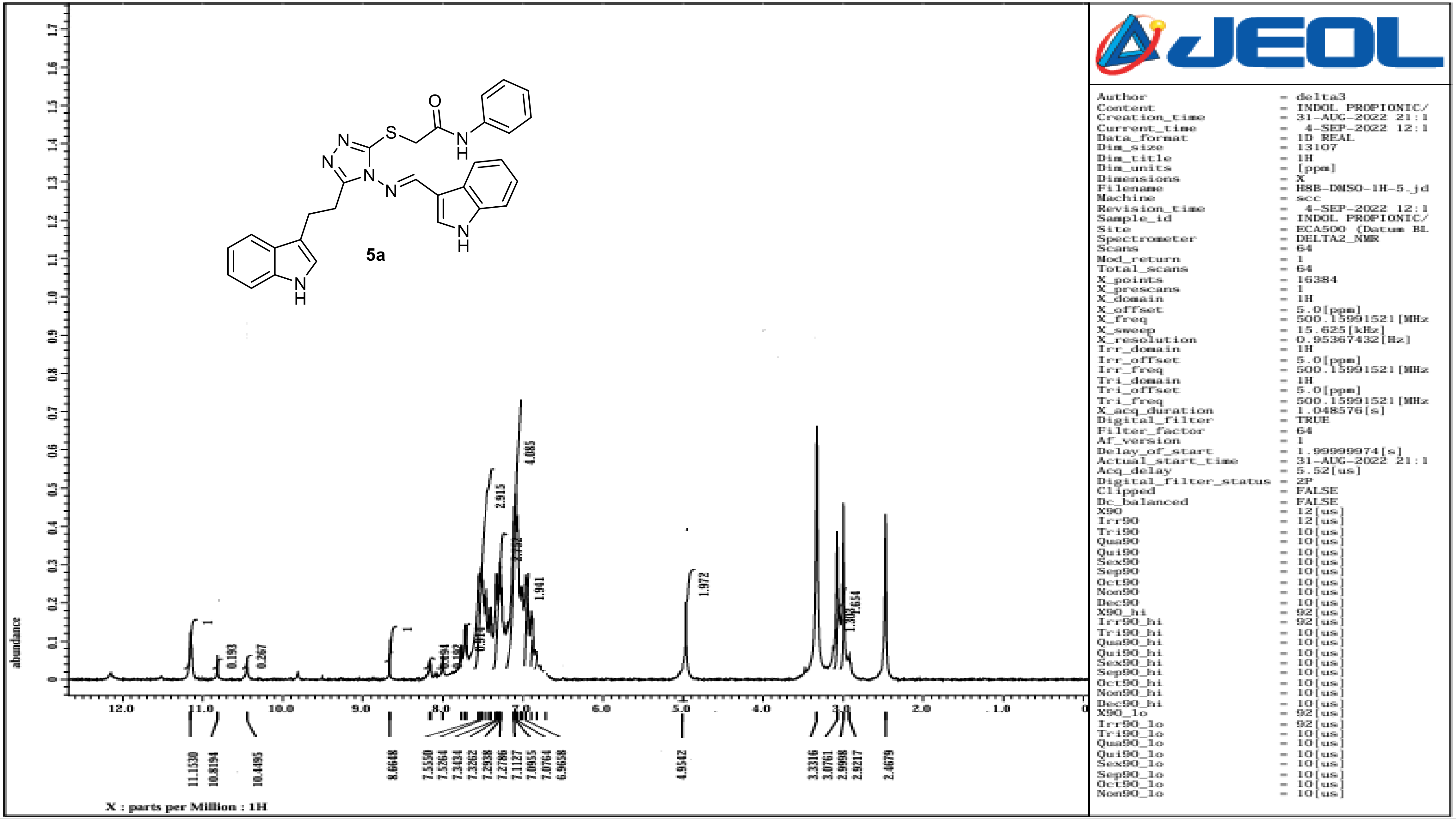

5a
abundance
X : parts per Million : 1H
12.0
11.0
10.0
9.0
8.0
7.0
6.0
5.0
4.0
3.0
2.0
1.0
11.1530
10.8194
10.4495
8.6648
7.5550
7.5264
7.3434
7.3262
7.2938
7.2786
7.1127
7.0955
7.0764
6.9658
4.9542
3.3316
3.0761
2.9998
2.9217
2.4679
4.085
2.915
1.941
1.972
Author = delta3
Content = INDOL PROPIONIC/
Creation_time = 31-AUG-2022 21:1
Current_time = 4-SEP-2022 12:1
Data_format = 1D REAL
Dim_size = 13107
Dim_title = 1H
Dim_units = [ppm]
Dimensions = X
Filename = H8B-DMSO-1H-5.jd
Machine = scc
Revision_time = 4-SEP-2022 12:1
Sample_id = INDOL PROPIONIC/
Site = ECA500 (Datum BL
Spectrometer = DELTA2_NMR
Scans = 64
Mod_return = 1
Total_scans = 64
X_points = 16384
X_prescans = 1
X_domain = 1H
X_offset = 5.0[ppm]
X_freq = 500.15991521[MHz
X_sweep = 15.625[kHz]
X_resolution = 0.95367432[Hz]
Irr_domain = 1H
Irr_offset = 5.0[ppm]
Irr_freq = 500.15991521[MHz
Tri_domain = 1H
Tri_offset = 5.0[ppm]
Tri_freq = 500.15991521[MHz
X_acq_duration = 1.048576[s]
Digital_filter = TRUE
Filter_factor = 64
Af_version = 1
Delay_of_start = 1.99999974[s]
Actual_start_time = 31-AUG-2022 21:1
Acq_delay = 5.52[us]
Digital_filter_status = 2P
Clipped = FALSE
Dc_balanced = FALSE
X90 = 12[us]
Irr90 = 12[us]
Tri90 = 10[us]
Qua90 = 10[us]
Qui90 = 10[us]
Sex90 = 10[us]
Sep90 = 10[us]
Oct90 = 10[us]
Non90 = 10[us]
Dec90 = 10[us]
X90_hi = 92[us]
Irr90_hi = 92[us]
Tri90_hi = 10[us]
Qua90_hi = 10[us]
Qui90_hi = 10[us]
Sex90_hi = 10[us]
Sep90_hi = 10[us]
Oct90_hi = 10[us]
Non90_hi = 10[us]
Dec90_hi = 10[us]
X90_lo = 92[us]
Irr90_lo = 92[us]
Tri90_lo = 10[us]
Qua90_lo = 10[us]
Qui90_lo = 10[us]
Sex90_lo = 10[us]
Sep90_lo = 10[us]
Oct90_lo = 10[us]
Non90_lo = 10[us]

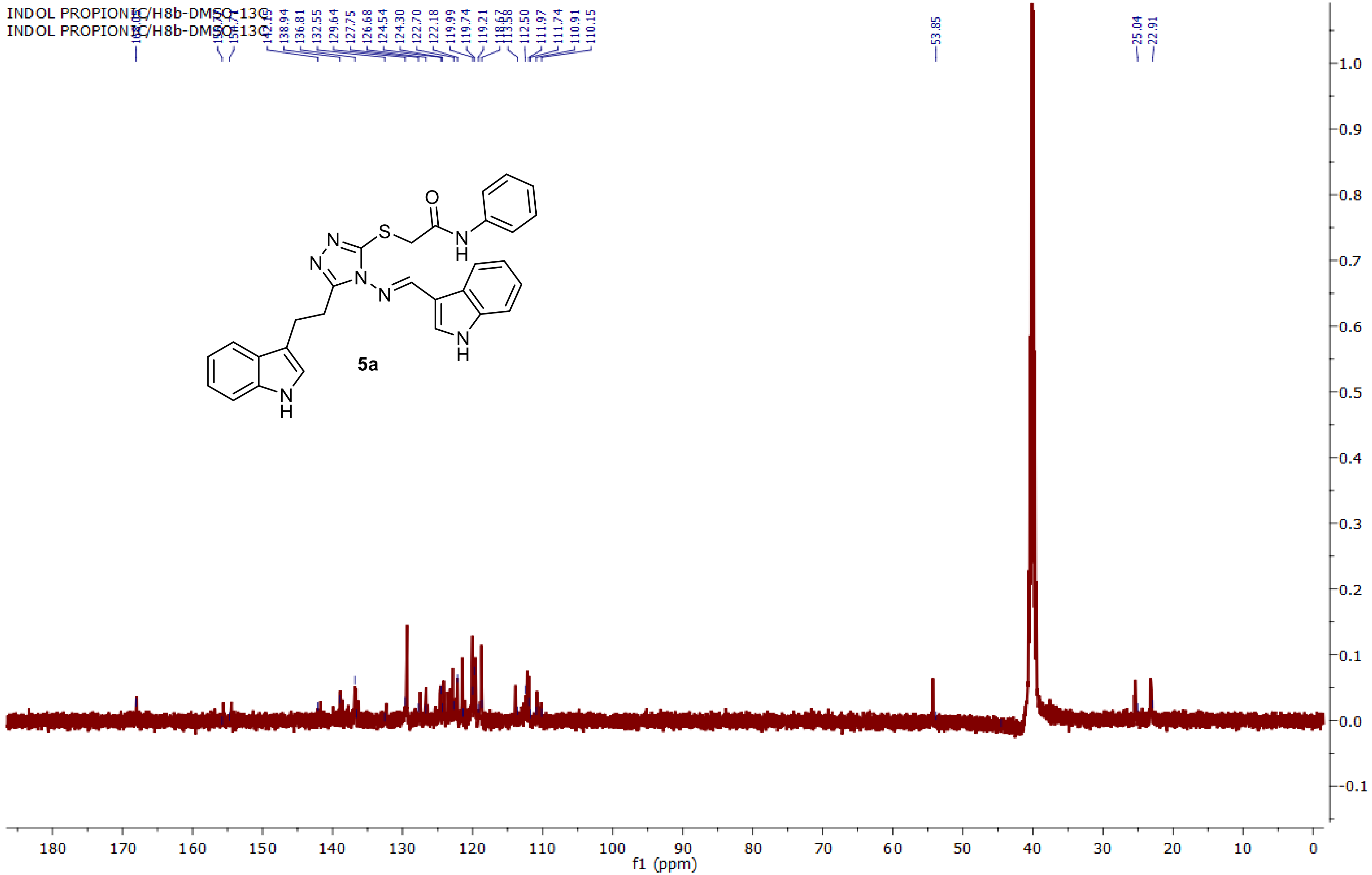
INDOL PROPIONIC/H8b-DMSO-13C
138.94
136.81
132.55
129.64
127.75
126.68
124.54
124.30
122.70
122.18
119.99
119.74
119.21
118.67
113.58
112.50
111.97
111.74
110.91
110.15
53.85
25.04
22.91
5a
f1 (ppm)

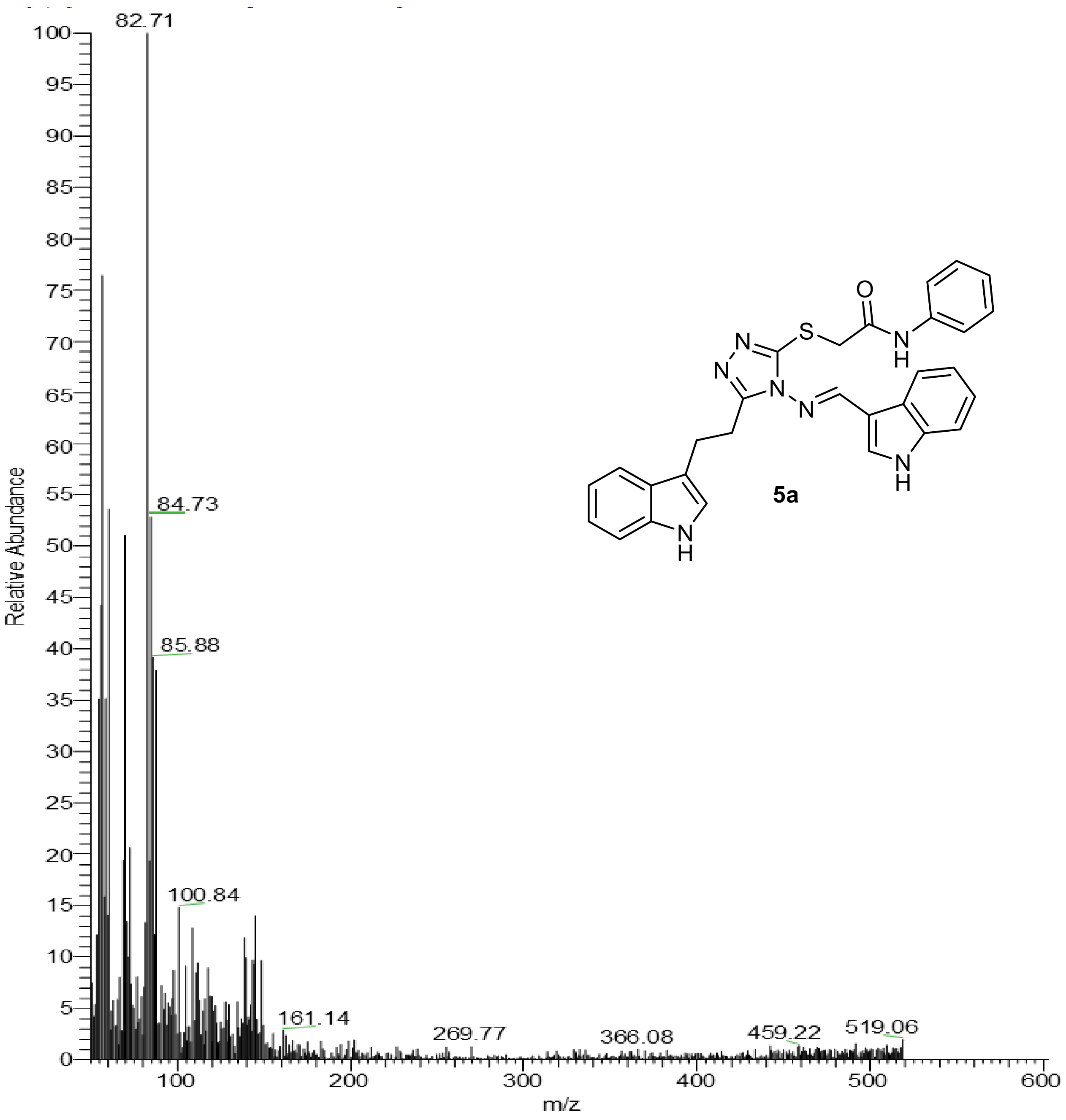
82.71
84.73
85.88
100.84
161.14
269.77
366.08
459.22
519.06
Relative Abundance
m/z
5a

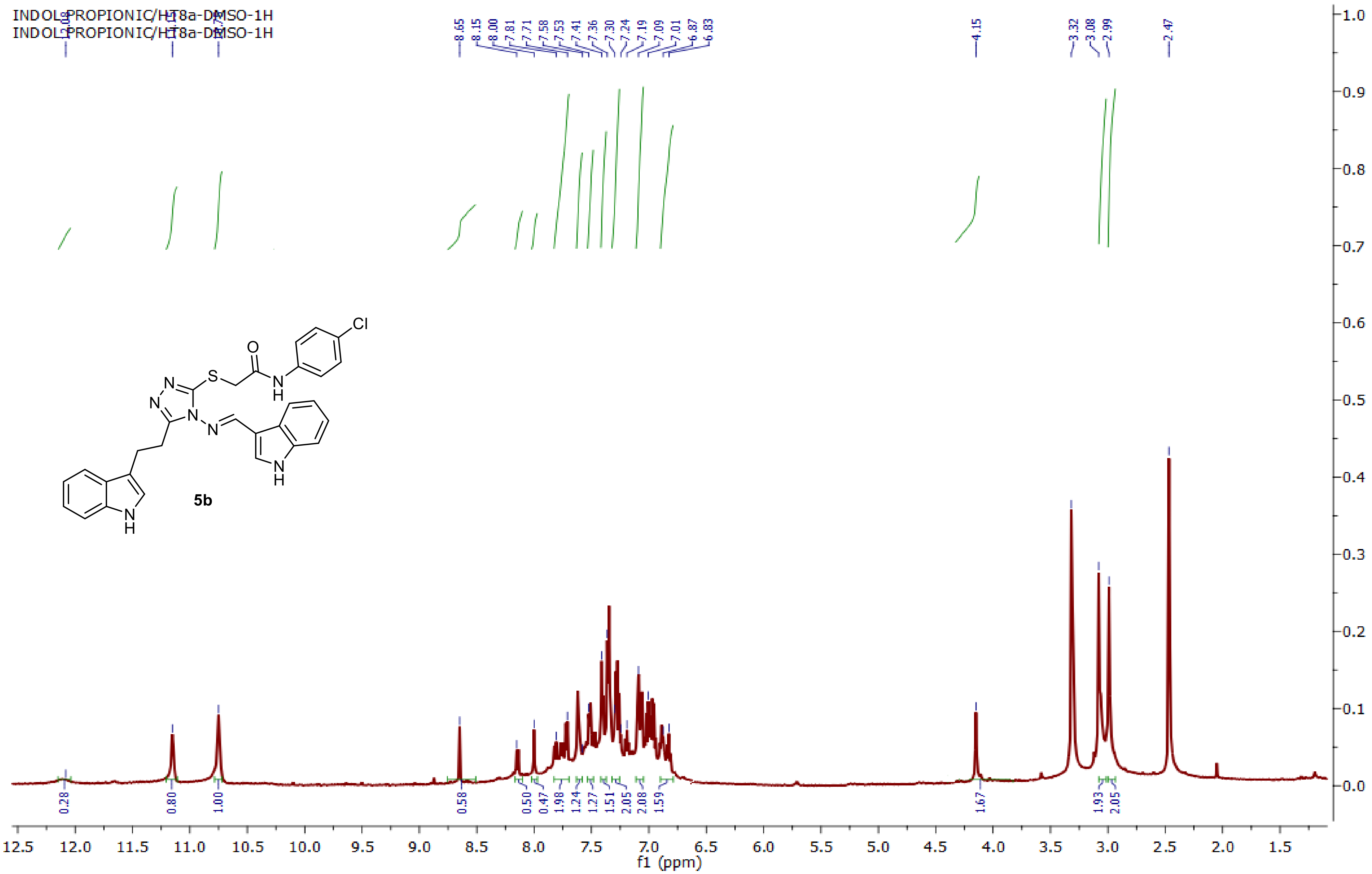
INDOL PROPIONIC/HT8a-DMSO-1H
INDOL PROPIONIC/HT8a-DMSO-1H
8.65
8.15
8.00
7.81
7.71
7.58
7.53
7.41
7.36
7.30
7.24
7.19
7.09
7.01
6.87
6.83
4.15
3.32
3.08
2.99
2.47
Cl
O
S
N
H
N
N
N
N
N
H
N
H
5b
0.28
0.80
1.00
0.58
0.50
0.47
1.98
1.24
1.27
1.51
2.05
2.08
1.59
1.67
1.93
2.05
f1 (ppm)

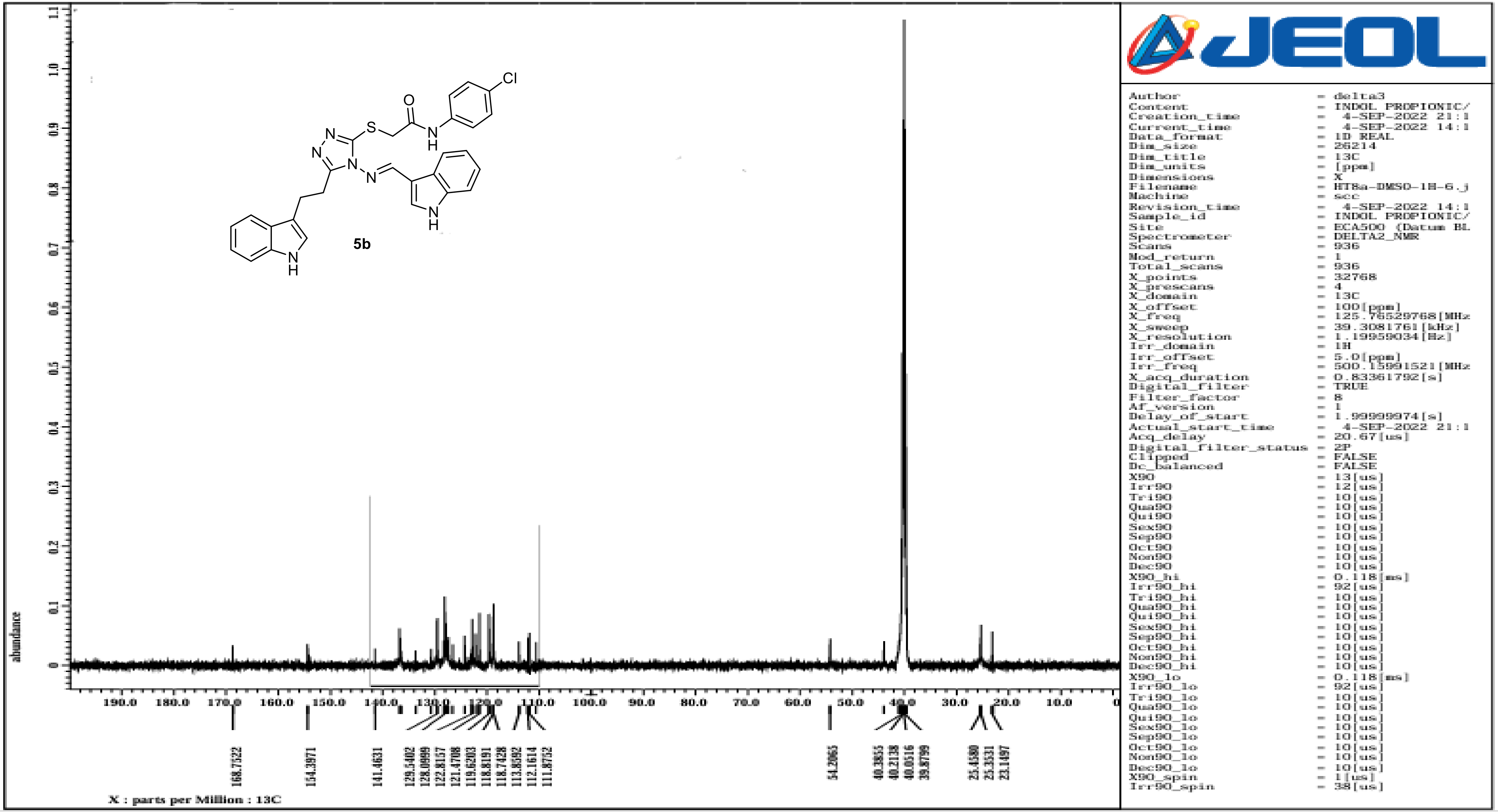

Cl
O
S
N
N
N
H
N
N
N
N
H
5b
N
H
abundance
X : parts per Million : 13C
168.7522
154.3971
141.4631
129.5402
128.0999
122.8157
121.4708
119.6203
118.8191
118.7428
113.8382
112.1614
111.8752
54.2065
40.3855
40.2138
40.0516
39.8799
25.4580
25.3531
23.1497
JEOL
Author = delta3
Content = INDOL PROPIONIC/
Creation_time = 4-SEP-2022 21:1
Current_time = 4-SEP-2022 14:1
Data_format = 1D REAL
Dim_size = 26214
Dim_title = 13C
Dim_units = [ppm]
Dimensions = X
Filename = HT8a-DMSO-1H-6.j
Machine = scc
Revision_time = 4-SEP-2022 14:1
Sample_id = INDOL PROPIONIC/
Site = ECA500 (Datum BL
Spectrometer = DELTA2_NMR
Scans = 936
Mod_return = 1
Total_scans = 936
X_points = 32768
X_prescans = 4
X_domain = 13C
X_offset = 100[ppm]
X_freq = 125.76529768[MHz
X_sweep = 39.3081761[kHz]
X_resolution = 1.19959034[Hz]
Irr_domain = 1H
Irr_offset = 5.0[ppm]
Irr_freq = 500.15991521[MHz
X_acq_duration = 0.83361792[s]
Digital_filter = TRUE
Filter_factor = 8
Af_version = 1
Delay_of_start = 1.99999974[s]
Actual_start_time = 4-SEP-2022 21:1
Acq_delay = 20.67[us]
Digital_filter_status = 2P
Clipped = FALSE
Dc_balanced = FALSE
X90 = 13[us]
Irr90 = 12[us]
Tri90 = 10[us]
Qua90 = 10[us]
Qui90 = 10[us]
Sex90 = 10[us]
Sep90 = 10[us]
Oct90 = 10[us]
Non90 = 10[us]
Dec90 = 10[us]
X90_hi = 0.118[ms]
Irr90_hi = 92[us]
Tri90_hi = 10[us]
Qua90_hi = 10[us]
Qui90_hi = 10[us]
Sex90_hi = 10[us]
Sep90_hi = 10[us]
Oct90_hi = 10[us]
Non90_hi = 10[us]
Dec90_hi = 10[us]
X90_lo = 0.118[ms]
Irr90_lo = 92[us]
Tri90_lo = 10[us]
Qua90_lo = 10[us]
Qui90_lo = 10[us]
Sex90_lo = 10[us]
Sep90_lo = 10[us]
Oct90_lo = 10[us]
Non90_lo = 10[us]
Dec90_lo = 10[us]
X90_spin = 1[us]
Irr90_spin = 38[us]

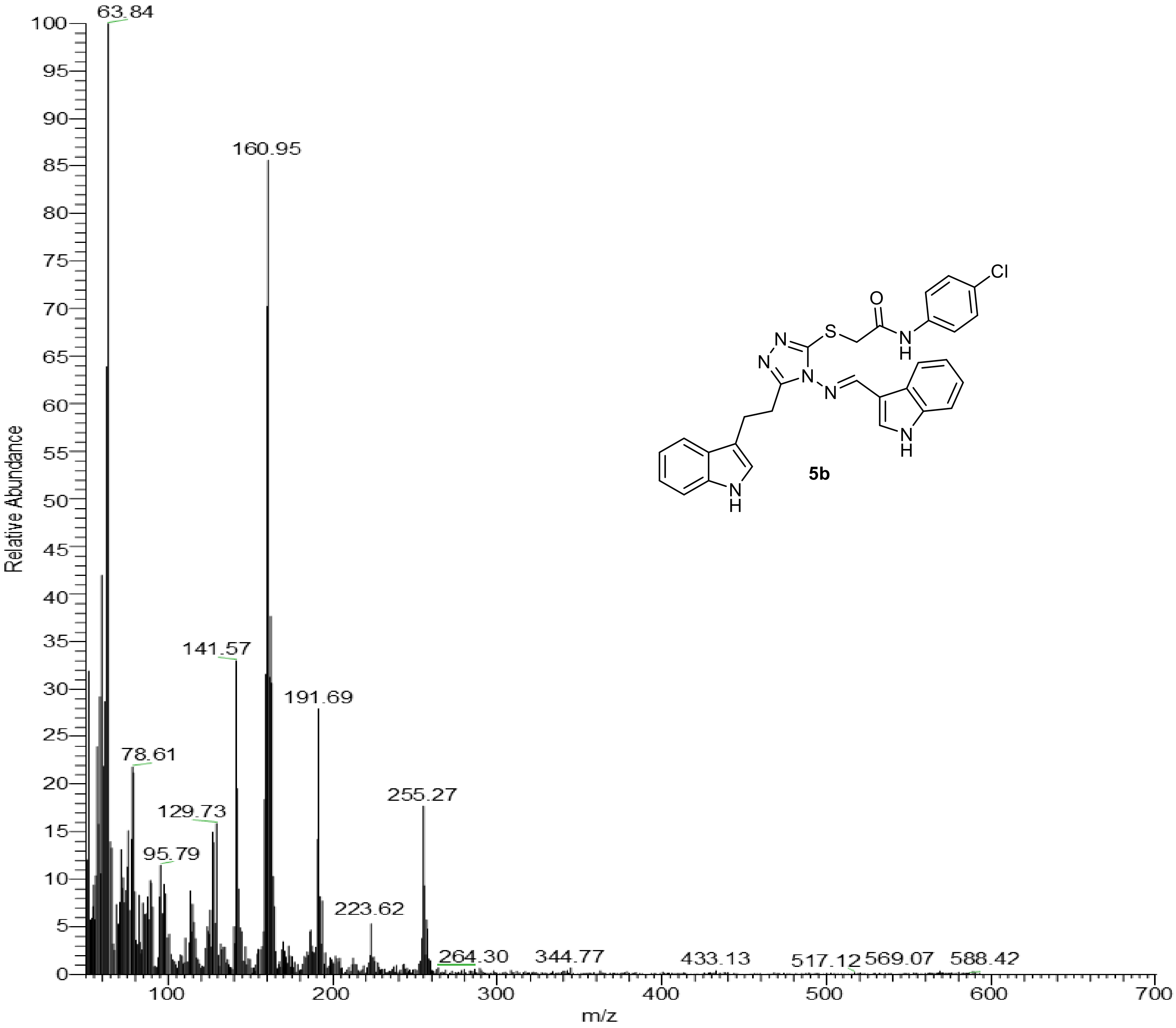

63.84
160.95
141.57
191.69
78.61
129.73
95.79
255.27
223.62
264.30
344.77
433.13
517.12
569.07
588.42
Relative Abundance
m/z
5b

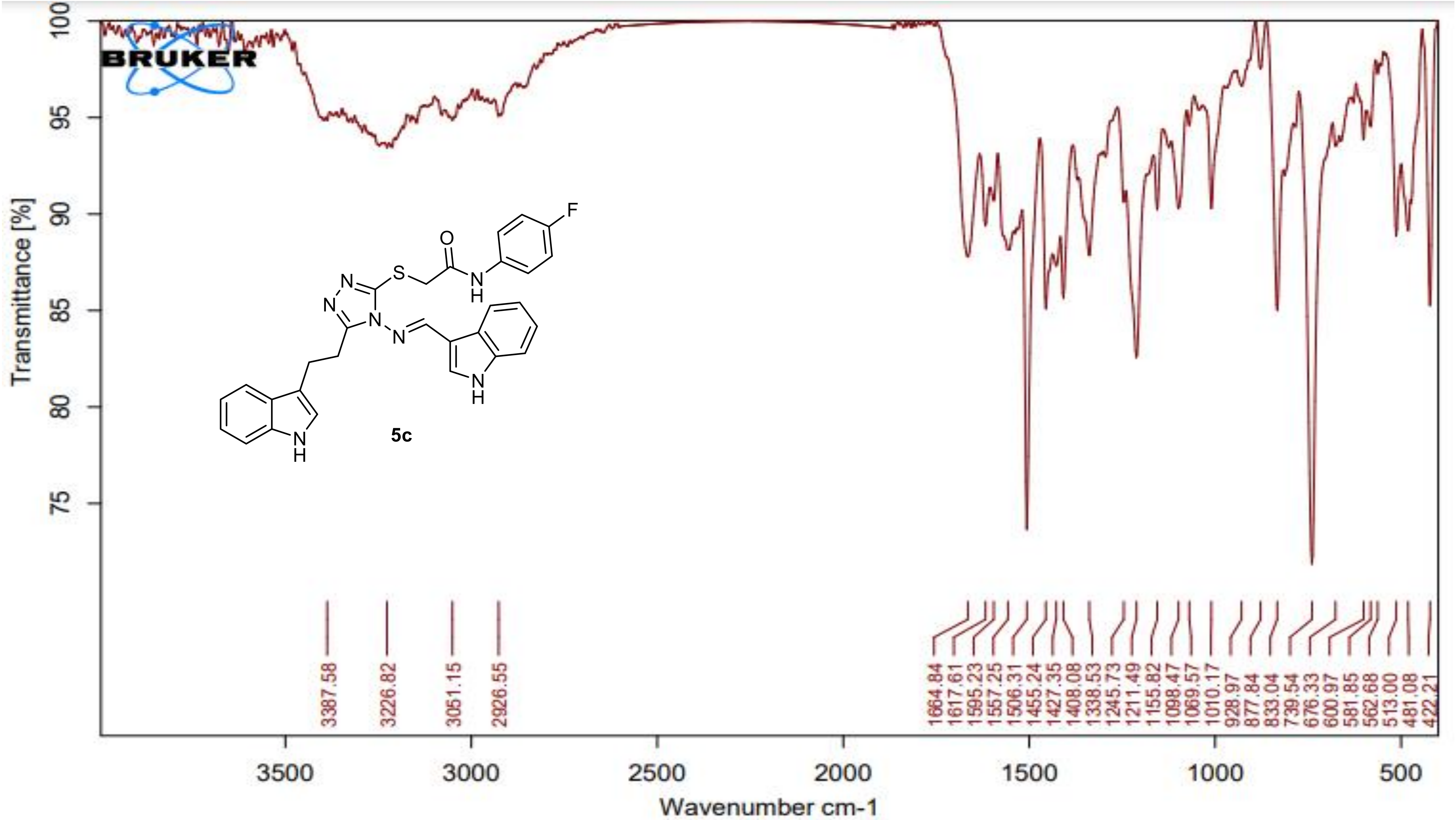
BRUKER
Transmittance [%]
100
95
90
85
80
75
Wavenumber cm-1
3500
3000
2500
2000
1500
1000
500
5c
3387.58
3226.82
3051.15
2926.55
1664.84
1617.61
1595.23
1557.25
1506.31
1455.24
1427.35
1408.08
1338.53
1245.73
1211.49
1155.82
1098.47
1069.57
1010.17
928.97
877.84
833.04
739.54
676.33
600.97
581.85
562.68
513.00
481.08
422.21

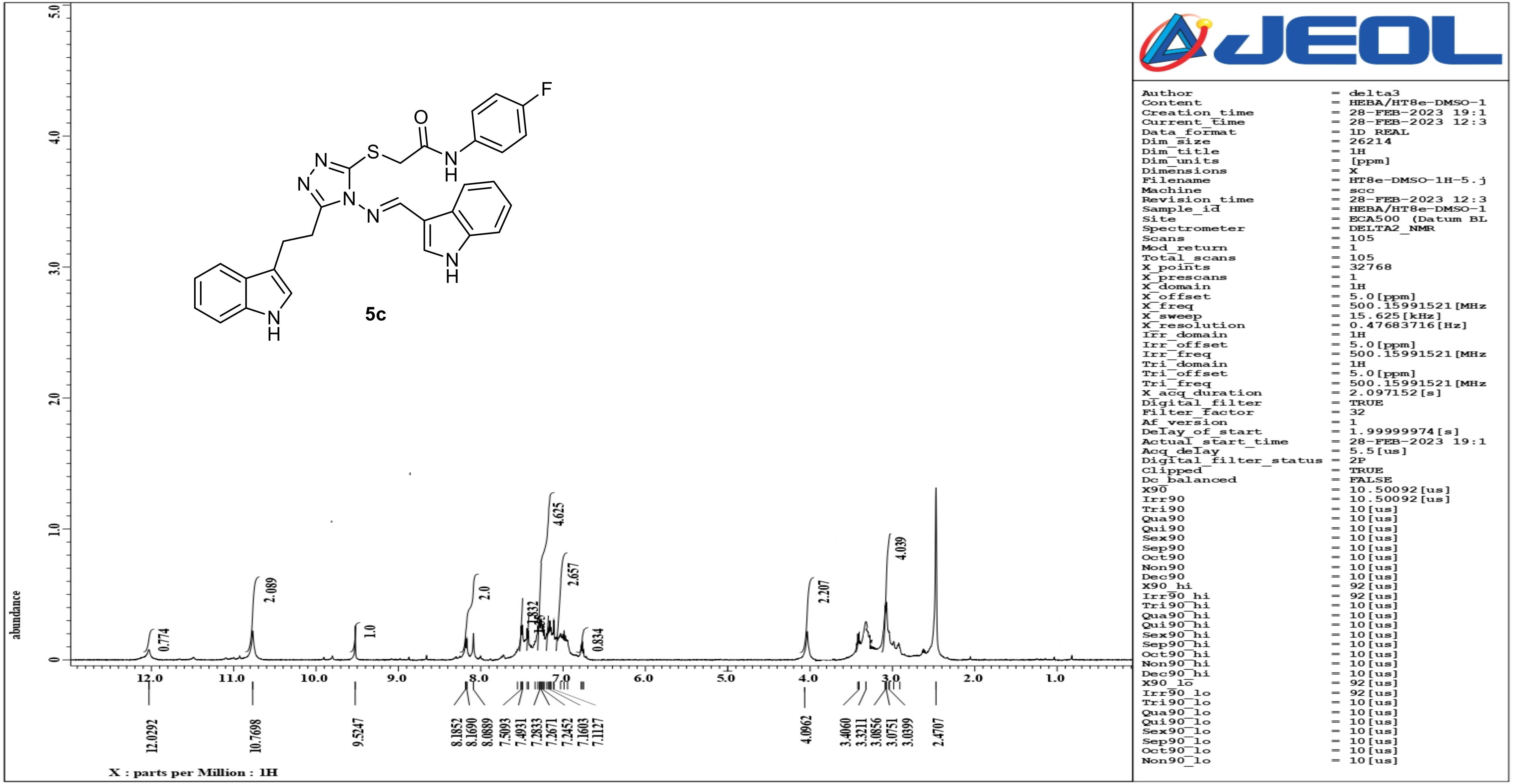
5c
abundance
X : parts per Million : 1H
12.0292
10.7698
9.5247
8.1852
8.1690
8.0889
7.5093
7.4931
7.2833
7.2671
7.2452
7.1603
7.1127
4.0962
3.4060
3.3211
3.0856
3.0751
3.0399
2.4707
0.774
2.089
1.0
2.0
1.832
4.625
2.657
0.834
2.207
4.039
JEOL
Author = delta3
Content = HEBA/HT8e-DMSO-1
Creation_time = 28-FEB-2023 19:1
Current_time = 28-FEB-2023 12:3
Data_format = 1D REAL
Dim_size = 26214
Dim_title = 1H
Dim_units = [ppm]
Dimensions = X
Filename = HT8e-DMSO-1H-5.j
Machine = scc
Revision_time = 28-FEB-2023 12:3
Sample_id = HEBA/HT8e-DMSO-1
Site = ECA500 (Datum BL
Spectrometer = DELTA2_NMR
Scans = 105
Mod_return = 1
Total_scans = 105
X_points = 32768
X_prescans = 1
X_domain = 1H
X_offset = 5.0[ppm]
X_freq = 500.15991521[MHz
X_sweep = 15.625[kHz]
X_resolution = 0.47683716[Hz]
Irr_domain = 1H
Irr_offset = 5.0[ppm]
Irr_freq = 500.15991521[MHz
Tri_domain = 1H
Tri_offset = 5.0[ppm]
Tri_freq = 500.15991521[MHz
X_acq_duration = 2.097152[s]
Digital_filter = TRUE
Filter_factor = 32
Af_version = 1
Delay_of_start = 1.99999974[s]
Actual_start_time = 28-FEB-2023 19:1
Acq_delay = 5.5[us]
Digital_filter_status = 2P
Clipped = TRUE
Dc_balanced = FALSE
X90 = 10.50092[us]
Irr90 = 10.50092[us]
Tri90 = 10[us]
Qua90 = 10[us]
Qui90 = 10[us]
Sex90 = 10[us]
Sep90 = 10[us]
Oct90 = 10[us]
Non90 = 10[us]
Dec90 = 10[us]
X90_hi = 92[us]
Irr90_hi = 92[us]
Tri90_hi = 10[us]
Qua90_hi = 10[us]
Qui90_hi = 10[us]
Sex90_hi = 10[us]
Sep90_hi = 10[us]
Oct90_hi = 10[us]
Non90_hi = 10[us]
Dec90_hi = 10[us]
X90_lo = 92[us]
Irr90_lo = 92[us]
Tri90_lo = 10[us]
Qua90_lo = 10[us]
Qui90_lo = 10[us]
Sex90_lo = 10[us]
Sep90_lo = 10[us]
Oct90_lo = 10[us]
Non90_lo = 10[us]

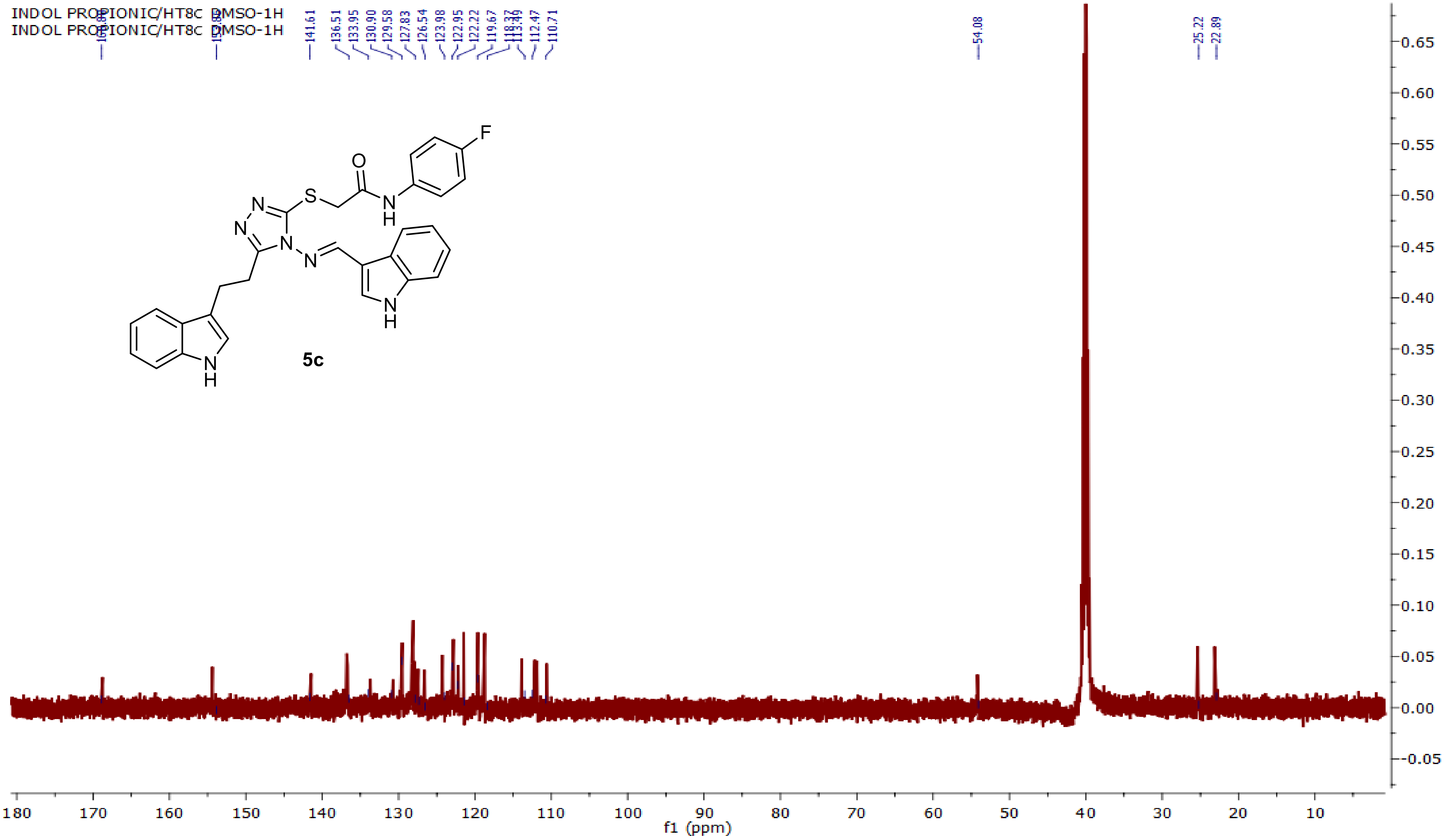
INDOL PROPIONIC/HT8c DMSO-1H
141.61
136.51
133.95
130.90
129.58
127.83
126.54
123.98
122.95
122.22
119.67
112.47
110.71
54.08
25.22
22.89
F
O
S
N
H
N
N
N
N
N
H
N
H
5c
0.65
0.60
0.55
0.50
0.45
0.40
0.35
0.30
0.25
0.20
0.15
0.10
0.05
0.00
-0.05
180
170
160
150
140
130
120
110
100
90
80
70
60
50
40
30
20
10
f1 (ppm)

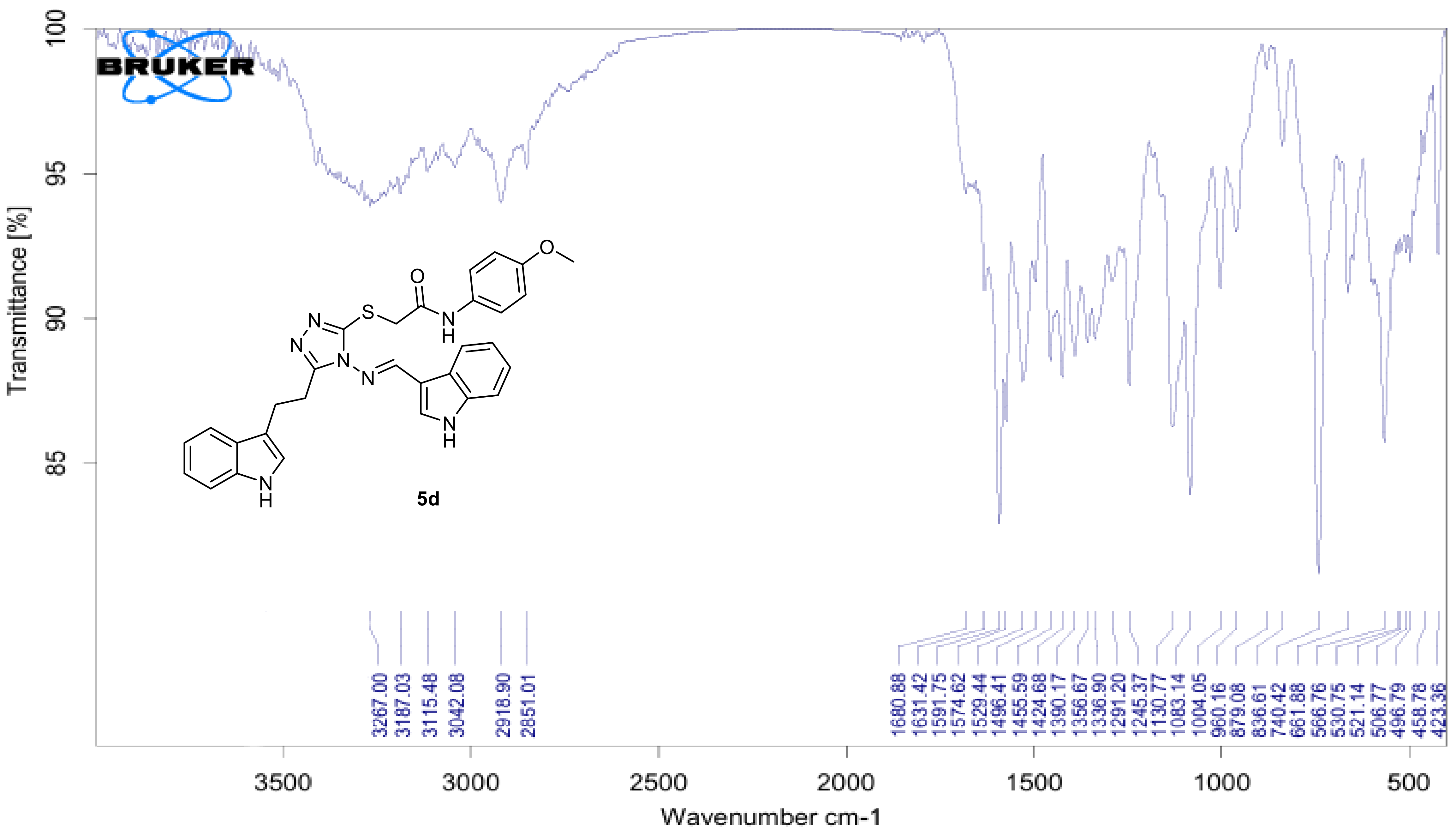
BRUKER
Transmittance [%]
100
95
90
85
Wavenumber cm-1
3500
3000
2500
2000
1500
1000
500
3267.00
3187.03
3115.48
3042.08
2918.90
2851.01
1680.88
1631.42
1591.75
1574.62
1529.44
1496.41
1455.59
1424.68
1390.17
1356.67
1336.90
1291.20
1245.37
1130.77
1083.14
1004.05
960.16
879.08
836.61
740.42
661.88
566.76
530.75
521.14
506.77
496.79
458.78
423.36
5d

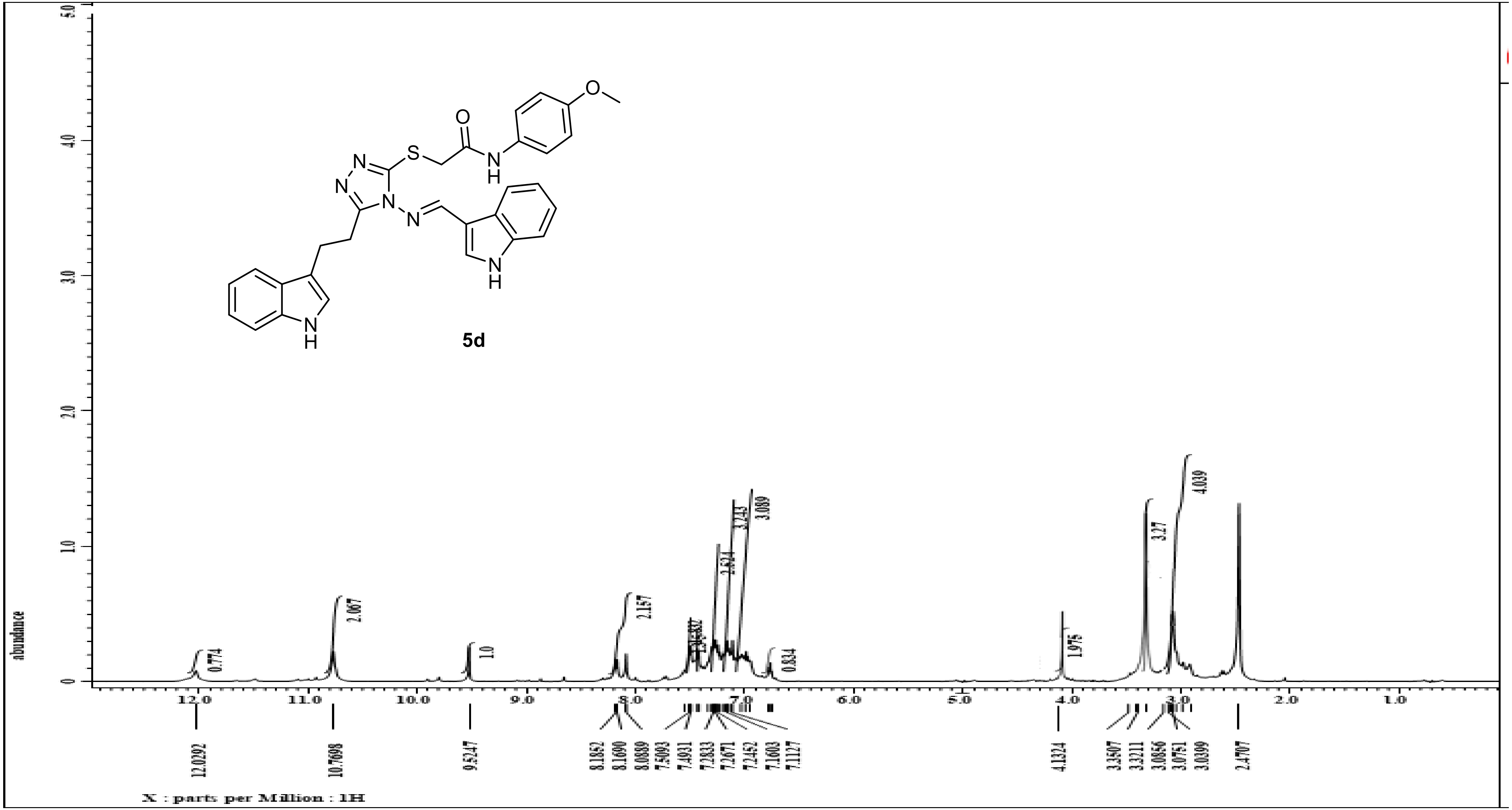
5d
abundance
X : parts per Million : 1H

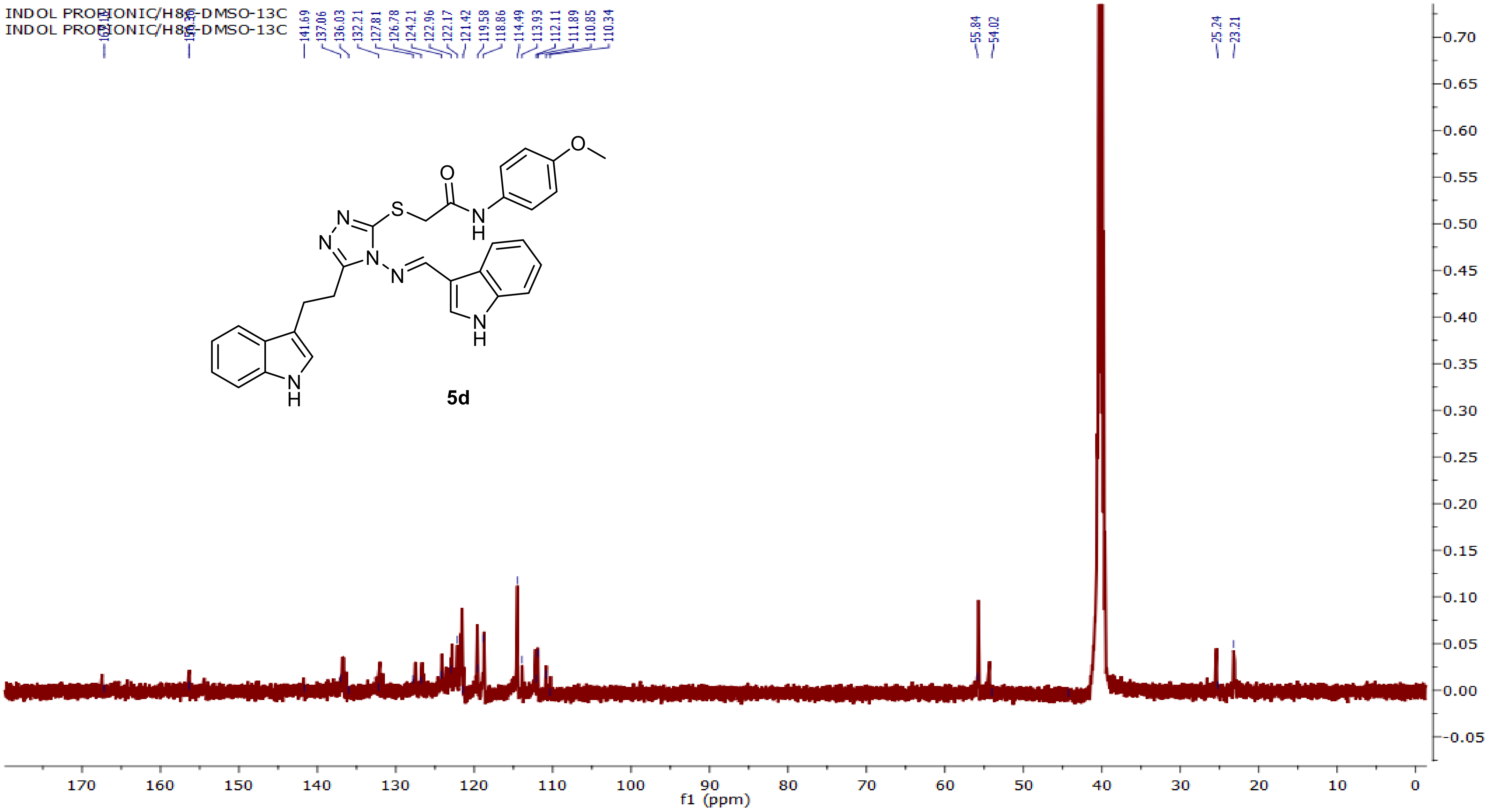
INDOL PROPIONIC/H8C-DMSO-13C
141.69
137.06
136.03
132.21
127.81
126.78
124.21
122.96
122.17
121.42
119.58
118.86
114.49
113.93
112.11
111.89
110.85
110.34
55.84
54.02
25.24
23.21
5d
f1 (ppm)

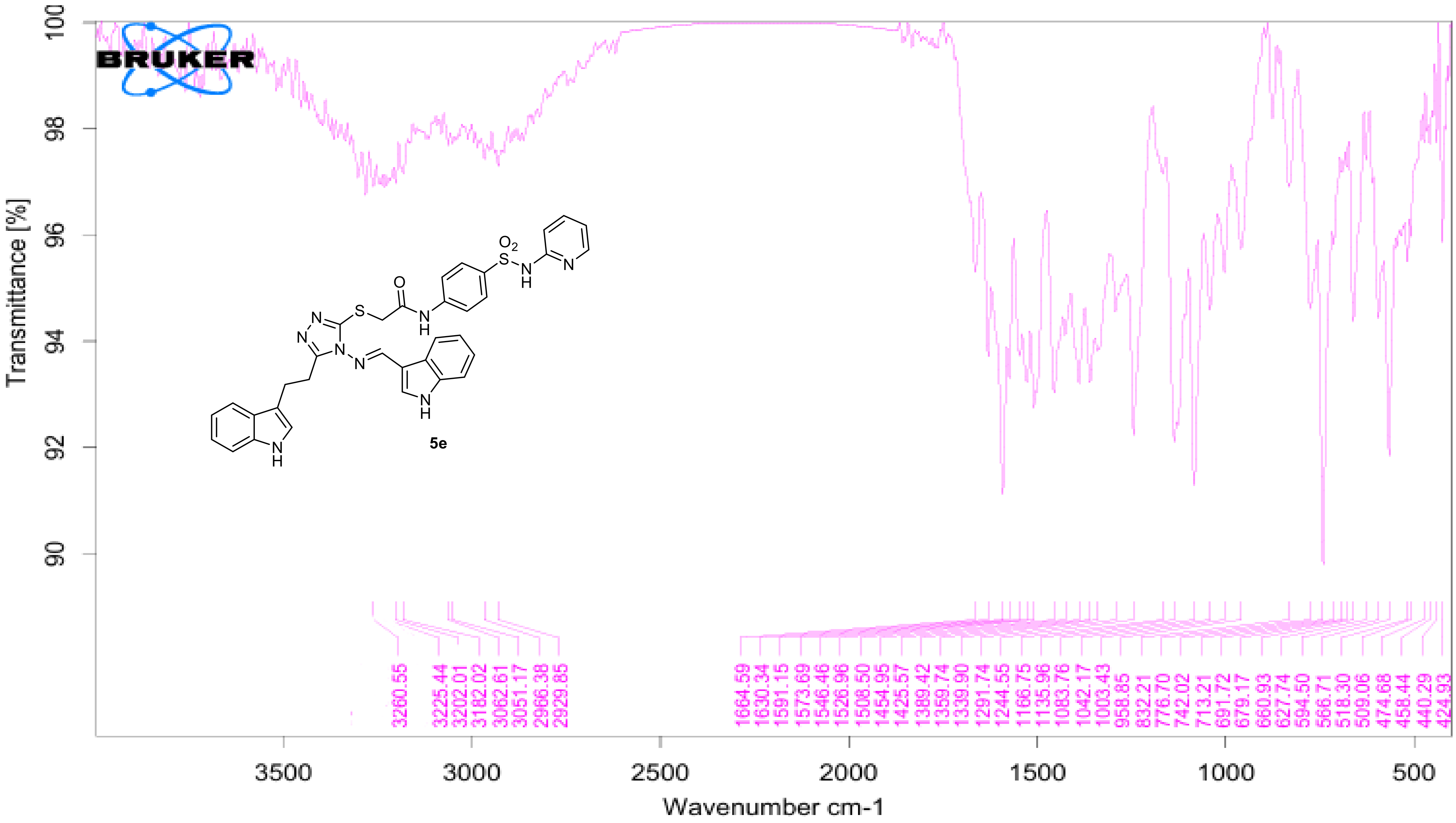
BRUKER
Transmittance [%]
Wavenumber cm-1
100
98
96
94
92
90
3500
3000
2500
2000
1500
1000
500
3260.55
3225.44
3202.01
3182.02
3062.61
3051.17
2966.38
2929.85
1664.59
1630.34
1591.15
1573.69
1546.46
1526.96
1508.50
1454.95
1425.57
1389.42
1359.74
1339.90
1291.74
1244.55
1166.75
1135.96
1083.76
1042.17
1003.43
958.85
832.21
776.70
742.02
713.21
691.72
679.17
660.93
627.74
594.50
566.71
518.30
509.06
474.68
458.44
440.29
424.93
5e

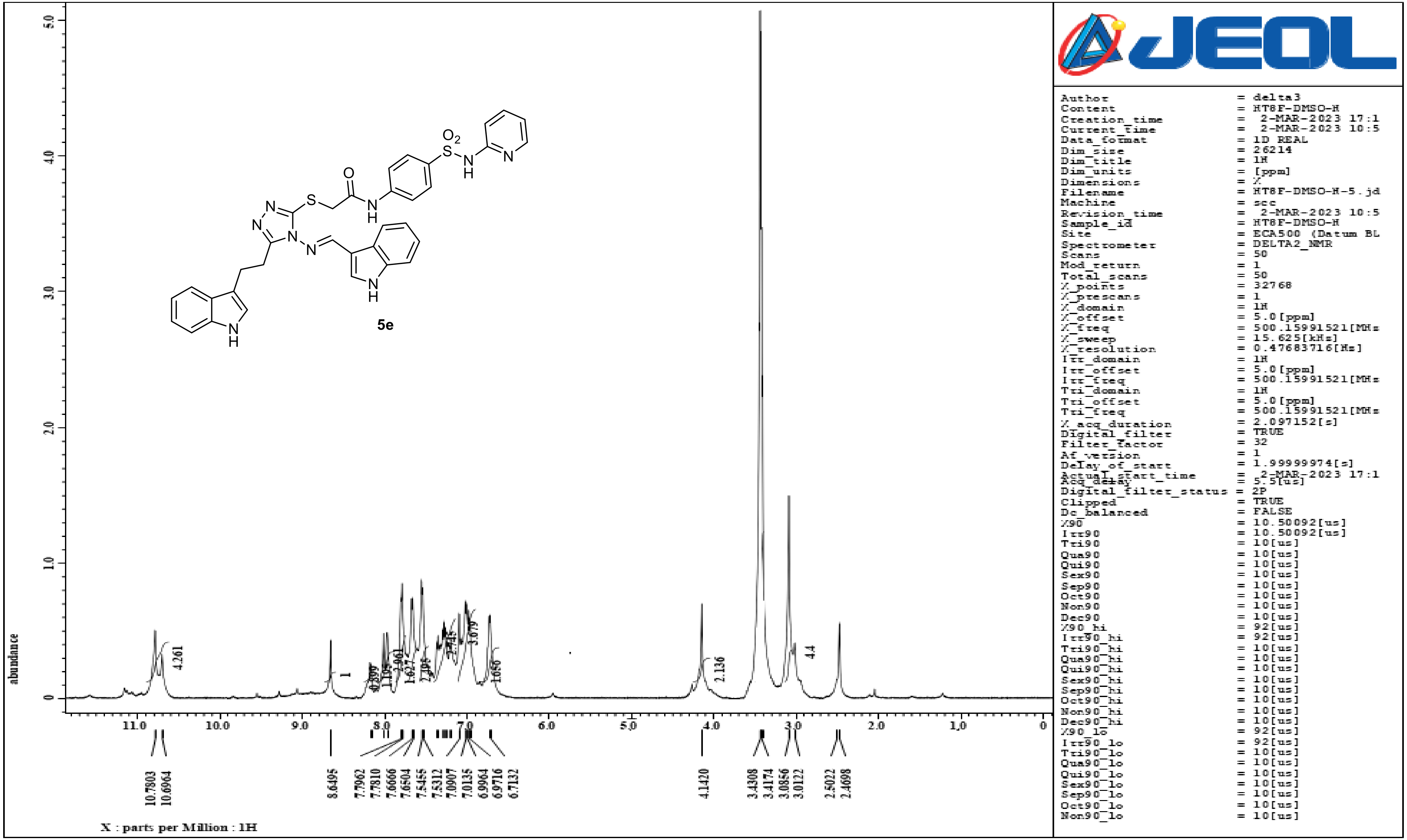

5e
abundnace
X : parts per Million : 1H
10.7803
10.6964
8.6495
7.7962
7.7810
7.6666
7.6504
7.5455
7.5312
7.0907
7.0135
6.9964
6.9716
6.7132
4.1420
3.4308
3.4174
3.0856
3.0122
2.5022
2.4698
4.261
2.136
4.4
Author = delta3
Content = HT8F-DMSO-H
Creation_time = 2-MAR-2023 17:1
Current_time = 2-MAR-2023 10:5
Data_format = 1D REAL
Dim_size = 26214
Dim_title = 1H
Dim_units = [ppm]
Dimensions = X
Filename = HT8F-DMSO-H-5.jd
Machine = scc
Revision_time = 2-MAR-2023 10:5
Sample_id = HT8F-DMSO-H
Site = ECA500 (Datum BL
Spectrometer = DELTA2_NMR
Scans = 50
Mod_return = 1
Total_scans = 50
X_points = 32768
X_prescans = 1
X_domain = 1H
X_offset = 5.0[ppm]
X_freq = 500.15991521[MHz
X_sweep = 15.625[kHz]
X_resolution = 0.47683716[Hz]
Irr_domain = 1H
Irr_offset = 5.0[ppm]
Irr_freq = 500.15991521[MHz
Tri_domain = 1H
Tri_offset = 5.0[ppm]
Tri_freq = 500.15991521[MHz
X_acq_duration = 2.097152[s]
Digital_filter = TRUE
Filter_factor = 32
Af_version = 1
Delay_of_start = 1.99999974[s]
Actual_start_time = 2-MAR-2023 17:1
Acq_delay = 5.5[us]
Digital_filter_status = 2P
Clipped = TRUE
Dc_balanced = FALSE
X90 = 10.50092[us]
Irr90 = 10.50092[us]
Tri90 = 10[us]
Qua90 = 10[us]
Qui90 = 10[us]
Sex90 = 10[us]
Sep90 = 10[us]
Oct90 = 10[us]
Non90 = 10[us]
Dec90 = 10[us]
X90_hi = 92[us]
Irr90_hi = 92[us]
Tri90_hi = 10[us]
Qua90_hi = 10[us]
Qui90_hi = 10[us]
Sex90_hi = 10[us]
Sep90_hi = 10[us]
Oct90_hi = 10[us]
Non90_hi = 10[us]
Dec90_hi = 10[us]
X90_lo = 92[us]
Irr90_lo = 92[us]
Tri90_lo = 10[us]
Qua90_lo = 10[us]
Qui90_lo = 10[us]
Sex90_lo = 10[us]
Sep90_lo = 10[us]
Oct90_lo = 10[us]
Non90_lo = 10[us]

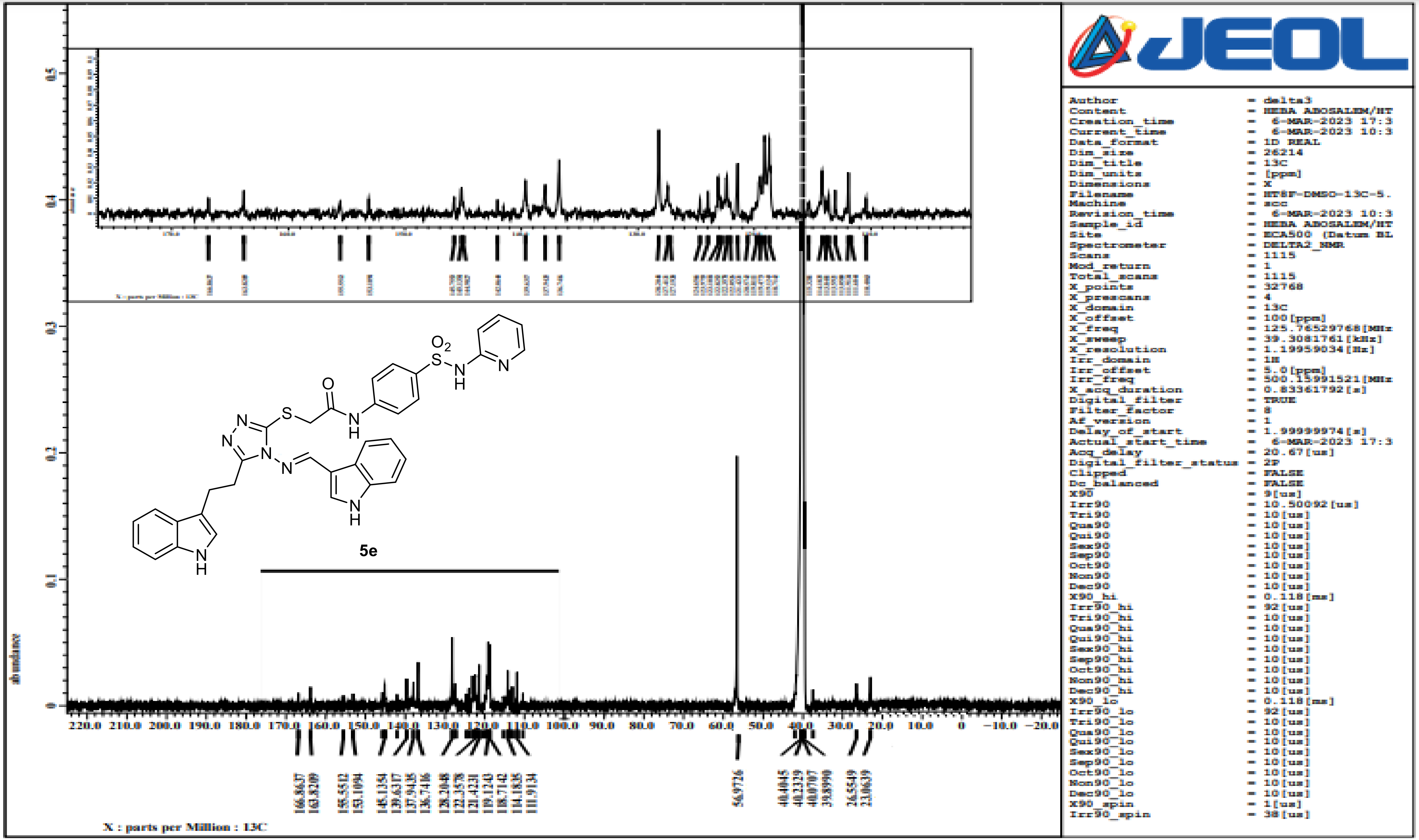
JEOL
O2
S
N
H
N
O
N
H
S
N
N
N
N
N
H
N
H
5e
abundance
X : parts per Million : 13C
166.8637
163.8209
155.5512
153.1094
145.1354
139.6317
137.9435
136.7416
128.2048
122.3578
121.4231
119.1243
118.7142
114.1835
111.9134
56.9726
40.4045
40.2329
40.0707
39.8990
26.5549
23.0639
Author = delta3
Content = HEBA ABOSALEM/HT
Creation_time = 6-MAR-2023 17:3
Current_time = 6-MAR-2023 10:3
Data_format = 1D REAL
Dim_size = 26214
Dim_title = 13C
Dim_units = [ppm]
Dimensions = X
Machine = scc
Revision_time = 6-MAR-2023 10:3
Sample_id = HEBA ABOSALEM/HT
Site = ECA500 (Datum BL
Spectrometer = DELTA2_NMR
Scans = 1115
Mod_return = 1
Total_scans = 1115
X_points = 32768
X_prescans = 4
X_domain = 13C
X_offset = 100[ppm]
X_freq = 125.76529768[MHz
X_sweep = 39.3081761[kHz]
X_resolution = 1.19959034[Hz]
Irr_domain = 1H
Irr_offset = 5.0[ppm]
Irr_freq = 500.15991521[MHz
X_acq_duration = 0.83361792[s]
Digital_filter = TRUE
Filter_factor = 8
Af_version = 1
Delay_of_start = 1.99999974[s]
Actual_start_time = 6-MAR-2023 17:3
Acq_delay = 20.67[us]
Digital_filter_status = 2P
Clipped = FALSE
Dc_balanced = FALSE
X90 = 9[us]
Irr90 = 10.50092[us]
X90_hi = 0.118[ms]
Irr90_hi = 92[us]
X90_lo = 0.118[ms]
Irr90_lo = 92[us]
X90_spin = 1[us]
Irr90_spin = 38[us]

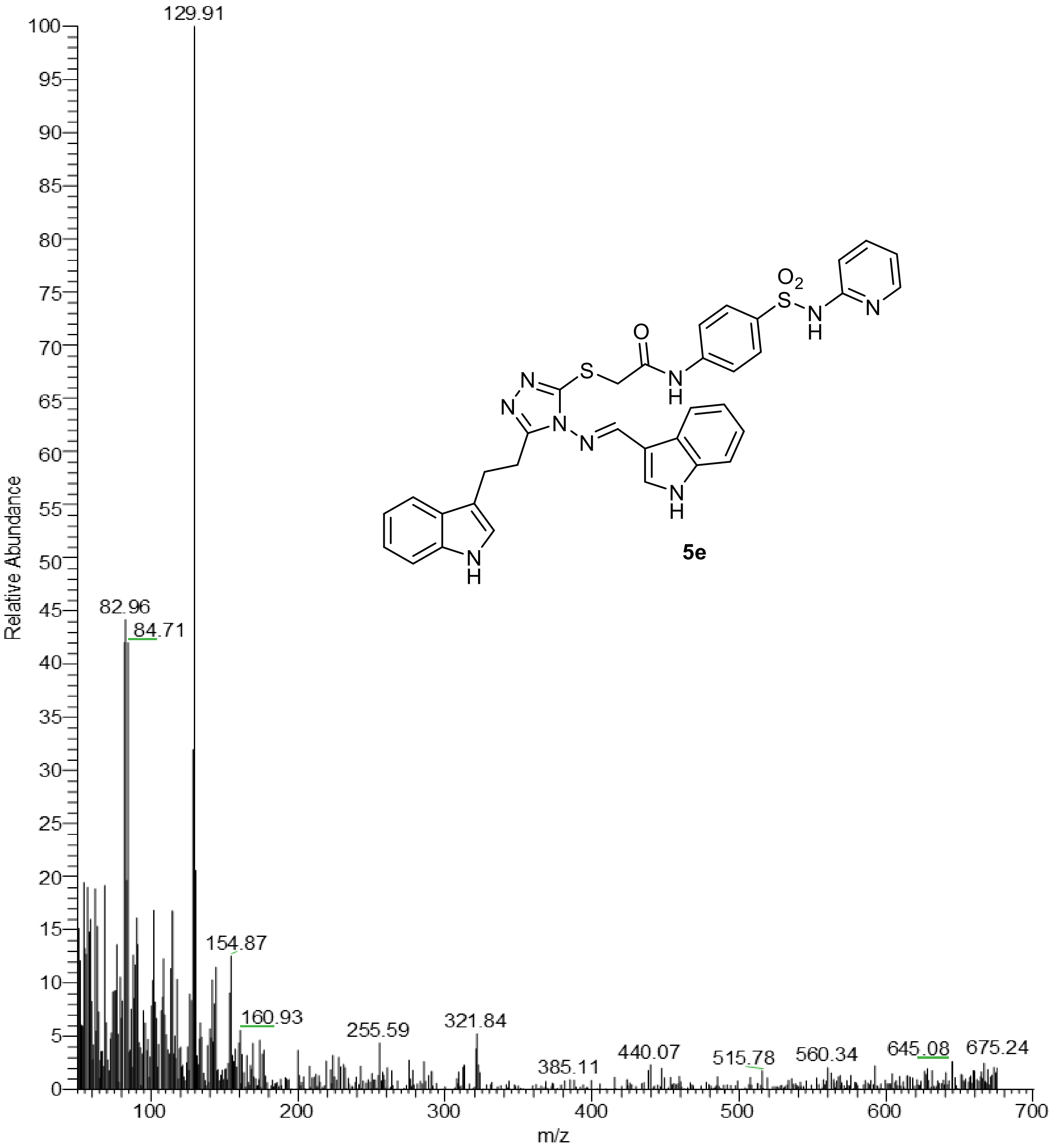

129.91
82.96
84.71
154.87
160.93
255.59
321.84
385.11
440.07
515.78
560.34
645.08
675.24
Relative Abundance
m/z
5e

Supp. Table 1. Measurement of area of migration (μm^2) of the scratched MCF-7, MDA-MB-231 and hFB cell lines after treatment with compounds 2, 3c, 4c and 5c for 0, 24h and 48h.

Mean(μm^2)±SEM/(n=3)		MCF-7	MDA-MB-231	hFB
Control	0	1136463±60357	1296438±55690	974969±10114
	24	352186±63903	516186±36064	298748±28249
	48	84328±6289	138549±24867	45369±4774
2	0	1168896±37680	1296886±98617	914300±36521
	24	614794±24582	568364±47269	517588±10745
	48	176654±3840	249961±33839	279863±4450
3c	0	1064131±31023	1296438±55690	926756±37271
	24	844828±12488	791914±59987	614236±46615
	48	565453±37291	571589±43612	208098±9066
4c	0	1136463±49697	1254796±78089	945706±20078
	24	935222±43974	945866±40617	636288±41493
	48	569640±57869	698299±53029	194179±22401
5c	0	1117334±35150	1279048±64300	974969±10114
	24	962107±63056	774689±27223	556473±24081
	48	385154±57540	530731±45573	185538±20116

Supp. Table 2: Results of cell frequencies of cell cycle phases in the various study treatment conditions

Cell Cycle Phase/ Treatment		N	MCF7		MDA	
			Mean (%)±SEM	P value	Mean (%)±SEM	P value
G0/G1	Control	6.00	60.98± 1.49		60.76 ±1.33	
	2	3.00	59.34±0.87	0.49	71.86 ±1.44	0.00
	3c	3.00	45.22± 1.79	0.00	67.37 ±1.27	0.01
	4c	3.00	49.58 ±1.68	0.00	73.16 ±1.94	0.01
	5c	3.00	51.30± 1.60	0.01	75.90± 1.35	0.00
S	Control	6.00	15.96 ±1.65		11.30 ±0.92	
	2	3.00	7.82 ±2.02	0.03	9.84 ±2.29	0.60
	3c	3.00	35.50 ± 3.87	0.02	14.30± 1.60	0.19
	4c	3.00	31.77 ±1.62	0.00	7.47 ±0.70	0.01
	5c	3.00	27.61± 1.18	0.00	8.31± 0.90	0.06
G2/M	Control	6.00	22.73 ±2.49		27.45± 0.62	
	2	3.00	32.67± 1.97	0.02	17.65 ±0.86	0.00
	3c	3.00	18.93 ±1.99	0.27	17.88 ±0.41	0.00
	4c	3.00	16.01± 0.36	0.05	11.47 ±1.55	0.00
	5c	3.00	19.13 ±0.49	0.36	14.80±0.61	0.00
SubG1	Control	5.00	0.23± 0.09		0.49 ±0.11	
	2	3.00	0.17 ±0.06	0.61	0.65 ±0.04	0.36
	3c	3.00	0.34 ± 0.09	0.43	0.45 ±0.03	0.81
	4c	3.00	2.64 ±0.41	0.02	7.90 ±0.38	0.00
	5c	3.00	1.96 ±0.06	0.04	0.99 ±0.07	0.01

Supp. Table 3. Results of proapoptotic or death inducing effects of the investigated compounds using annexin V/PI.

Viability Status	Treatment	N	MCF7		MDA	
			**Mean (%) ±SEM**	**P value**	**Mean (%) ±SEM**	**P value**
live	**control**	3.00	96.24±0.08		96.56±0.26	
	**2**	3.00	95.38±1.15	0.5	92.12± 0.10	**0.001**
	**3c**	3.00	95.41±0.13	**0.009**	92.40± 0.17	**0.000**
	**4c**	3.00	67.99±0.54	**0.000**	8.54± 0.78	**0.000**
	**5c**	3.00	93.26±0.16	0.100	80.47± 0.21	**0.000**
Early apoptotic	**control**	3.00	0.50±0.04		0.40 ± 0.08	
	**2**	3.00	1.19±0.72	0.395	3.53± 0.22	**0.002**
	**3c**	3.00	0.65±0.01	0.057	3.17± 0.17	**0.000**
	**4c**	3.00	8.69±0.22	**0.001**	62.05± 0.18	**0.000**
	**5c**	3.00	2.71±0.15	**0.003**	13.99± 0.18	**0.000**
Late apoptotic	**control**	3.00	1.60±0.08		0.58± 0.05	
	**2**	3.00	2.34±0.40	0.145	1.62± 0.07	**0.000**
	**3c**	3.00	1.77±0.03	0.168	1.40 ± 0.07	**0.001**
	**4c**	3.00	15.96±1.09	**0.000**	28.80± 1.04	**0.000**
	**5c**	3.00	3.05±0.08	**0.000**	3.86 ± 0.04	**0.000**
dead	**control**	3.00	1.66±0.02		2.45± 0.18	
	**2**	3.00	1.09±0.08	**0.015**	2.73± 0.16	0.319
	**3c**	3.00	2.17±0.09	**0.005**	3.03± 0.10	**0.050**
	**4c**	3.00	7.37±0.46	**0.000**	0.62± 0.09	**0.003**
	**5c**	3.00	0.98±0.05	**0.002**	1.67± 0.15	**0.033**

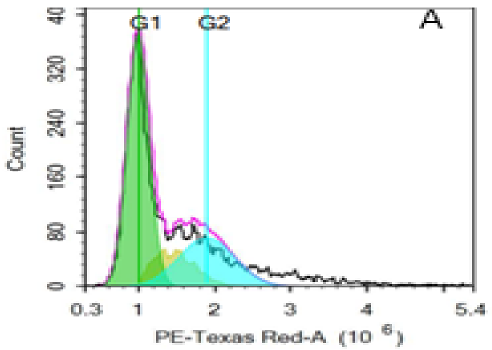

Freq G1	Freq S	Freq G2	Freq Sub-G1
60.36	15.77	29.43	0.09

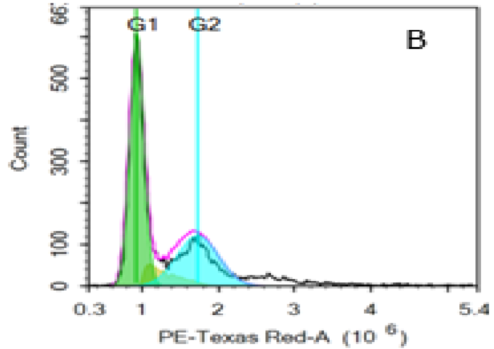

Freq G1	Freq S	Freq G2	Freq Sub-G1
60.22	8.80	35.25	0.26

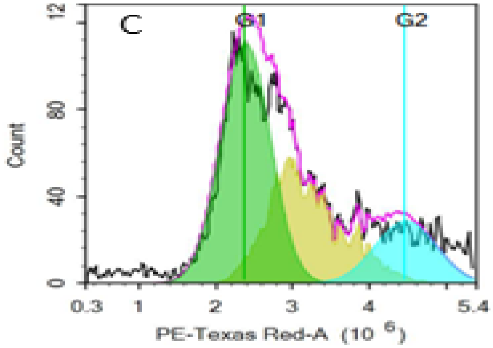

Freq G1	Freq S	Freq G2	Freq Sub-G1
52.58	34.89	16.78	2.86

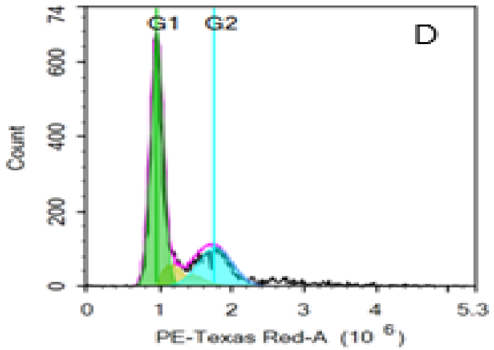

Freq G1	Freq S	Freq G2	Freq Sub-G1
67.56	11.64	28.42	0.26

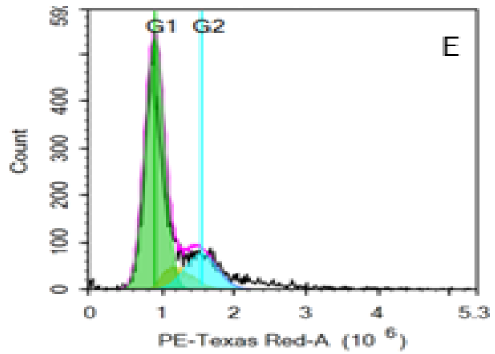

Freq G1	Freq S	Freq G2	Freq Sub-G1
82.21	10.26	19.98	0.82

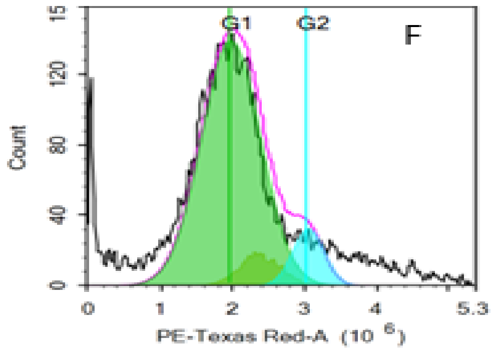

Freq G1	Freq S	Freq G2	Freq Sub-G1
71.66	5.93	8.84	7.48

Supplemental Figure 1. Cell cycle analysis by propidium iodide staining in MCF-7 and MDA-MB-231 cells. Representative flow cytometry histograms showing DNA content distribution after treatment with compounds 2 and 4c (37.5 µg/mL, 48 h). Upper panel (A-C), MCF-7 cells: (A) Untreated control displaying typical G1 predominance with minimal sub-G1 fraction; (B) Compound 2 treatment showing significant S-phase reduction and G2/M accumulation (G2/M arrest); (C) Compound 4c treatment demonstrating prominent S-phase arrest with marked sub-G1 apoptosis peak. Lower panel (D-F), MDA-MB-231 cells: (D) Untreated control with normal cell cycle distribution; (E) Compound 2 treatment inducing G1 phase accumulation; (F) Compound 4c treatment causing strong G1 arrest with substantial sub-G1 apoptotic population. Cell cycle phases are indicated: sub-G1 (apoptosis), G0/G1, S, G2/M. Quantitative analysis from n=3 independent experiments presented in Supp. Table 2.

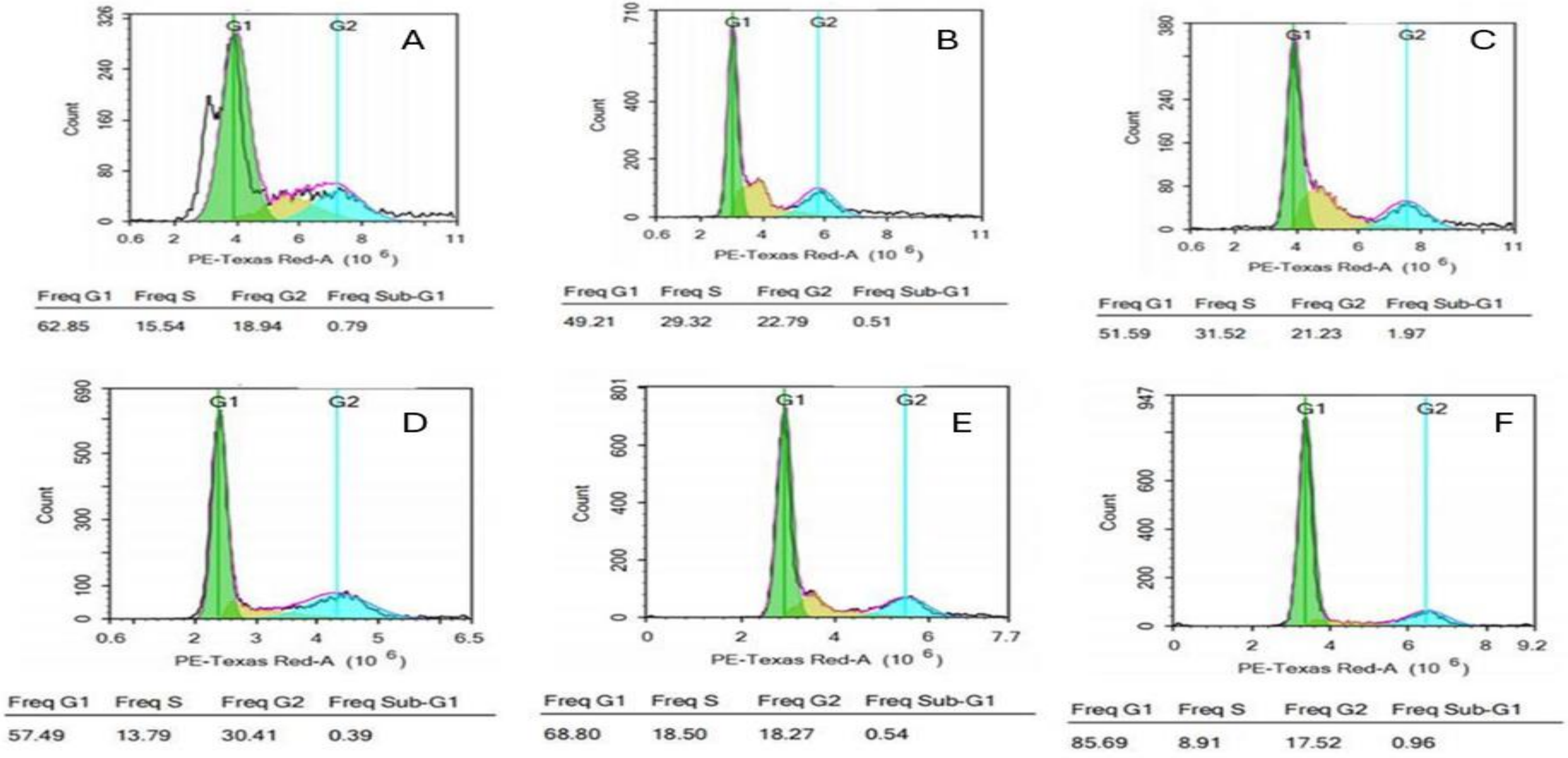

Supplemental Figure 2. Cell cycle analysis by propidium iodide staining in MCF-7 and MDA-MB-231 cells. Representative flow cytometry histograms showing DNA content distribution after treatment with compounds 3c and 5c (37.5 µg/mL, 48 h). Upper panel (A-C), MCF-7 cells: (A) Untreated control displaying typical G1 predominance with minimal sub-G1 fraction; (B) Compound 3c treatment inducing S-phase arrest with minimal sub-G1 apoptosis; (C) Compound 5c treatment demonstrating S-phase arrest with detected sub-G1 apoptotic population. Lower panel (D-F), MDA-MB-231 cells: (D) Untreated control with normal cell cycle distribution; (E) Compound 3c treatment showing modest G1 phase accumulation (weakest G1 arrest effect); (F) Compound 5c treatment causing significant G1 arrest with elevated sub-G1 apoptotic fraction. Cell cycle phases indicated: sub-G1 (apoptosis), G0/G1, S, G2/M. Quantitative analysis from n=3 independent experiments presented in Supp. Table 2.

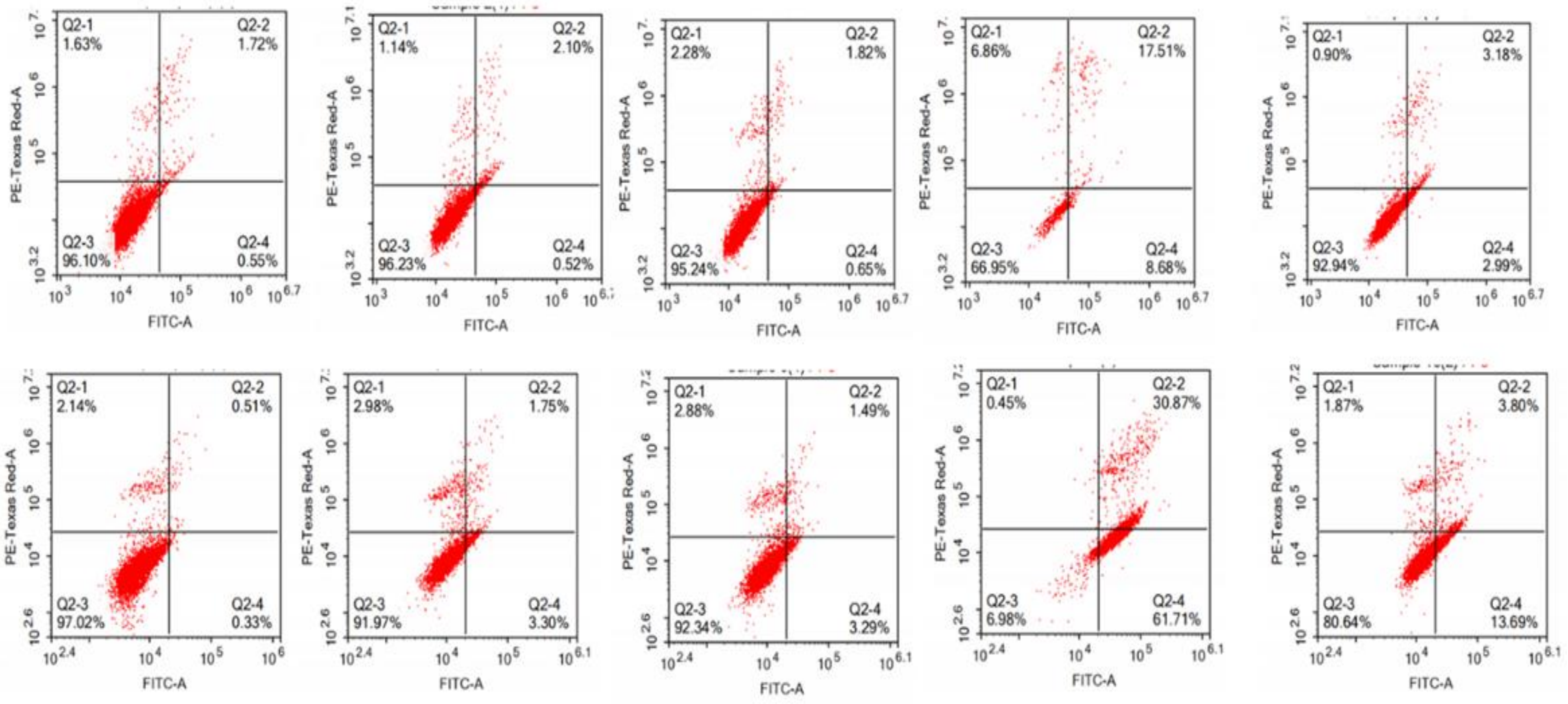

Supplemental Figure 3. Induction of apoptosis by 1,2,4-triazolo-indolyl conjugates in breast cancer cell lines. Representative Annexin V/PI flow cytometry dot plots for MCF-7 (non-TNBC, upper panel) and MDA-MB-231 (TNBC, lower panel) cells after 48 h treatment with compounds 2, 3c, 4c, 5c or left untreated (control).
